# Synthesis of Pyrrolo[3,4-*b*]pyridin-5-ones via Ugi–Zhu Reaction and In Vitro–In Silico Studies against Breast Carcinoma

**DOI:** 10.3390/ph16111562

**Published:** 2023-11-06

**Authors:** Ivette Morales-Salazar, Carlos E. Garduño-Albino, Flora P. Montes-Enríquez, Dania A. Nava-Tapia, Napoleón Navarro-Tito, Leonardo David Herrera-Zúñiga, Eduardo González-Zamora, Alejandro Islas-Jácome

**Affiliations:** 1 Departamento de Química, Universidad Autónoma Metropolitana-Iztapalapa, San Rafael Atlixco 186, Col. Vicentina, Iztapalapa, Ciudad de México 09340, Mexico; ivette649_tatu@hotmail.com (I.M.-S.); charlywuiller199912101@gmail.com (C.E.G.-A.); montesenriquez98@outlook.com (F.P.M.-E.); egz@xanum.uam.mx (E.G.-Z.); 2 Laboratorio de Biología Celular del Cáncer, Universidad Autónoma de Guerrero, Chilpancingo de los Bravo 39086, Mexico; danianavatapia@uagro.mx

**Keywords:** breast cancer, docking, MCRs, Ugi–Zhu, Pyrrolo[3,4-*b*]pyridin-5-ones, MDA-MB-231, MCF-7, molecular dynamics

## Abstract

An Ugi–Zhu three-component reaction (UZ-3CR) coupled in a one-pot manner to a cascade process (*N*-acylation/*aza* Diels–Alder cycloaddition/decarboxylation/dehydration) was performed to synthesize a series of pyrrolo[3,4-*b*]pyridin-5-ones in 20% to 92% overall yields using ytterbium triflate as a catalyst, toluene as a solvent, and microwaves as a heat source. The synthesized molecules were evaluated in vitro against breast cancer cell lines MDA-MB-231 and MCF-7, finding that compound **1f**, at a concentration of 6.25 μM, exhibited a potential cytotoxic effect. Then, to understand the interactions between synthesized compounds and the main proteins related to the cancer cell lines, docking studies were performed on the serine/threonine kinase 1 (AKT_1_) and Orexetine type 2 receptor (Ox_2_R), finding moderate to strong binding energies, which matched accurately with the in vitro results. Additionally, molecular dynamics were performed between proteins related to the studied cell lines and the three best ligands.

## 1. Introduction

Breast cancer is the most common invasive neoplasm and the leading cause of death in women worldwide. According to the World Health Organization, 2.5 million new cases and 685,000 deaths were reported worldwide in 2020 [1]. Furthermore, it has been described that 90% of cancer deaths are associated with metastasis, and breast cancer cells spread mainly to secondary sites such as the bones, lungs, liver, brain, and lymph nodes [2]. One of the most common and widely accepted classifications of breast cancer is related to the expression of estrogen receptor (ER), progesterone receptor (PR), and human epidermal growth factor receptor 2 (HER2) [3,4]. According to this classification, breast cancer has been divided into six main subtypes: luminal A, luminal B, basal-like or triple-negative breast cancer (TNBC), basal-epithelial, normal-like, and claudin-low breast cancer [3,5]. Currently, the molecular characteristics of breast cancer have allowed the use of different cellular models as a first approach in the research of alternative or complementary treatments in breast cancer therapy, where cell lines such as MCF-7 (luminal subtype A) and MDA-MB-231 (TNBC subtype) are frequently used [6], for instance, in the present work.

Thus, to investigate the possible therapeutic effects of the synthesized pyrrolo[3,4-*b*]pyridin-5-ones **1a**–**1k**, molecular docking assays were performed using 20 protein targets related to breast cancer: Serine/Threonine-protein kinase AKT (AKT_1_), Troponin cardiac muscle (cTn), Cytochrome P450 2C9 (CYP2C9), Cytochrome P450 3A4 (CYP3A4), MAP kinase p38 alpha (p38α), Cathepsin S (CTSS), Cyclin-dependent kinase 2/cyclin A (Cdk2/CA), Insulin-degrading enzyme (IDE), Cyclin-dependent kinase 2/cyclin E (Cdk2/CE), Cyclin-dependent kinase 4 (CDK4), Mitogen-activated protein kinase 8 (MAPK8), Cathepsin L (CTSL), Serotonin 6 receptor (5-HT_6_), Dual specificity protein phosphatase 3 (DUSP3), GTPase Kras (KRAS), MAP kinase-activated protein kinase 3 (MAPK3), Metabotropic glutamate receptor 2 (mGluR2), Ghrelin receptor (ghrelinR), Histamine H3 receptor (H3), and Orexin-2 receptor (Ox_2_R). The best hits were confirmed using molecular dynamics (MD). Based on available information, there are no prior investigations into the potential of this compound series to target AKT_1_ [7,8] and Ox_2_R [9,10] as the main proteins relating to breast cancer. Compounds **1f**, **1h**, and **1k** bind efficiently to catalytic sites of the two specific target proteins implicated in the proliferation of breast cancer cells. As a result, it can be observed that compounds **1f**, **1h**, and **1k** possess advantageous structural characteristics, rendering them highly promising molecules with prospective therapeutic applications in antineoplastic treatments for breast cancer.

## 2. Results and Discussion

### 2.1. Synthesis

Multicomponent reactions are privileged synthetic tools due to the high atom economy observed when assembling polyheterocyclic cores in a one-pot manner [11]. Within MCRs, an elegant variation of the Ugi three-component reaction (U-3CR) called the Ugi–Zhu reaction (UZ-3CR) has been extensively developed in our research group to synthesize a broad variety of polyheterocyclic pyrrolo[3,4-*b*]pyridin-5-ones [12].

**Scheme 1:**
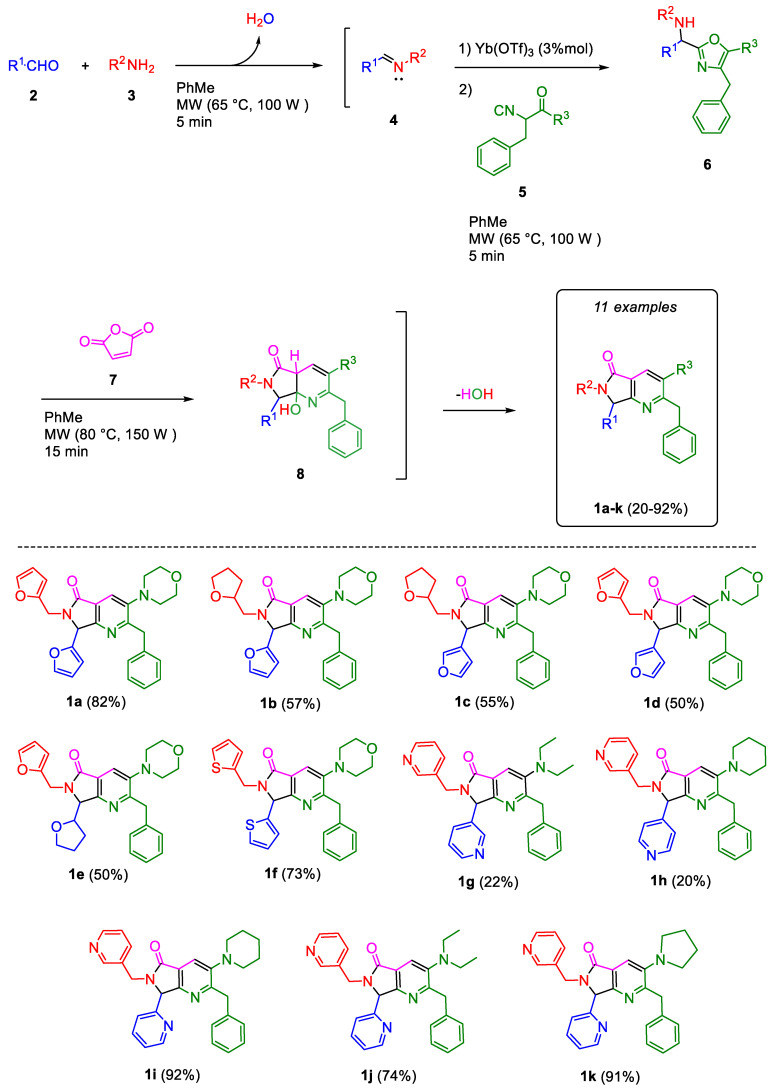
Synthesis of pyrrolo[3,4-*b*]pyridin-5-ones **1a**–**1k**.

Thus, to achieve pyrrolo[3,4-*b*]pyridin-5-ones **1a**–**1k**, a one-pot process was performed by coupling an UZ-3CR to a cascade sequence: *aza* Diels–Alder/*N*-acylation/aromatization (decarboxylation/dehydration). In some of our previous reports, it was found that toluene and ytterbium triflate were the best solvent and catalyst, respectively, to perform this process [13,14]. Thus, it was necessary to synthesize isocyanoacetamides from racemic phenylalanine in three steps as reported by Zhu and co-workers: (1) amino acid *N*-formylation, (2) peptidic coupling, and (3) Ugi-type dehydration [15]. With isocyanides in hand, and according to the Ugi–Zhu process, the reaction between aldehydes **2** and amines **3** yielded the imines **4**, which were activated with Lewis acid catalysts to promote α-nucleophilic attack by α-isocyanoacetamides **5**. Then, through a non-prototropic chain-ring tautomerization, 5-aminooxazoles **6** were afforded. Thus, addition of maleic anhydride (**7**) promoted an *aza* Diels–Alder cycloaddition followed by the *N*-acylation/decarboxylation/dehydration cascade process to give the products **1a**–**1k** in moderate to good yields (20–92%) (Scheme 1). It is worth highlighting that only three small molecules (two molecules of water and one molecule of CO_2_) were released in all synthetic processes, demonstrating the process’s high atom economy under ecofriendly conditions.

### 2.2. Anticancer Activity

**Figure 1:**
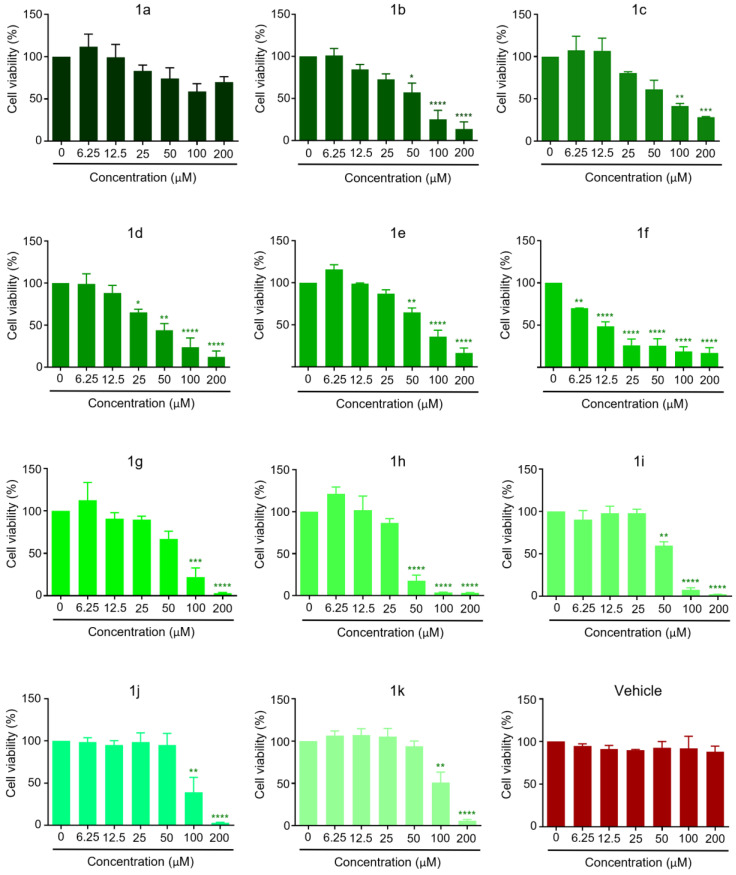
Effect of compounds **1a**–**1k** on cell viability of MDA-MB-231 cells. The MDA-MB-231 cell line was exposed to 0, 6.25, 12.5, 25, 50, 100, and 200 µM of the chemical compounds for 48 h. The graphs are representative of the % cell viability concerning the control and are representative of three independent experiments. Statistical significance was calculated by ANOVA and Dunnett, ±SD with values of * *p* < 0.05, ** *p* < 0.01, *** *p* < 0.001, and **** *p* < 0.0001 indicating statistical significance.

**Figure 2:**
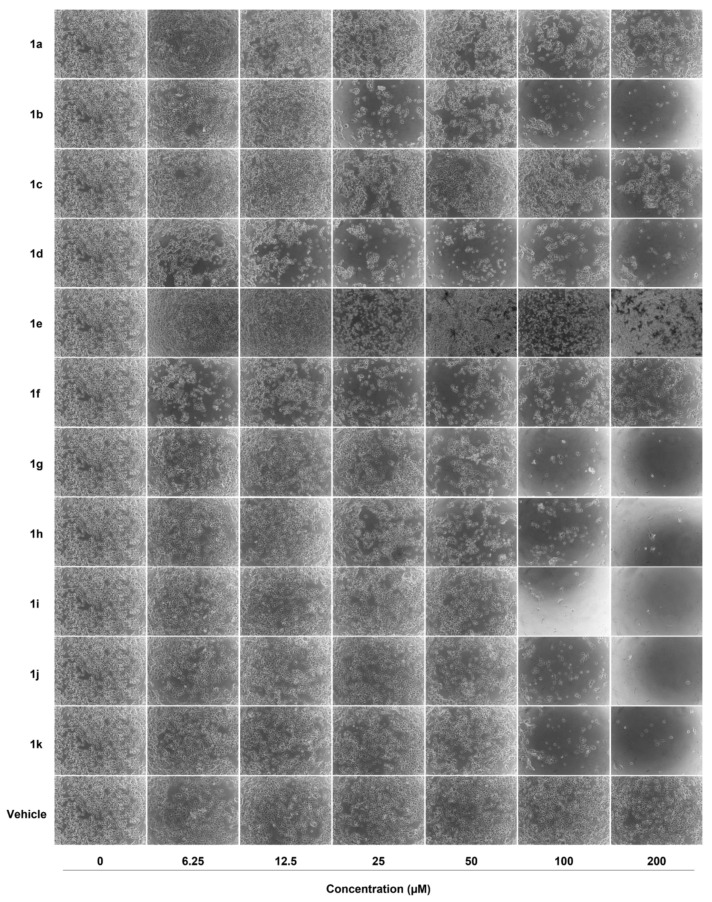
Effect of compounds **1a**–**1k** on the cell viability of MDA-MB-231 cells. Representative phase contrast microscopy images of MDA-MB-231 tumor cells. The cells were treated with different concentrations of the chemical molecules (0, 6.25, 12.5, 25, 50, 100, and 200 µM) or vehicle (DMSO) for 48 h. Images of morphological changes were taken at 48 h with a 10× objective.

The effect of compounds **1a**–**1k** was evaluated on cell viability by MTT assays of two breast cancer cell lines, the triple-negative cell line MDA-MB-231 and the HER2-positive breast cancer line MCF-7. Results show that in the MDA-MB-231 cell line, the compounds that significantly decrease cell viability at lower concentrations are **1f** (6.25 µM) and **1d** (25 µM), whereas compounds **1b**, **1e**, **1h**, and **1i** decrease cell viability at 50 µM. Compounds **1c**, **1g**, **1j**, and **1k** decrease viability starting at 100 and 200 µM. Interestingly, no significant decrease was observed for compound **1a** in MDA-MB-231 breast cancer cells (Figure 1 and Figure 2).

In particular, in the MCF-7 breast cancer cell line, compound **1i** decreased cell viability at 50 μM, **1h** at 100 μM, and **1b**, **1c**, **1d**, **1g**, **1j**, and **1k** at 200 μM. However, compounds **1a**, **1e**, and **1f** had no significant effect on cell viability.

**Figure 3:**
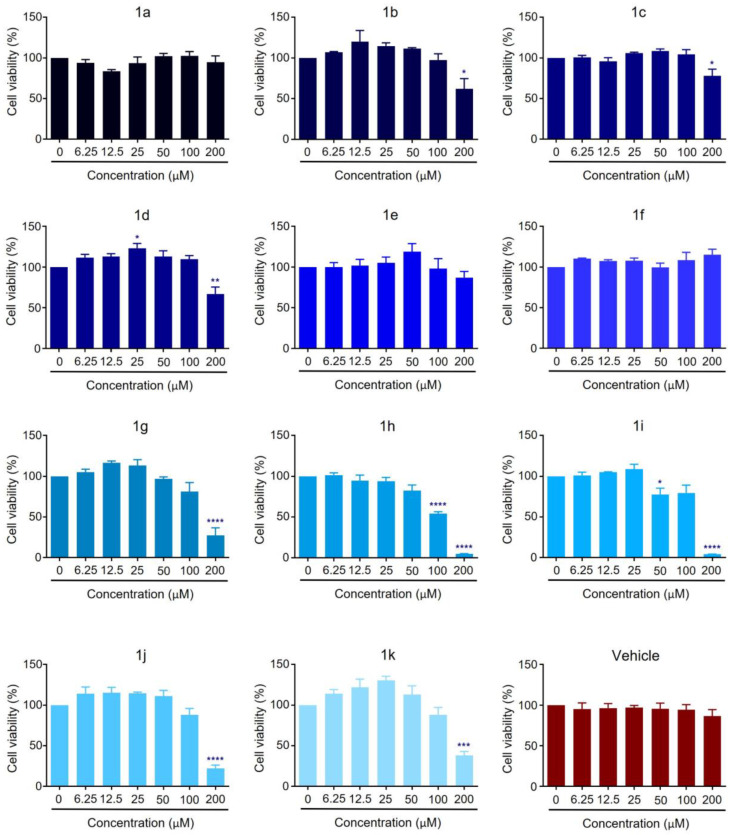
Effect of chemical compounds **1a**–**1k** on cell viability of MCF-7 cells. The MCF-7 cell line was exposed to 0, 6.25, 12.5, 25, 50, 100, and 200 µM of the compounds for 48 h. The graphs are representative of the % cell viability concerning the control and are representative of three independent experiments. Statistical significance was calculated by ANOVA and Dunnett, ±SD with values of * *p* < 0.05, ** *p* < 0.01, *** *p* < 0.001, and **** *p* < 0.0001 considered statistically significant.

**Figure 4:**
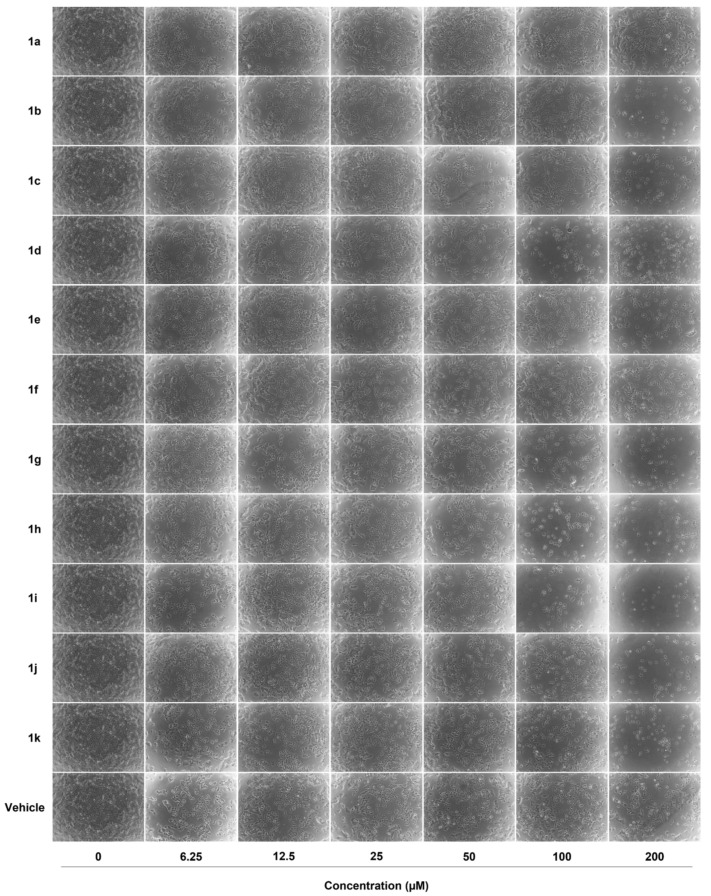
Effect of chemical compounds **1a**–**1k** on cell viability of MCF-7 cells. Representative bright-field microscopy images of MCF-7 tumor cells. Cells were treated with different concentrations of the chemical molecules (0, 6.25, 12.5, 25, 25, 50, 100, and 200 µM) or vehicle (DMSO) for 48 h. Images of the morphological changes at 48 h were taken with a 10× objective on an inverted microscope.

When photographs were taken of MCF-7 cells treated with different concentrations of the compounds **1a**–**1k**, effects were observed only at 100 and 200 μM with **1b**, **1c**, **1e**, **1g**, **1h**, **1i**, **1j**, and **1k** (Figure 3 and Figure 4). Interestingly, unlike MDA-MB-231 cells, where we observed effects from low concentrations, the effects in the MCF-7 cell line were seen only at high concentrations.

With the data obtained from the cell viability assays of the breast cancer cell lines MDA-MB-231 and MCF-7, the IC_50_ values were determined for all synthesized compounds with ranges from 0 to 200 µM. Of all the compounds tested, the lowest IC_50_ doses in MDA-MB-231 cells were for compounds **1f**, **1i**, and **1d** (Table 1). Interestingly, no significant effects were observed with any compound in the MCF-7 breast cancer cell line. These data suggest that compound **1f** has the most anticancer activity and that it is specific for MDA-MB-231 triple-negative breast cancer cells.

The importance of assessing cell viability in MDA-MB-231 and MCF-7 cancer cells treated with different concentrations of compounds **1a**–**1k** is due to the presence in their chemical structure of the pyrrolo[3,4-*b*]pyridin-5-one series, which is a fused-type polyheterocyclic system and is an *aza*-analogue of the isoindoline-1-one core [16]. Isoindolin-1-one has been described as the structural core of several natural and synthetic anticancer agents [17]. Moreover, in various recently published works, isoindolin-1-one related heterocyclic compounds have been tested against cancer cell lines [18,19,20,21]. In this sense, it was likely that the compounds could have biological activity by decreasing viability of mammary cancer cells +ER and +PR, and TNBC cells. Interestingly, in our study, the compounds tested had different effects depending on the cell line. Indeed, it was found that the compound with the highest biological activity in TNBC MDA-MB-231 cells was **1f**, decreasing cell viability from the lowest concentration used (6.25 µM). According to the structural substituents of all molecules, compound **1f** is the only one with sulfur atoms in its structure. Different studies have reported that sulfur is present in many compounds with biological activities, including anticancer properties, because it decreases cell proliferation and, therefore, metastasis in various cancer cell lines such as prostate cancer, murine colorectal cancer, and lung carcinoma [22,23].

On the other hand, an analysis was also performed with compounds **1a**–**1k** using the SwissTargetPrediction (Ver. 2019) and PASS-Protein-Target (Ver. 2.0) platforms. It was found that compound **1f** may interact with proteins such as PI3K, which is a key regulator in growth, progression, survival, metabolism, protein synthesis, and angiogenesis of breast cancer; likewise, it can also participate in the risk of resistance to endocrine therapy and chemotherapy [24]. MAPKs are another essential family of proteins related to tumor progression [25,26]. Likewise, **1f** can interact with CDK4, one of the regulatory proteins of cell cycle progression [27]. Indirectly, it can also interact with hypoxia-inducible factor-1-α (HIF-1-α), which facilitates the adaptation of cancer cells to hypoxic conditions, allowing tumor cells to survive during metabolic stress and enter a prolonged state of tumor dormancy [28].

### 2.3. In Silico Studies

#### 2.3.1. Multi-Target Molecular Docking

The use of molecular docking simulations with organic compounds as inhibitors against numerous proteins is well known and often applied in both industrial and laboratory contexts. The purpose of these simulations is to minimize temporal intervals, decrease costs, and optimize research endeavors [29,30,31]. In the present investigation, molecular docking and MD techniques were used to ascertain and to authenticate inhibitors targeting diverse protein targets. A screening method was performed to identify possible protein targets for the compounds **1a**–**1k** using SwissTargetPrediction and PASS-Protein-Targets [32,33,34,35]. A total of 20 potential targets were identified in this study (Table S2, electronic Supplementary Material), each with a probability (P) greater than 0.8. Among these protein targets, 15 were found to be water-soluble proteins, including AKT_1_ (PDB-ID: 5KCV), CYP2C9 (PDB-ID: 1R9O), CYP3A4 (PDB-ID:8EXB), p38α (PDB-ID: 6SFI), CTSS (PDB-ID: 2FRA), Cdk2/CA (PDB-ID: 2R3F), Cdk2/CE (PDB-ID: 7E34), IDE (PDB-ID: 6EDS), CDK4 (PDB-ID: 2W9Z), MAPK8 (PDB-ID: 5IU2), CTSL (PDB-ID: 8C77), DUSP3 (PDB-ID: 3F81), MAPK3 (PDB-ID: 3FHR), KRAS (PDB-ID: 7SCX), and cTn (PDB-ID 4Y99). Additionally, five membrane proteins were identified as potential targets, namely 5-HT_6_ (PDB-ID: 7XTB), mGluR2 (PDB-ID: 7EPF), ghrelinR (PDB-ID: 7NA8), H3 (PDB-ID: 7F61), and Ox_2_R (PDB-ID: 6TPN).

A thorough target analysis demonstrates that pyrrolo[3,4-*b*]pyridin-5-one could interact with a significant number of proteins implicated in cancer-related disorders. In this context, it is plausible that compounds **1a**–**1k** may exhibit inhibitory properties akin to those observed in antineoplastic drugs. Therefore, these compounds may be identified as potential candidates for utilization in breast cancer therapy.

**Figure 5:**
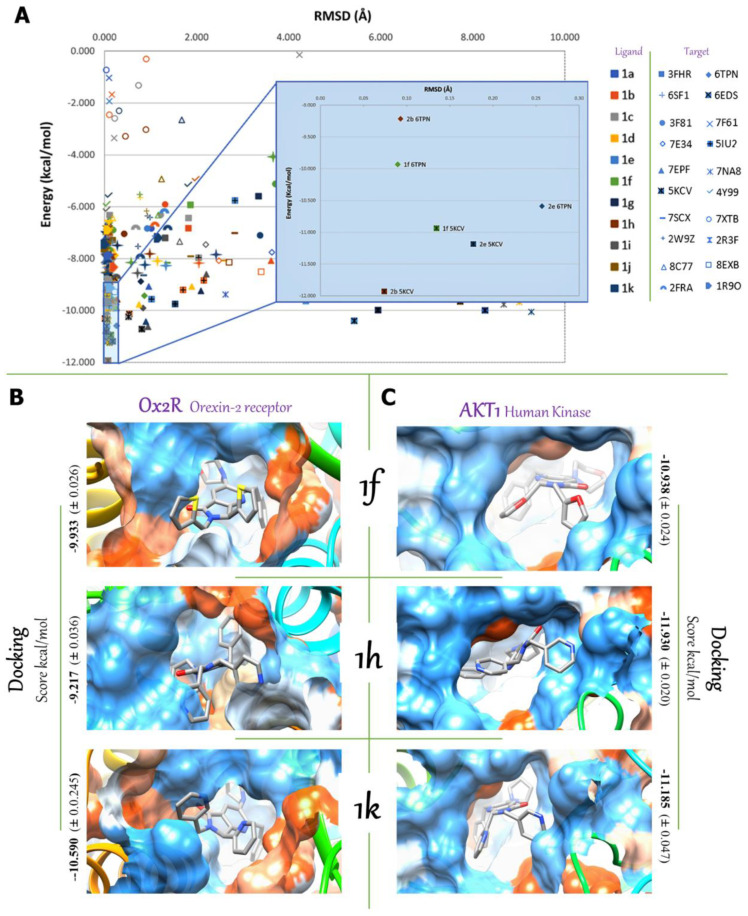
Docking results: (**A**) energy vs. RMSD, (**B**) docking to Ox_2_R, (**C**) docking to AKT_1_.

Breast cancer, a prominent contributor to female cancer-related mortality, frequently exhibits metastatic tendencies, disseminating across several anatomical sites. The primary aim of the computational research performed herein is to ascertain efficient approaches for identifying molecules exhibiting optimal binding possesses, utilizing docking scores as a basis for the evaluation. The task of determining the appropriate conformation of a ligand–protein complex in a computational molecular framework is accompanied by difficulties related to different docking techniques [36,37,38,39,40,41,42]. To investigate this matter, compounds **1a**–**1k** were subjected to individual docking simulations against each of the 20 possible protein therapeutic targets. The procedure encompassed the application of three scoring functions, afterwards followed by the re-evaluation of docking through the utilization of Autodock Vina. The findings unveiled a spectrum of interaction score energy values for all compounds, ranging from −7.00 to 12.00 kcal/mol (Table S3). The docking clusters are depicted in Figure 5A, wherein the lower-left quadrant contains the most advantageous binding pose. These poses are chosen based on the lowest root mean square deviation (RMSD), lowest energy score, and high cluster populations.

Then, predictions were made regarding the ADMETox characteristics of compounds **1a**–**1k**. This analysis involved the utilization of models that incorporate medicinal chemistry features, pharmacokinetics, drug likeness, and toxicity profiles. The analysis was performed using machine-learning techniques included in Protox-II and SwissADME. In the methodology for performing MD simulations, we opted for molecules that demonstrated favorable docking scores, desirable drug-like characteristics (Table S4), and low IC_50_ values. As a result, compounds **1f**, **1h**, and **1k** were found to meet these criteria and demonstrated the highest positive rankings upon evaluation against the therapeutic targets Ox_2_R and AKT_1_.

AKT_1_ has been identified as a crucial regulator of key aspects of cancer and metastasis, resulting in a growing interest in its potential as a therapeutic target [43,44]. In addition, the OX_2_R receptor, which exhibits an absence of expression in healthy cells but is present in breast cancer cells, has emerged as a highly promising contender for targeted cancer therapy. The complete investigation of its possible significance in cancer cells must be undertaken. A thorough comprehension of these functions may be pivotal for advancing breast cancer therapy [10,45,46].

The main structure of AKT_1_ (UniProt: P31749) consists of two structural domains (Figure S24): the first encompasses a Pleckstrin Homology (PH) domain, spanning residues 6 to 105. The second domain includes residues 150 to 447 and is characterized by a conserved kinase domain. The *N*-Lobe (residue 144 to 230) and C-Lope motifs (residue 230 to 444), along with the G-loop (residue 157 to 162) and the activation loop (residue 350 to 360), come together to form this kinase domain. Our research primarily investigated the allosteric cavity generated between the PH and kinase domains. The presence of an allosteric pocket can confer benefits to our compounds in relation to AKT_1_ [44]. The utilization of an allosteric pocket provides a distinctive advantage over ATP-competitive inhibitors. Ox_2_R (UniProt: O43614) is composed of seven helical transmembrane segments (TM1 to TM7) that are interconnected by three intracellular loops (ICL1 to ICL3) and three extracellular loops (ECL1 to ECL3). The Ox_2_R variant with a truncated intracellular loop 3 (ICL3) was employed in this investigation (Figure S25), and the Ox_2_R target was constructed using the structural data provided by Asada et al. in 2022 (PDB-ID: 7XRR).

According to the docking results (Table S3) the compounds **1f**, **1h**, and **1k** had the highest evaluation with the lower interaction score energy of −9.933, −9.217, and −10.59 kcal/mol, respectively, when evaluated against Ox_2_R. Similarly, these compounds achieved scores of −10.938, −11.930, and −11.185 kcal/mol, respectively, when assessed against AKT_1_. The findings from in silico molecular coupling investigations targeting AKT_1_ indicate that compounds **1f**, **1h**, and **1k** have potential as tyrosine kinase allosteric inhibitors (AKT_1_-aIs). In the same manner, the aforementioned compounds exhibit inhibitory properties towards Ox_2_R, a GPCR receptor that is uniquely expressed in breast cancer cells and not in non-tumor cells [10,46], indicating that Ox_2_R has significant potential as a target for cancer treatment. Figure 5B displays the best docking pose inside the active sites of AKT_1_ and Ox_2_R with a conformational plasticity in the pocket. To date, our research has shown the potential of compounds **1f**, **1h**, and **1k** as viable candidates with pro-apoptotic and antiproliferative properties, with notable low IC_50_ values. The ADMETox evaluation (Table S4) of compound **1f** revealed that it exhibits favorable characteristics compared to compounds **1h** and **1k**. Furthermore, compound **1k** displayed a higher toxicity than compound **1h**. However, it is crucial to emphasize that compound **1k** exhibits the most advantageous MCF-7/MDA-MB-231 IC_50_ ratio, which is valued at 1.59. The importance of this ratio lies in its capacity to enable the identification of compounds that have the potential to operate as anticancer agents, capable of affecting both cell lines at a lower dosage. In this way, **1f** has been evaluated and classified as a Class V chemical, indicating that it has the potential to cause damage if ingested, with an LD_50_ that falls within the range of 2000 to 5000 mg/kg, indicating moderate toxicity. Furthermore, it exhibits a high level of absorption in the gastrointestinal tract, complies with Lipinski’s rule of five, and does not penetrate the blood–brain barrier. In contrast, it is possible for the compounds **1h** and **1k** to overcome this barrier and be categorized as Class III drugs, which are specifically identified as “toxic if consumed” (50 < LD_50_ ≤ 300). Regarding the ligand endpoints targeting toxicity, nuclear receptors, and stress pathways, it is projected that all three compounds will exhibit inactivity.

Figure 5A illustrates clustering graphs that indicate distinct groupings for each protein–ligand system. Each point within the graphs represents an individual cluster. The figure shows a magnified depiction of the energy scores that are optimally positioned in relation to the RMSD of the clusters with the highest population. Significantly, the interactions of **1f**, **1h**, and **1k** with AKT_1_ and Ox_2_R demonstrate a strong correlation between scoring energy and RMSD, suggesting a substantial binding affinity. Figure 5B depicts the spatial arrangements of **1f**, **1h**, and **1k** within the cavities of the Ox_2_R and AKT_1_ target proteins. The energy scores associated with each molecule are presented alongside their respective orientations. The atom–ligand pairs are visually distinguished by color, as carbon is shown as gray, nitrogen as blue, oxygen as red, and sulfur as yellow. The region surrounding the ligands within the pocket is primarily characterized by hydrophobic properties, as indicated by the blue color. Conversely, the remaining surface of the pocket exhibits polar characteristics, represented by the orange color. Nevertheless, the depth of the pocket is of such a magnitude that it poses a considerable challenge in terms of visualizing the entirety of its surface. By directing attention only to the pocket region, a more distinct exterior perspective can be achieved.

#### 2.3.2. Molecular Dynamic Simulation Studies of **1f**, **1h**, and **1k** on AKT_1_ and Ox_2_R

Thus far, it has been established that compounds **1f**, **1h**, and **1k** demonstrate positive binding scores with AKT_1_ and Ox_2_R target proteins, together with moderately hazardous IC_50_ values at experimentally determined inhibitory dosages. To determine the accurate conformations of the ligands and examine the impact of conformational alterations on side chains during recognition processes, as well as to obtain a deeper understanding of the interaction energies and mechanisms that govern the interactions between ligands and the target proteins, a series of three individual simulations (replicas) were performed for each ligand/protein system. Each simulation had a duration of 100 ns. To ensure the representativeness of the population for energy calculations, we combined the final 80 ns of each replica. Following that, we conducted a clustering analysis to categorize conformational states that exhibited similarity in the simulations and so minimize structural variation.

**Figure 6:**
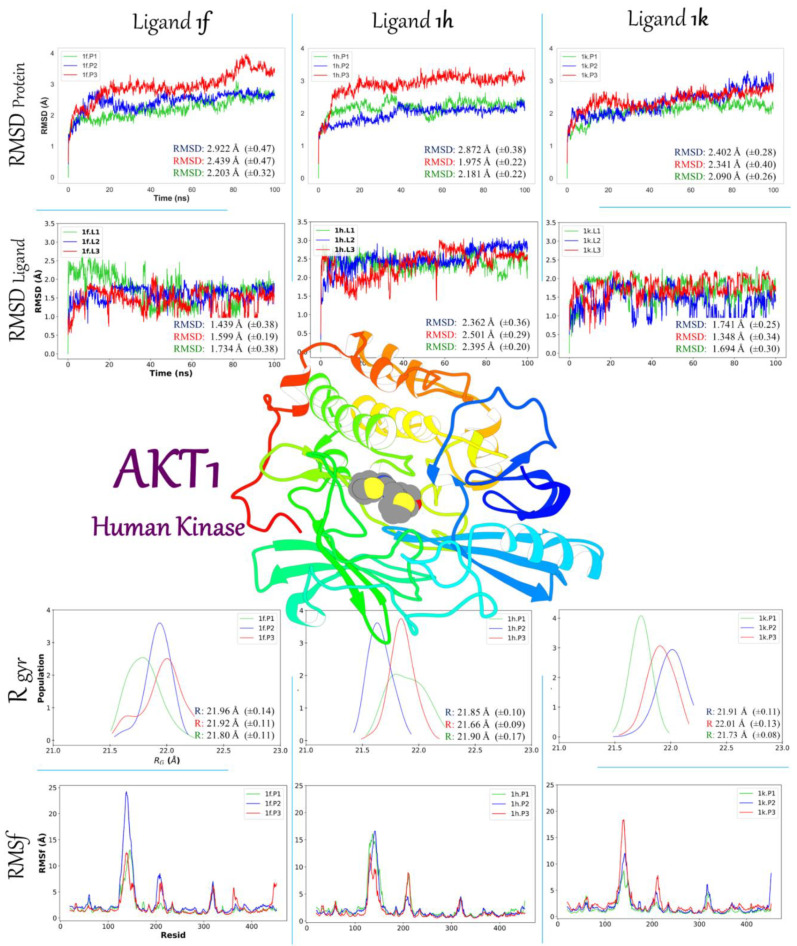
MD structure equilibrating descriptors for the interactions **1f**, **1h**, and **1k** with AKT_1_.

**Figure 7:**
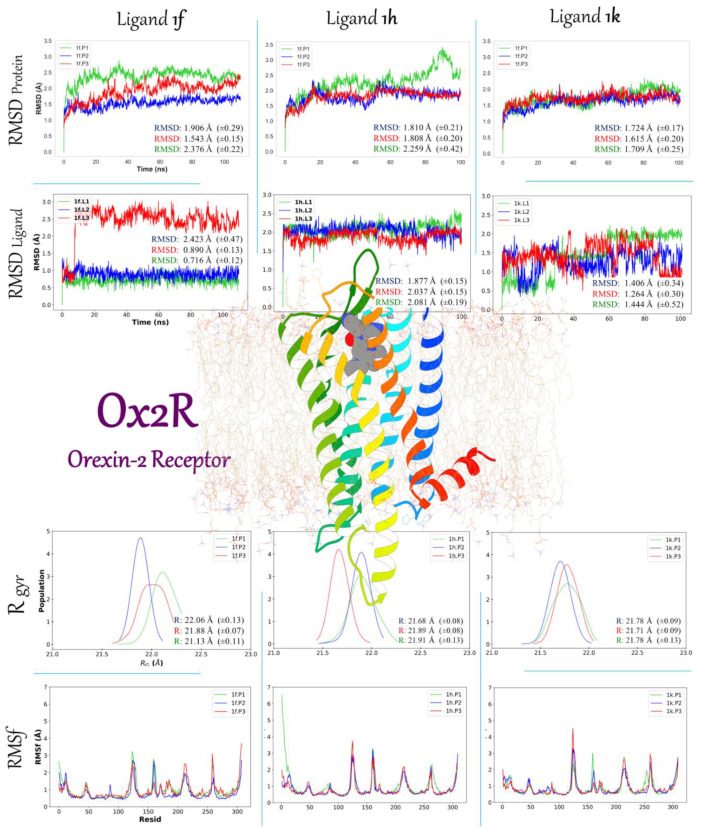
Structural equilibrating descriptors obtained by MD simulations for the interactions **1f**, **1h,** and **1k** with the Ox_2_R receptor.

The RMSD findings, as depicted in Figure 6 and Figure 7, indicate that the simulations attain stability starting from 20 ns, apart from one of the three replicas performed for the **1f**/AKT_1_ system (Figure 6, blue line), **1k**/AKT_1_ system (Figure 6, red line), and **1h**/Ox_2_R system (Figure 7, green line). This observation suggests that these replicas may have experienced either a lack of stabilization or demonstrated more significant variations in comparison to the remaining two replicas. In this context, it is shown that systems incorporating the AKT_1_ protein exhibit RMSD values below 4 Å, while systems containing Ox_2_R demonstrate RMSD values below 3.5 Å (Table S5). Consequently, all trajectories were incorporated into the clustering procedure. Similar trends were detected in a ligand RMSD study, akin to those observed in protein RMSD analysis. As illustrated in Figure 6, compound **1k** exhibits fluctuations that are correlated with those of AKT_1_. Compound **1f** displays an imbalance noticed in the final 20 ns that matches with the sudden increase in the protein RMSD. With respect to the second replica of **1f**/Ox_2_R, as seen by the blue line in Figure 7, it is evident that **1f** experiences a displacement, which is likely attributable to a rotating motion occurring within the binding site. No ligand RMSD systems surpassed 3 Å. Hence, despite the presence of noticeable visual variations in certain replicas, the ligands consistently maintained their position within the binding pockets. It is worth mentioning that there is an observable association between the movements of ligands within the cavity and the mechanical motions of the protein. This implies the presence of a useful stimulus–response relationship.

The graphical representations (Figure 6) within the study illustrate the protein–RMSD (alpha-carbon), ligand–RMSD, root mean square fluctuations (RMSf), and normalized distribution of the radius of gyration (R_gyr_) across a simulated time period of 100 ns in each replicate of every one of the systems. The replicates are distinguished by distinct colors, with replicate 1 represented by blue, replicate 2 denoted by red, and replicate 3 indicated by green. To minimize the possibility of incorrect interpretation, the first graph for each descriptor is provided with axis labels. The central region of the diagram illustrates the AKT_1_ target protein’s structure using cartoon representations. Additionally, the **1h** ligand has been added into the allosteric binding pocket.

Minor alterations were detected in the variations of individual amino acid’s RMSf. In the context of AKT_1_ (Figure 6), it is seen that the linking-loop connecting the PH domain to the kinase domain displays significant oscillations, with a maximum amplitude of 25 Å. This suggests that the linking-loop has the capacity to assume many conformations, including open, closed, or intermediate states (Movie S1, see page S60 on Supplementary Material) during the interaction with AKT_1_-aIs, which may have the capacity to modulate cell signaling downstream. Regarding Ox_2_R, the RMSf demonstrates the stability of the six transmembrane helices and the occurrence of high-rate oscillations in the interconnecting loops (Movie S2). Nevertheless, the analysis of TM5 reveals slight fluctuation variations in two replicas during the interaction of Ox_2_R with **1f**. The limited extent of variations seen in the remaining systems implies a state of inactivity that maintains stability throughout oscillations in MD. Nonetheless, it is worth noting that all three systems display alterations in the structure of TM5, TM6, TM7, and H8 (Figure S26). Moreover, the results demonstrate that compounds **1f**, **1h**, and **1k** exhibit an influence on the conformational alterations occurring in the sidechains of the active pockets, interdomain linking loops in AKT_1_, and the transmembrane domains (TMs) in Ox_2_R. These findings may offer a possible rationale for the observed lowered IC_50_ values.

Regarding the acquired data, R_gyr_ is a metric utilized to assess the protein’s dynamic behavior, specifically its changes in structural compactness, which can be interpreted as expansions or contractions in its total globular size. Figure 6 and Figure 7 illustrate the varying R_gyr_ values observed for the AKT_1_ and Ox_2_R structures, exhibiting oscillations within the range of 21.5 to 22.0 Å for both systems (Table S5).

The internal graphics (Figure 7) illustrate the protein–RMSD (α-carbon), ligand–RMSD, RMSf, and normalized distribution of R_gyr_ across a simulated time of 100 ns in each replicate of every system. The replicates are distinguished by their respective colors, with blue representing replicate 1, red representing replicate 2, and green representing replicate 3. To minimize the possibility of incorrect interpretation, the first graph for each descriptor is provided with axis labels. The central region of the diagram illustrates the Ox_2_R target protein’s structure in cartoon representations, whereby a **1k** ligand has been introduced into the active pocket.

Into two AKT_1_ replicas, there is an observed phenomenon of a widened distribution of the population bell curve or the presence of two distinct populations. This observation cannot be attributed to a denaturation state. One instance illustrating this behavior is replica 2 (Figure 6, red line) pertaining to ligand **1f**, wherein the observed variations arise from the movements of the linking-loop (Movie S1) between PH and kinase domains. The observed variations could potentially be attributed to the allosteric dynamics of inhibited AKT_1_. In the context of Ox_2_R, the population oscillations observed for R_gyr_ are shown to be constant and similar between replicates. Nevertheless, the systems undergo the most sudden alterations when Ox_2_R encounters ligand **1f**. Within this particular setting, it is possible that ligand **1f** elicits disturbances in the dynamic “breathing” fluctuations of Ox_2_R. These perturbations may align with its function as an inhibitor in the process of Ox_2_R inactivation, thereby suppressing its signaling functions.

#### 2.3.3. Binding Free Energy **1f**, **1h**, and **1k**

The MD simulations were employed to understand the binding interactions between the compounds (**1f**, **1h**, and **1k**) with target proteins (AKT_1_ and Ox_2_R) [47,48,49]. Hence, the computational method known as MM/GBSA was employed to calculate the interaction binding energy between compounds **1f**, **1h**, and **1k** and the proteins AKT_1_ and Ox_2_R. This method is recognized for its cost-effective nature [50,51,52]. The clustering analysis was performed on the three concatenated replicates across the final 80 ns of each simulation. Following the clustering approach, the cluster with the highest population, which accounted for at least 75% of the combined simulation dataset consisting of 1800 randomly selected structures, was further analyzed using the MM/GBSA method.

Based on the computed interaction energies, it can be inferred that the complexes involving putative compounds **1f**, **1h**, and **1k** exhibit stabilization mostly due to non-polar contributions (Table 2), when interacting with the protein therapeutic targets. In the context of AKT_1_, compound **1f** was observed to bind to the allosteric pocket with an interaction energy of −18.48 kcal/mol. Comparatively, the similarity compounds **1h** and **1f** exhibited interaction energies of −19.50 and −26.42 kcal/mol, respectively. Regarding the Ox_2_R inhibitor, it was observed that compound **1f** had a binding affinity of −14.26 kcal/mol towards the active site. Similarly, compounds **1h** and **1k** displayed binding affinities of −23.62 and 23.06 kcal/mol, respectively. The interaction energy of both systems was found to exhibit contributions from Van der Waals (vdW) forces ranging from 45 to 55 kcal/mol. In contrast, the electrostatic contributions varied from −5 to −25 kcal/mol. These results suggest that the vdW contributions were more energetically advantageous in comparison to the electrostatic contributions (Table 2).

The positive contribution of the polar interaction energy presents a drawback in the interaction process due to its positive contribution, which varies from 35 to 55 kcal/mol (Table 2). Within this framework, the non-polar nature of ligands **1f**, **1h**, and **1k** gives rise to the possibility of transitory electron shifts. These shifts lead to the formation of temporary dipoles and play a crucial role in determining the attractive forces between the side chains of the pocket targets and the inhibitors. The compounds under investigation possess a chemical composition characterized by a substantial electron count, rendering them very susceptible to polarization. This inherent property, in conjunction with hydrophobic interactions, provides a plausible explanation for the inhibitors’ affinity towards the binding sites associated with the AKT_1_ and Ox_2_R targets. Our findings demonstrate that ligands **1f** and **1k** prefer acting as allosteric inhibitors of AKT_1_. Additionally, ligand **1h** may be suggested as an inhibitor (inverse agonist) of Ox_2_R.

**Figure 8:**
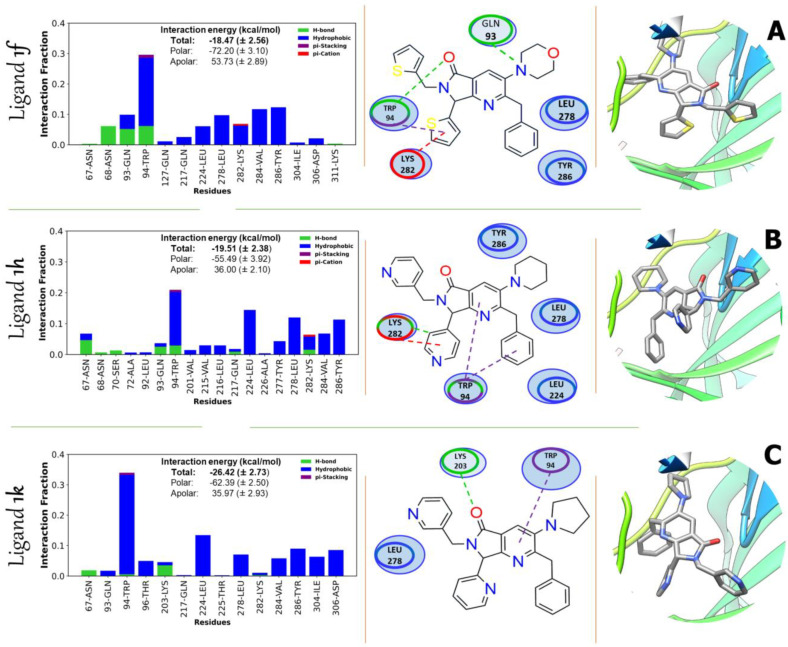
Main interactions between the protein target AKT_1_ and compounds **1f**, **1h**, and **1k**. These interactions are represented by the following arrangements: (**A**) **1f**, (**B**) **1h**, and (**C**) **1k.** Color coding: The interactions are visually depicted using circles containing residue names and lines indicating their spatial arrangement. The interaction colors are consistent across both the bar chart and the 2D maps. Green indicates hydrogen bonding interactions, while blue represents hydrophobic interactions. Purple is used for π-stacking interactions, and red is employed for π–cation interactions. The atom–ligand pairs are visually differentiated based on their respective colors. Carbon is represented by the color gray, nitrogen by blue, oxygen by red, and sulfur by yellow.

In addition, an analysis was conducted to decompose the energy of the interaction by residue (Table S6). This analysis aimed to discover the specific residues involved in the interaction process that contribute a significant energy value of more than 0.5 kcal/mol within a 5 Å radius from the surface of the ligand. In the case of AKT_1_, the residues GLN93, TRP94, and VAL284 made notable contributions (Figure 8). Specifically, for residue GLN93, the calculated energy values were −2.69 kcal/mol, −1.22 kcal/mol, and −1.12 kcal/mol for the compounds **1f**, **1h**, and **1k**, respectively. Similarly, for residue TRP94, the energy values were −3.64 kcal/mol, −2.60 kcal/mol, and −3.67 kcal/mol for the **1f**, **1h**, and **1k** inhibitors, respectively. Lastly, for residue VAL284, the energy values were −2.02 kcal/mol, −1.48 kcal/mol, and −2.11 kcal/mol for the **1f**, **1h**, and **1k** inhibitors, respectively. TRP94 present the most advantageous interaction energies among the three compounds (Figure 8). These include π-stacking-type interactions with the neighboring thiophene attached to the nitrogen atom of the pyrrolo[3,4-*b*]pyridin-5-one nucleus, hydrogen bonding with the oxygen of the pyrrolo[3,4-*b*]pyridin-5-one nucleus, and hydrophobic interactions for ligand **1f**.

A comparable phenomenon was noted while examining the behavior of ligand **1h** (Figure 8). However, in this case, the indole group of TRP94 exhibited a specific orientation that facilitated the formation of π-stacking and perpendicular contacts between the pyrrolo[3,4-*b*]pyridin-5-one nucleus and the benzene substituent. Moreover, it was observed that ligand **1k** exclusively formed π-stacking interactions with the pyrrolo[3,4-*b*]pyridin-5-one nucleus. Similarly, ligands **1f** and **1h** exhibited significant interactions with the amino acid residue LYS282, which subsequently displayed strong interactions with neighboring residues within the kinase domain, including VAL284, TYR286, and ARG287 (Figure 8). These four residues hold significant importance in the activation process of AKT_1_.

**Figure 9:**
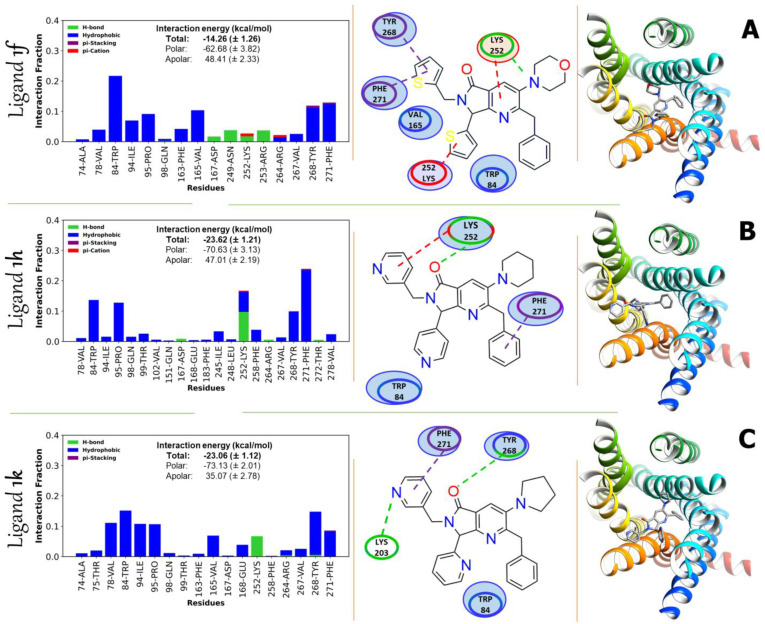
Main interactions that occur between the protein target Ox_2_R and compounds **1f**, **1h**, and **1k**. These interactions are represented by the following arrangements: (**A**) **1f**, (**B**) **1h**, and (**C**) **1k**. Color coding: The interactions are visually depicted using circles containing residue names and lines indicating their spatial arrangement. The interaction colors are consistent across both the bar chart and the 2D maps. Green indicates hydrogen bonding interactions, while blue represents hydrophobic interactions. Purple is used for π-stacking interactions, and red is employed for π–cation interactions. The atom–ligand pairs are visually differentiated based on their respective colors. Carbon is represented by the color gray, nitrogen by blue, oxygen by red, and sulfur by yellow.

The bar graphs depicted in Figure 8 and Figure 9 showcase the interactions governing the recognition process for AKT_1_ and Ox_2_R between compounds **1f**, **1h**, and **1k**, respectively. Additionally, both display the residence periods of these interactions, measured in snapshots, and rated by the Protein-Ligand Interaction Profiler (PLIP). The horizontal axis of the graphs reflects residue counts, while the vertical axis corresponds to population percentages. Each bar represents the average interaction type. The inset of the graphs illustrates the interaction energy (measured in kcal/mol) between the substrate and each possible inhibitor. Specifically, it focuses on residues located within a 5 Å radius of the binding allosteric site. Following this, a two-dimensional heatmap illustrates the interactions of AKT_1_ and Ox_2_R with compound interactions. The heatmap was generated using snapshots obtained from the representative structure of the most populated cluster, and the interactions were evaluated using Discovery Studio. The fingerprint map illustrates the predominant interactions between AKT_1_ residues and the compounds, with a particular focus on the preservation of TRP94 and LYS282 π-stacking, hydrophobic, and π–cation interactions. Similarly, it highlights the interactions between Ox_2_R residues and compounds, with a particular focus on preserving LYS2526.58, TYR2687.31, and PHE2717.34 π-stacking, hydrophobic, and π–cation contacts throughout the 100 ns MD production simulations for compounds **1f**, **1h**, and **1k**. At some point, a 3D inhibitor spatial conformation is presented for each complex. Figure 8 and Figure 9 illustrate the spatial arrangement of the compounds within the allosteric pocket of AKT_1_ and the active pocket of Ox_2_R.

The activation of the ATP binding site is conditioned upon conformational changes in the AKT_1_ activation loop. This modification occurs exclusively when the phosphorylated activation loop interacts with specific residues (LYS282, VAL294, TYR286, or ARG287) situated within the kinase domain. In light of this, we have put forth a hypothesis suggesting that the binding of compounds **1f** and **1h** to the allosteric site could potentially impede ATP binding. The interaction between residues LYS282, VAL294, and TYR286 within the activation loop is hindered by the presence of **1f** and **1h**. Hence, the strategic placement of compounds **1f** and **1h**, which exhibit advantageous energy values as indicated in Table 2, at the allosteric site will impede the dephosphorylation process of the activation loop. As a result, inactivation of AKT_1_ downstream signaling would show reduced efficacy. Compound **1k**, which demonstrated the most substantial interaction energy of −26.42 kcal/mol with AKT_1_, exhibited comparable interaction patterns to inhibitors **1f** and **1h**. However, it also displayed a broader energy distribution, engaging a larger number of residues (Figure 8. This collective effect resulted in a favorable increase in interaction energy, thereby promoting inhibition. In addition, the hydrophobic residues, including LEU278 and THR305, play a significant role in the hydrophobic interaction energy within the allosteric site of AKT_1_. The simulation results demonstrate that ligands **1f**, **1h**, and **1k** (Figure 8) exhibit several conformations, indicating their potential interactions with residues such as ASN67, ASN68, GLN93, and TRP94 in the PH motifs, as well as GLN217, LEU224, and THR225 in the *N*-Lobe, and LEU278, LYS282, VAL 284, and TYR286 in the *C*-Lobe.

The interactions in Ox_2_R did not correspond with the results presented by Nagahara et al. in 2015 regarding the regulation of Ox_2_R via specific residues Thr111^2.60^, Asp115^2.64^, His350^7.38^, and Tyr354^7.42^ (referred to as the agonistic tetrad) [53]. Nevertheless, a previous investigation conducted by Heifetz et al. in 2013 underscored the significance of TYR268^7.31^ (TYR317^7.38^ in its native form as Ox_2_R) in facilitating the activation of OX_2_R by orexin peptide A [54]. In this context, it is observed that compounds obstructed the TYR268^7.31^ interactions; Figure 9. Compound **1f** predominantly engages with the side chains of the amino acids TRP84^1.58^, LYS252^6.58^, TYR268^7.31^, and PHE271^7.34^ (Figure 9). These interactions are characterized by respective binding energy contributions of −1.23 kcal/mol, −0.52 kcal/mol, −1.79 kcal/mol, and −1.17 kcal/mol (Table S6). Compound **1h** exhibits a prominent interaction with the side chains of amino acids LYS252^6.58^, TYR268^7.31^, and PHE271^7.34^, resulting in binding energy contributions of −0.91 kcal/mol, −1.65 kcal/mol, and −2.44 kcal/mol, respectively. **1k** predominantly forms associations with the side chains of amino acids TYR268^7.31^ and PHE271^7.34^, resulting in binding energy contributions of −1.57 kcal/mol and −1.63 kcal/mol, respectively.

The energy increase experienced by each residue is primarily determined by the specific contact exerted by each ligand (Figure 9). Regarding this matter, residue PHE271^7.34^ facilitates a hydrophobic contact and a π-stacking interaction with ligand **1f**. The π-stacking interaction is observed between TYR268^7.31^ and the thiophene group attached to the nitrogen atom of the pyrrolo[3,4-*b*]pyridin-5-one nucleus. The consistency of this interaction is observed across all inhibitors, with the sole difference being the way in which residue PHE271^7.34^ is accommodated on the pyrrolo[3,4-*b*]pyridin-5-one nucleus. This adjustment is contingent upon the orientation of the inhibitors, as illustrated in Figure 9. In the instance of LYS252^6.58^, it mostly engages in hydrogen bonding and π–cation interactions, either with the pyrrolo[3,4-*b*]pyridin-5-one core of **1f** or the amino pyridine substituent of **1h**. In the present context, the observed variability in energy contributions pertaining to ligand **1h** may provide insights into the factors underlying the more favorable interaction energy between inhibitor **1h** and Ox_2_R, as opposed to AKT_1_.

Given the significant involvement of the amino acid TYR268^7.31^ in the activation mechanisms of Ox_2_R, as well as its apparent inhibition by ligands **1f**, **1h**, and **1k** through favorable interactions, it can plausibly be suggested that these ligands have the potential to interfere with the interactions between transmembrane helices 5 (TM5) and 6 (TM6). The potential intermembrane contact described may give rise to an inhibitor outcome due to the interconnected inward motion of TM5 and TM6 helices, which is also associated with the activation of Ox*_2_*R [50,51,52,53,54,55,56,57]. Therefore, it can be postulated that the three inhibitors could disrupt the connections between transmembrane helices. In a manner analogous to the simulations performed for compounds **1f**, **1h**, and **1k** with AKT_1_, the simulations undertaken to explore the inhibition of Ox_2_R reveal that compounds **1f**, **1h**, and **1k** assume several conformations and display noteworthy interactions with TM6 and TM7 (Figure S26).

## 3. Materials and Methods

### 3.1. Synthesis

#### 3.1.1. General Information, Instrumentation, Software, and Chemicals

^1^H and ^13^C nuclear magnetic resonance (NMR) spectra were acquired on a Bruker AMX Advance III spectrometer (500 MHz, Fällande, Uster, Switzerland). The solvent used for NMR experiments was deuterated chloroform (CDCl_3_). Chemical shifts are reported in parts per million (δ/ppm). Coupling constants are reported in Hertz (*J*/Hz). Internal reference for NMR spectra was tetramethylsilane (TMS) at 0.00 ppm. Multiplicities of the signals are reported using the standard abbreviations: singlet (s), doublet (d), triplet (t), quartet (q), and multiplet (m). NMR spectra were analyzed using the MestReNova software (Ver. 12.0.0-20080). Infrared (IR) spectra were acquired on a Perkin Elmer 1600 spectrometer (Norwalk, CT, USA) using the attenuated total reflectance (ATR) method. The maximum absorbance peaks are reported in reciprocal centimeters (ν_max_/cm^−1^). IR spectra were analyzed using the Report Builder software (Ver. 2.01). High-resolution mass spectroscopy (HRMS) spectra were acquired by electrospray ionization (ESI) on a Micro-TOF II spectrometer Bruker Daltonics GmbH (Bremen, Germany). HRMS samples were injected directly (Apollo source) and analyzed by time-of-flight method (TOF). HRMS spectra were analyzed using the Compass analysis software (Ver. 1.5, Flex Control and Flex Analysis by Bruker Daltonics, Inc.). Microwave-assisted reactions were performed in closed-vessel mode on a CEM Discover SP MW-reactor (Matthews, North Carolina, CA, USA). Reaction progress was monitored by thin-layer chromatography (TLC) and the spots were visualized under ultraviolet (UV) light (254 or 365 nm). Flash columns packed with oxide aluminum in a 0.063–0.200 mm mesh particle size were used to purify the products. Mixtures of hexanes (Hex) and ethyl acetate (EtOAc) in 1:1 or 1:2 (*v/v*) proportion were used to run TLC, aluminum oxide columns, and to measure the retention factor (R*_f_*) values (using the same mobile phase for all the experiments). All starting reagents and solvents were used as received (without further purification, distillation, or dehydration). Chemical structures were drawn using the ChemDraw Professional software (Ver. 15.0.0.106, Perkin Elmer Informatics, Cambridge, MA, USA). The purity for all synthesized products (>95%) was assessed by NMR.

#### 3.1.2. Synthesis and Characterization of the Pyrrolo[3,4-b]pyridin-5-ones **1g**–**1k**

General procedure (GP): The corresponding aldehydes **2** (1.0 equiv.) and the amines **3** (0.1 mmol, 1.0 equiv.) were placed in a sealed CEM Discover microwave reaction tube (10 mL) and diluted in toluene (1.0 mL). Then, the mixture was stirred and heated using microwave irradiation (65 °C, 100 W) for 5 min, and ytterbium(III) triflate (0.03 equiv.) was added. The mixture was stirred and heated using microwave irradiation (65 °C, 100 W) for 5 min, and then the corresponding isocyanides **5** (1.2 equiv.) were added. The new mixture was stirred and again heated using microwave irradiation (70 °C, 150 W) for 15 min, and then maleic anhydride (**7**) (1.4 equiv.) was added. Finally, the reaction mixture was stirred and heated using microwave irradiation (80 °C, 150 W) for 15 min. Then, the solvent was removed to dryness under vacuum. The crude was extracted using dichloromethane (3 × 25.0 mL) and Na_2_CO_3 (aq.)_ (3 × 25 mL), and then washed with brine (3 × 25 mL). The organic layer was dried using anhydrous Na_2_SO_4_, filtered, and concentrated to dryness under vacuum. The new crude was purified by aluminum oxide column chromatography using mixtures of hexanes (Hex) and ethyl acetate (EtOAc) in 1:1 (*v*/*v*) proportions as a mobile phase to isolate the corresponding pyrrolo[3,4-*b*]pyridin-5-ones **1g**–**1k**.

2-benzyl-3-(diethylamino)-7-(pyridin-3-yl)-6-(pyridin-3-ylmethyl)-6,7-dihydro-5*H*-pyrrolo[3,4-*b*]pyridin-5-one (**1g**)

According to the GP, 3-Pyridinecarboxaldehyde (100.0 μL), 3-Picolylamine (107.0 μL), ytterbium(III) triflate (19.0 mg), *N*,*N*-diethyl-2-isocyano-3-phenylpropanamide (289.0 mg), and maleic anhydride (143.0 mg) were reacted together in toluene (1.0 mL) to afford **1g** (27.0 mg, 22%) as a yellow oil; R*_f_* = (Hex–AcOEt = 1:1, *v*/*v*); FT-IR (ATR) ν_max_/cm^−1^ 1693 (C = O); ^1^H NMR (500 MHz, CDCl_3_): δ 8.65 (dd, *J* = 4.8, 1.7 Hz, 1H), 8.56 (dd, *J* = 4.8, 1.7 Hz, 1H), 8.51 (d, *J* = 2.4 Hz, 1H), 8.47 (d, *J* = 2.4 Hz, 1H), 7.92 (s, 1H), 7.62–7.59 (m, 1H), 7.41–7.37 (m, 1H), 7.33–7.30 (m, 1H), 7.29–7.26 (m, 1H), 7.19–7.13 (m, 5H), 5.38 (d, *J* = 15.3 Hz, 1H), 5.31 (s, 1H), 4.32 (d, *J* = 14.0 Hz, 1H), 4.17 (d, *J* = 14.0 Hz, 1H), 4.00 (d, *J* = 15.2 Hz, 1H), 3.03 (q, *J* = 7.1 Hz, 4H), 0.97 (t, *J* = 7.1 Hz, 6H) ppm; ^13^C NMR (125 MHz, CDCl_3_): δ 167.4, 164.1, 158.7, 150.2, 149.6 (2), 149.3, 146.9, 139.2, 136.1, 135.3, 132.2, 131.2, 128.9 (2), 127.9 (2), 125.9, 125.7, 123.8, 123.7, 122.8, 62.3, 47.6 (2), 41.7, 39.8, 12.0 (2) ppm; HRMS (ESI^+^): *m/z* calcd for C_29_H_29_N_5_O [M + H]^+^ 464.2450, found 464.2445.

2-benzyl-3-(piperidin-1-yl)-6-(pyridin-3-ylmethyl)-7-(pyridin-4-yl)-6,7-dihydro-5*H*-pyrrolo[3,4-*b*]pyridin-5-one (**1h**)

According to the GP, 4-Pyridinecarboxaldehyde (100.0 μL), 3-Picolylamine (106.0 μL), ytterbium(III) triflate (19.0 mg), 2-isocyano-3-phenyl-1-(piperidin-1-yl)propan-1-one (300.0 mg), and maleic anhydride (141.0 mg) were reacted together in toluene (1.0 mL) to afford **1h** (25.0 mg, 20%) as a yellow oil; R*_f_* = (Hex–AcOEt = 1:1, *v*/*v*); FT-IR (ATR) ν_max_/cm^−1^ 1694 (C = O); ^1^H NMR (500 MHz, CDCl_3_): δ 8.62–8.60 (m, 2H), 8.53 (dd, *J* = 4.8, 1.7 Hz, 1H), 8.42–8.40 (m, 1H), 7.84 (s, 1H), 7.56–7.53 (m, 1H), 7.23 (ddd, *J* = 7.8, 4.8, 0.9 Hz, 1H), 7.17–7.12 (m, 5H), 7.10–7.08 (m, 2H), 5.40 (d, *J* = 15.2 Hz, 1H), 5.18 (s, 1H), 4.23 (d, *J* = 13.9 Hz, 1H), 4.12 (d, *J* = 13.9 Hz, 1H), 3.91 (d, *J* = 15.2 Hz, 1H), 2.82–2.78 (m, 4H), 1.75–1.69 (m, 4H), 1.62–1.57 (m, 2H) ppm; ^13^C NMR (125 MHz, CDCl_3_): δ 167.6, 162.7, 157.9, 150.5 (2), 149.8, 149.6, 149.4, 144.6, 139.1, 136.2, 132.1, 128.8 (2), 128.1 (2), 126.1, 123.8, 123.3, 122.9, 122.6 (2), 63.3, 54.2 (2), 41.8, 39.7, 26.3 (2), 23.9 ppm; HRMS (ESI^+^): *m/z* calcd for C_30_H_30_N_5_O [M + H]^+^ 476.2437, found 476.2445.

2-benzyl-3-(piperidin-1-yl)-7-(pyridin-2-yl)-6-(pyridin-3-ylmethyl)-6,7-dihydro-5*H*-pyrrolo[3,4-*b*]pyridin-5-one (**1i**)

According to the GP, 2-Pyridinecarboxaldehyde (100 μL), 3-Picolylamine (108 μL), ytterbium(III) triflate (19 mg), 2-isocyano-3-phenyl-1-(piperidin-1-yl)propan-1-one (306 mg), and maleic anhydride (144 mg) were reacted together in toluene (1.0 mL) to afford **1i** (115 mg, 92%) as a yellow oil; R*_f_* = (Hex–AcOEt = 1:1, *v*/*v*); FT-IR (ATR) ν_max_/cm^−1^ 1691 (C = O); ^1^H NMR (500 MHz, CDCl_3_): δ 8.58 (ddd, *J* = 4.9, 1.8, 0.9 Hz, 1H), 8.46 (dd, *J* = 4.8, 1.7 Hz, 1H), 8.41–8.39 (m, 1H), 7.87 (s, 1H), 7.61–7.57 (m, 2H), 7.24–7.19 (m, 2H), 7.17–7.06 (m, 3H), 7.05–7.01 (m, 1H), 5.46 (s, 1H), 5.20 (d, *J* = 15.1 Hz, 1H), 4.22 (d, *J* = 13.6 Hz, 1H), 4.20–4.16 (m, 2H), 2.81–2.75 (m, 4H), 1.74–1.65 (m, 4H), 1.61–1.53 (m, 2H) ppm; ^13^C NMR (125 MHz, CDCl_3_): δ 167.5, 162.2, 158.3, 155.4, 149.9, 149.8, 149.6, 148.9, 139.4, 136.9, 136.3, 132.7, 128.8 (2), 128.0 (2), 125.9, 123.8, 123.5, 123.4, 123.4, 123.0, 66.2, 54.2 (2), 42.1, 39.8, 26.3 (2), 23.9 ppm; HRMS (ESI^+^): *m/z* calcd for C_30_H_30_N_5_O [M + H]^+^ 476.2442, found 476.2445.

2-benzyl-3-(diethylamino)-7-(pyridin-2-yl)-6-(pyridin-3-ylmethyl)-6,7-dihydro-5*H*-pyrrolo[3,4-*b*]pyridin-5-one (**1j**)

According to the GP, 2-Pyridinecarboxaldehyde (100 μL), 3-Picolylamine (108 μL), ytterbium(III) triflate (19 mg), *N*,*N*-diethyl-2-isocyano-3-phenylpropanamide (291 mg), and maleic anhydride (144 mg) were reacted together in toluene (1.0 mL) to afford **1j** (90 mg, 74%) as a yellow oil; R*_f_* = (Hex–AcOEt = 1:1, *v*/*v*); FT-IR (ATR) ν_max_/cm^−1^ 1692 (C = O); ^1^H NMR (500 MHz, CDCl_3_): δ 8.60 (ddd, *J* = 4.9, 1.8, 0.9 Hz, 1H), 8.47 (dd, *J* = 4.8, 1.7 Hz, 1H), 8.41 (dd, *J* = 2.3, 0.8 Hz, 1H), 7.88 (s, 1H), 7.61 (dddd, *J* = 7.7, 5.1, 2.7, 1.8 Hz, 2H), 7.23 (ddd, *J* = 7.6, 4.9, 1.2 Hz, 1H), 7.19 (ddd, *J* = 7.9, 4.8, 0.8 Hz, 1H), 7.14–7.11 (m, 5H), 7.03 (d, *J* = 7.8, 1.0 Hz, 1H), 5.47 (s, 1H), 5.22 (d, *J* = 15.1 Hz, 1H), 4.25 (d, *J* = 13.9 Hz, 1H), 4.23–4.17 (m, 2H), 2.97 (q, *J* = 7.1 Hz, 4H), 0.92 (t, *J* = 7.1 Hz, 6H) ppm; ^13^C NMR (125 MHz, CDCl_3_): δ 167.5, 163.6, 158.4, 155.3, 149.9, 149.8, 148.9, 146.7, 139.4, 136.8, 136.3, 132.6, 129.5, 128.9 (2), 127.8 (2), 125.8, 123.5, 123.4, 122.9, 66.2, 47.7 (2), 42.1, 39.8, 12.0 (2) ppm; HRMS (ESI^+^): *m/z* calcd for C_29_H_30_N_5_O [M + H]^+^ 464.2445, found 464.2545.

2-benzyl-7-(pyridin-2-yl)-6-(pyridin-3-ylmethyl)-3-(pyrrolidin-1-yl)-6,7-dihydro-5*H*-pyrrolo[3,4-*b*]pyridin-5-one (**1k**)

According to the GP, 2-Pyridinecarboxaldehyde (100 μL), 3-Picolylamine (108 μL), ytterbium(III) triflate (19 mg), 2-isocyano-3-phenyl-1-(pyrrolidin-1-yl)propan-1-one (288 mg), and maleic anhydride (144 mg) were reacted together in toluene (1.0 mL) to afford **1k** (110 mg, 91%) as a yellow oil; R*_f_* = (Hex–AcOEt = 1:1, *v*/*v*); FT-IR (ATR) ν_max_/cm^−1^ 1693 (C = O); ^1^H NMR (500 MHz, CDCl_3_): δ 8.57 (ddd, *J* = 4.9, 1.8, 0.9 Hz, 1H), 8.45 (dd, *J* = 4.8, 1.7 Hz, 1H), 8.41 (dd, *J* = 2.3, 0.8 Hz, 1H), 7.67 (s, 1H), 7.58 (dd, *J* = 7.6, 1.8 Hz, 2H), 7.23–7.09 (m, 5H), 7.05–7.02 (m, 2H), 7.01 (d, *J* = 7.8 Hz, 1H), 5.44 (s, 1H), 5.19 (d, *J* = 15.1 Hz, 1H), 4.24 (d, *J* = 5.5 Hz, 2H), 4.18 (d, *J* = 15.2 Hz, 1H), 3.20–3.09 (m, 4H), 1.92–1.82 (m, 4H) ppm; ^13^C NMR (125 MHz, CDCl_3_): δ 167.7, 156.4, 155.6, 155.1, 149.7, 149.6, 148.8, 146.1, 139.4, 136.7, 136.1, 132.6, 128.3 (2), 127.9 (2), 125.7, 123.5, 123.3, 123.1, 122.8, 118.5, 66.0, 51.8 (2), 42.0, 41.5, 25.0 (2) ppm; HRMS (ESI^+^): *m/z* calcd for C_29_H_28_N_5_O [M + H]^+^ 462.2298, found 462.2288

### 3.2. Anticancer Activity

#### 3.2.1. Reagents

Culture Dulbecco’s Modified Eagle Medium (DMEM) was purchased from Sigma-Aldrich (St Louis, MO, USA), antibiotics/antimycotics GIBCO^®^ (5240-062). MTT (M2128) was purchased from Sigma-Aldrich (St Louis, MO, USA).

#### 3.2.2. Cell Culture

The MCF-7 and MDA-MB-231 breast cancer cells lines were obtained from ATCC (Manassas, VA), and were cultured in DMEM/F12 medium (50:50, V:V; Sigma-Aldrich, St Louis, MO, USA) with 5% fetal bovine serum (FBS) and 1% antibiotic (penicillin G/streptomycin, Gibco, Waltham, MA, USA). Cells were maintained in a humidified atmosphere with 5% CO_2_ at 37 °C. The MCF-7 and MDA-MB-231 breast cancer cells were serum-deprived for 24 h before treatment.

#### 3.2.3. Cell Viability Assays

Viability was measured by MTT (3-[4,5-dimethylthiazol-2-yl]-2,5 diphenyl tetrazolium bromide) assay. A quantity of 2 × 10^4^ cells were seeded and grown overnight in a 96-well plate. Treatments with the synthesized compounds were performed by employing different concentrations ranging from 6.25 to 200 mM. After 24 h and 48 h of treatment, 0.5 mg/mL MTT was added to each well and incubated at 37 °C for 4 h, protected from light. Formazan crystals were dissolved in DMSO, and absorbances were measured at 570 nm in a MultiskanTM GO microplate spectrophotometer (ThermoScientific, Waltham, MA, USA). The percentage of cell viability was calculated as the ratio of optical densities of the control and experimental groups.

#### 3.2.4. Statistical Analysis

The results are presented as the mean ± SD. Data were analyzed statistically by one-way ANOVA, and comparisons were performed by Dunnett’s multiple comparison test. A statistical probability of *p* < 0.05 was considered significant.

### 3.3. In Silico Studies

#### 3.3.1. ADME and Tox Properties

The assessment of Absorption, Distribution, Metabolism, Excretion, and Toxicity (ADMETox) characteristics was carried out. The assessment of ADMETox characteristics was conducted using the SwissADME (Ver. 2017) [58] and ProTox-II (Ver. 2021) [59] tools. The predictions were conducted using support vector machine (SVM) and machine learning techniques, respectively. Multiple criteria were evaluated, including gastrointestinal absorption, permeability across the blood–brain barrier, potential as a substrate or inhibitor of P-glycoprotein, inhibition of the cytochrome family, and suitability for artificial synthesis.

#### 3.3.2. Target Selection and Active Pocket Determination

The process of selecting targets for ligands **1a**–**1k** was conducted via SwissTargetPrediction (Ver. 2019) [60] and PASS-Protein-Target (Ver. 2.0) [61], which are computational systems that use a machine learning approach. The therapeutic targets that were selected for further investigation were those that had the greatest likelihood and significance in relation to breast cancer. A total of 20 proteins (Table S2) with known structures in the Protein Data Bank (PDB) https://www.rcsb.org/ (accessed on 8 February 2023) [62] were chosen for the virtual screening procedure. The active pocket was identified using the Ligand Designer program [63], available on the publicly accessible CHARM-GUI [64]. The G-LoSA method was used to identify the appropriate active site, as indicated by a GA-score greater than 0.9 (Table S2).

#### 3.3.3. Ligand Optimization

The ligands **1a**–**1k** underwent a geometric complete geometry optimization, accompanied by a frequency calculation in Gaussian 09 (Table S7). This optimization was conducted without any limitations to get the structures that had the lowest energy [36,65]. The optimization procedure used the B3LYP/6-31+g(d) basis set and approach, adding a solvent model based on the density of charge (SMD). The optimum shape was achieved in conditions of high moisture. After the production of output xyz files, the ligands were subjected to processing using Discovery Studio [66]. The first stage of this process was the conversion of the ligand files into SDF format, followed by the addition of polar hydrogens. Following that, the ligands underwent processing using the Meeko program (available at https://github.com/forlilab/Meeko) (accessed on 10 August 2023) to produce pdbqt files. These files were then combined using the ADFR software package (Ver. 1.0) [67].

#### 3.3.4. Homology Modeling and Docking Simulations

The process of modeling missing segments in the crystallographic proteins was carried out using SwissModel (Table S8) [68]. The crystallographic structures of the proteins were used as templates, and the modeling was performed by considering the GMQE and QMEANDisCo values, both of which were required to be greater than 0.9. The assessment of model integrity was conducted using Molprobity [69] and VERIFY3D [70]. The elucidated proteins were used as targets for molecular docking [42]. The non-protein components that were found in the crystallographic structures were eliminated from the receptor. Subsequently, the structure was subjected to minimization using the YASARA server [71] in an aqueous environment with a solvent shell of 6 Å. Additionally, the pKa values of the amino acids were estimated at a pH of 7.4. The minimization process was terminated when the energy exhibited an improvement of less than 0.0119 kcal/mol (0.05 kJ/mol) and 200 steps in the steepest descent method.

Autodock Vina version 1.2.5 [72] was used for the purpose of molecular docking. Distinct grid centers and sizes were established for each target protein (Table S2). The docking simulations were conducted autonomously with three distinct force fields: AD4, Vina, and Vinardo. A total of 13,860 independent docking experiments were conducted, where each of the 11 ligands was docked 20 times to each of the 20-target proteins, using the three different force fields. To assess the reliability of the docking outcomes and the prediction of the active site, a crystallographic ligand was submitted to blind docking over two proteins of twenty target proteins. The calculated RMSD values were found to be 3.18 Å for the water-soluble protein MAPK8 (PDB-ID: 5IU2) and 5.57 Å for the membrane protein ghrelinR (PDB-ID: 7NA8). The superposition of the blind docking is depicted in Figure S27 [73,74,75,76].

To improve the accuracy of the docking analysis, the ligands were subjected to a redocking procedure and subsequently classified using a clustering, with a cutoff of 0.5 Å. The ligands selected for subsequent MD analyses were those with binding scores that were one standard deviation below the mean and belonged to the most densely populated clusters [42].

#### 3.3.5. Molecular Dynamics Simulations

The molecular complexes that displayed the most favorable energy scores and exhibited the lowest IC_50_ values were chosen for subsequent MD simulations using the GROMACS program [77]. The protonation states of each protein were modified to conform to the physiological pH using the propKa tool [78] available on the PDB2PQR website (https://server.poissonboltzmann.org/) (accessed on 14 August 2023). The construction of input files was carried out with the Input Generator CHARMM-GUI [79]. The CHARMM36m force field [80] was used for the simulations. The Ligand Reader and Modeler CHARMM-GUI suite [81] was used in conjunction with CGenFF to get the ligand parameters.

The orthorhombic cell, with a thickness of 15 Å, was used to enclose each system. TIP3P water molecules were employed to surround the cell and, to neutralize the system’s charge, 0.15 M NaCl was introduced. A total of eighteen systems were constructed, with nine of them using AKT kinases (AKT_1_), while the other nine systems included the Orexin-2 receptor (Ox_2_R). The systems underwent a sequential process, which included an initial minimization phase consisting of 10,000 iterations using the steepest descent method. This was followed by two subsequent phases of MD simulations, with the first phase lasting 10 ns and conducted in the NVT ensemble, and the second phase lasting 20 ns and conducted in the NPT ensemble. These MD simulations were performed to achieve system equilibration. Following that, MD simulations lasting 100 ns were conducted. In the case of AKT_1_ systems, a timestep of 4 femtoseconds was used together with Hydrogen-Mass-Repartitioning (HMR) [82]. Conversely, for Ox_2_R, a timestep of 2 femtoseconds was utilized. The use of periodic boundary conditions (PBC) and Particle-Mesh-Ewald (PME) was employed for the treatment of electrostatic interactions. Inside the NPT ensemble, the temperature coupling was performed using the velocity rescale method, and the pressure coupling was performed using the Parrinello–Rahman method. The system was maintained at a temperature of 310.15 K and a pressure of 1 bar.

The simulation of Ox_2_R, a membrane protein, using the parameters established by Marrink et al. (2014) [83] and Im et al. (2022) [84], specifically designed for a human plasma membrane. The lipid content of the inner leaflet was found to be composed of 3-palmitoyl-2-oleoyl-d-glycero-1-phosphatidylcholine (POPC), 1-palmitoyl-2-linoleoyl-phosphatidylcholine (PLPC), 1-palmitoyl-2-arachidonyl-phosphatidylethanolamine (PAPE), 3-palmitoyl-2-oleoyl-d-glycero-1-phosphatidylethanolamine (POPE), sphingomyelin (SSM), n-stearoyl-sphingomyelin (NSM), and cholesterol (CHL1) in a ratio of 16:22:3:3:11:11:4:37, respectively. On the other hand, the outer leaflet exhibited a lipid composition consisting of POPC, PLPC, PAPE, POPE, phosphatidylinositol (POPI), 1-palmitoyl-2-arachidonyl-phosphatidylserine (PAPS), 3-palmitoyl-2-oleoyl-d-glycero-1-phosphatidic acid (POPA), SSM, NSM, and CHL1 in a ratio of 7:11:12:14:5:11:1:5:5:29. The construction of the asymmetric mammalian plasma membrane with 203 was carried out with the Membrane Builder suite [85] inside the CHARMM-GUI.

The MD simulations were carried out in triplicate, with each run being independent. The simulations encompassed three distinct ligands (**1f**, **1h**, and **1k**) and two target proteins (AKT_1_ and Ox_2_R), yielding a total of six systems, each with three replicas. The total time of the overall simulation effort was 1.8 µs. The trajectory analysis was conducted using the MDAnalysis software (Ver. 2.6.1) [86], and the pictures were displayed using Chimera [87].

#### 3.3.6. Binding Free Energy

The molecular mechanics generalized Born surface area (MM/GBSA) approximation was used to obtain the interaction energy [88,89,90]. The gmx_MMPBSA program, which is derived from AMBER’s MMPBSA.py [91,92], was used to calculate the interactions energies of MM/GBSA. The GB approximation is a computational method that efficiently incorporates solvation effects into molecular interactions. A clustering study was conducted by concatenating the three replicas for each system, with the objective of calculating the interaction energy. The clustering analysis was conducted on the equilibrated segment of the simulation, which spanned 240 ns and consisted of 2400 frames. The clustering analysis was conducted utilizing the GROMACS cluster program, with a cutoff value of 2.0 Å [93,94], specifically targeting α-carbons to guarantee the inclusion of a minimum of 75% of the structures in the first cluster.

The binding free energy (*Δ*G binding) was determined according to the equation:

(1) $$\Delta G_{binding}=G_{complex}^{}-\left(G_{protein}+G_{ligand}\right)$$

Calculation of free energy value G:

(2) $$G=E_{MM}+G_{solv}-TS_{MM}$$

where:

- $\Delta G_{binding}$: represents the binding free energy.
- $G_{complex}^{}$: is the energy of the complex.
- $G_{protein}$: in complex is the energy of the protein in the complex.
- $G_{ligand}$: in complex is the energy of the ligand in the complex.
- 〈G〉 denotes the calculated free energy value.
- $E_{MM}$ stands for the molecular mechanics energy.
- $G_{solv}$ is the solvation energy.
- T represents the temperature.
- $S_{MM}$ refers to the average molecular mechanics entropy.

Since entropy was not calculated during the experiment, the reported binding free energies only show the enthalpic part of the binding free energy [42]. To examine the protein–ligand interactions, we used the PLIP version 2.2.2 [95,96]. The Ballesteros numbering scheme, developed by Ox_2_R, was implemented using the online platform available at https://gproteindb.org/residue/residuetable (accessed on 8 February 2023) [97].

## 4. Conclusions

The investigation of molecules potentially possessing pharmacological properties that may effectively engage multiple protein receptors offers a promising strategy for addressing sophisticated medical problems, such as breast cancer. The pharmaceutical compounds synthesized for the present study exhibited interesting properties and numerous benefits, including the abilities to enhance therapeutic efficacy through synergistic interactions with various targets, enhance safety profiles, and decrease required dosages. Then, computer simulations were performed to enhance the progress of multi-target molecules for the treatment of cancer and accelerate the identification and progression of innovative therapeutic treatments.

Interestingly, of all compounds evaluated here, compound **1f** showed the greatest potential as a viable and promising candidate for cancer treatment in triple-negative breast cancer, exhibiting the lowest IC_50_ at 14.8 mM and morphological changes, indicative of decreased cell viability in the triple-negative breast cancer cell line MDA-MB-231. Nevertheless, there is a dearth of comprehension around the precise pathways through which **1f** exhibits its antineoplastic properties, underscoring the imperative for additional investigation in the realm of cancer treatment.

## Figures and Tables

**Table 1:** IC_50_ of the different compounds in MCF-7 and MDA-MB-231 breast cancer cells.

Compound	MCF-7 IC_50_ (µM)	MDA-MB-231 IC_50_ (µM)
**1a**	−2432.33696	114.998827
**1b**	277.07712	89.7144132
**1c**	554.323499	117.833333
**1d**	331.476877	**67.8459821**
**1e**	753.2345013	77.5989269
**1f**	753.234501	**14.8518557**
**1g**	158.183608	68.6064836
**1h**	109.624029	75.5411255
**1i**	123.0212854	**59.4034271**
**1j**	159.288991	96.820554
**1k**	187.18081	117.33474

**Table 2:** Binding interaction energies for potential inhibitors targeting AKT_1_ and Ox_2_R.

		***Contributions of Binding Interaction Energy (kcal/mol)***						
	**Inhibitor**	**E_vdW_**	**E_ELE_**	**E_GB_**	**E_SURF_**	**G_Apolar_**	**G_polar_**	**Total**
**AKT_1_**	**1f**	−51.85 (±1.92)	−20.35 (±1.96)	60.17 (±3.17)	−6.44 (±0.46)	−72.20 (±3.04)	53.73 (±2.89)	−18.48 (±1.56)
	**1h**	−45.38 (±1.83)	−10.11 (±1.32)	42.04 (±2.37)	−6.05 (±0.50)	−55.49 (±3.92)	36.00 (±3.10)	−19.5 (±0.38)
	**1k**	−54.5 (±2.31)	−7.88 (±0.66)	42.70 (±2.30)	−6.74 (±0.50)	−62.39 (±2.50)	35.97 (±3.93)	−26.42 (±0.73)
**Ox_2_R**	**1f**	−46.71 (±1.99)	−15.97 (±1.3)	54.37 (±2.36)	−5.95 (±0.40)	−62.68 (±3.82)	48.41 (±4.33)	−14.26 (±0.26)
	**1h**	−44.55 (±2.09)	−26.08 (±1.77)	53.22 (±3.84)	−6.21 (±0.370)	−70.63 (±3.15)	47.01 (±4.90)	−23.62 (±1.20)
	**1k**	−48.87 (±2.71)	−24.26 (±1.22)	56.38 (±3.08)	−6.31 (±0.56)	−73.13 (±2.01)	50.07 (±2.78)	−23.06 (±1.12)

Inhibitors with negative values signify heightened substrate interactions with the targets.

## Data Availability

The data presented in this study are available on request from the corresponding author A.I.-J.

## Supplementary Materials

The following supporting information can be downloaded at: https://www.mdpi.com/article/10.3390/ph16111562/s1, (1) workflow, (2) structures of compounds in vitro assayed, (3) spectra of all new compounds (^1^H NMR, ^13^C NMR, HRMS and FT-IR, (4) in silico study details (docking data and molecular dynamics), and (5) supplementary references.
