# Supplementary material for: Synthesis of Pyrrolo[3,4-b]pyridin-5-ones via Ugi–Zhu Reaction and In Vitro–In Silico Studies against Breast Carcinoma
Source: Pharmaceuticals (Basel). 2023 Nov 6;16(11):1562. doi: 10.3390/ph16111562 (PMC10674953; doi:10.3390/ph16111562)
Supplement: Supplementary file 1 [file pharmaceuticals-16-01562-s001.zip › pharmaceuticals-2685871-supplementary.pdf]

SUPPLEMENTARY MATERIAL

Synthesis of pyrrolo[3,4-*b*]pyridin-5-ones via Ugi-Zhu reaction and In vitro-In silico studies against breast carcinoma

Ivette Morales-Salazar[1], Carlos E. Garduño-Albino[1], Flora P. Montes-Enríquez[1], Eduardo González-Zamora[1], Dania A. Nava-Tapia[2], Napoleón Navarro-Tito[2,*], Leonardo David Herrera-Zúñiga[1,*], and Alejandro Islas-Jácome[1,*]

[1] Departamento de Química, Universidad Autónoma Metropolitana-Iztapalapa, Av. Ferrocarril San Rafael Atlixco 186, Col. Leyes de Reforma 1A Sección, Iztapalapa, Ciudad de México 09310, Mexico; ivette649_tatu@hotmail.com (I.M.-S.); charlywuiller199912101@gmail.com (C.E.G.-A.); montesenriquez98@outlook.com (F.P.M.-E.); egz@xanum.uam.mx (E.G.-Z.)

[2] Laboratorio de Biología Celular del Cáncer, Universidad Autónoma de Guerrero, Chilpancingo de los Bravo 39086, México; danianavatapia@uagro.mx (D.A.N.-T.)

* Correspondence: nnavarro@uagro.mx (N.N.-T.); leo.hz@xanum.uam.mx (L.D.H.-Z.); aij@xanum.uam.mx (A.I.-J)

	CONTENTS	*PAGE*
1	**Workflow**	S3
2	**Compounds *in vitro* assayed**	S4
	• **Table S1.** Compounds *in vitro* assayed	S4
3	**Spectra of new compounds**	S5
	• **Figure S1.** ^{1}H NMR spectrum of the compound **1g**	S5
	• **Figure S2.** ^{13}C NMR spectrum of the compound **1g**	S6
	• **Figure S3.** COSY spectrum of the compound **1g**	S7
	• **Figure S4.** HSQC (^{1}H, ^{13}C) spectrum of the compound **1g**	S8
	• **Figure S5.** HMBC (^{1}H, ^{13}C) spectrum of the compound **1g**	S9
	• **Figure S6.** HRMS spectrum of the compound **1g**	S10
	• **Figure S7.** FT-IR (ATR) spectrum of the compound **1g**	S11
	• **Figure S8.** ^{1}H NMR spectrum of the compound **1h**	S12
	• **Figure S9.** ^{13}C NMR spectrum of the compound **1h**	S13
	• **Figure S10.** HRMS spectrum of the compound **1h**	S14
	• **Figure S11.** FT-IR (ATR) spectrum of the compound **1h**	S15
	• **Figure S12.** ^{1}H NMR spectrum of the compound **1i**	S16
	• **Figure S13.** ^{13}C NMR spectrum of the compound **1i**	S17
	• **Figure S14.** HRMS spectrum of the compound **1i**	S18
	• **Figure S15.** FT-IR (ATR) spectrum of the compound **1i**	S19
	• **Figure S16.** ^{1}H NMR spectrum of the compound **1j**	S20
	• **Figure S17.** ^{13}C NMR spectrum of the compound **1j**	S21
	• **Figure S18.** HRMS spectrum of the compound **1j**	S22
	• **Figure S19.** FT-IR (ATR) spectrum of the compound **1j**	S23
	• **Figure S20.** ^{1}H NMR spectrum of the compound **1k**	S24

	• **Figure S21.** ^{13}C NMR spectrum of the compound **1k**	S25
	• **Figure S22.** HRMS spectrum of the compound **1k**	S26
	• **Figure S23.** FT-IR (ATR) spectrum of the compound **1k**	S27
4	***In silico* studies**	S28
	• **Table S2**: Receptor and Cavity Information	S28
	• **Table S3**. Multiligand and Multitarget Docking Summary of **1a-k** with 20 Target Proteins	S34
	• **Table S4**. Key ADMETox Predicted Properties of the compounds **1a-k**	S45
	• **Table S5**. Structural Time Equilibrium Descriptors	S47
	• **Table S6.** Decomposition of Key Amino Acids for Interaction at 5 Å Calculated Using MM/GBSA	S48
	• **Table S7**. 2D and 3D Representations of Optimized Structures for Ligands **1a-k**	S49
	• **Table S8.** Target Proteins. Ribbon representation of the 20 potential target proteins	S51
	• **Figure S24**. Structure of Protein AKT_1	S55
	• **Figure S25.** Structure of Protein Ox_2R	S56
	• **Figure S26**. Superposition of Representative Structures from Simulations with Ligands **1f**, **1h**, and **1k**	S57
	• **Figure S27.** Blind Docking for Structures 5IU2 and 7NA8	S58
	• **Movie 1**	S60
	• **Movie 2**	S60
5	**Supplementary References**	S60

1. Workflow

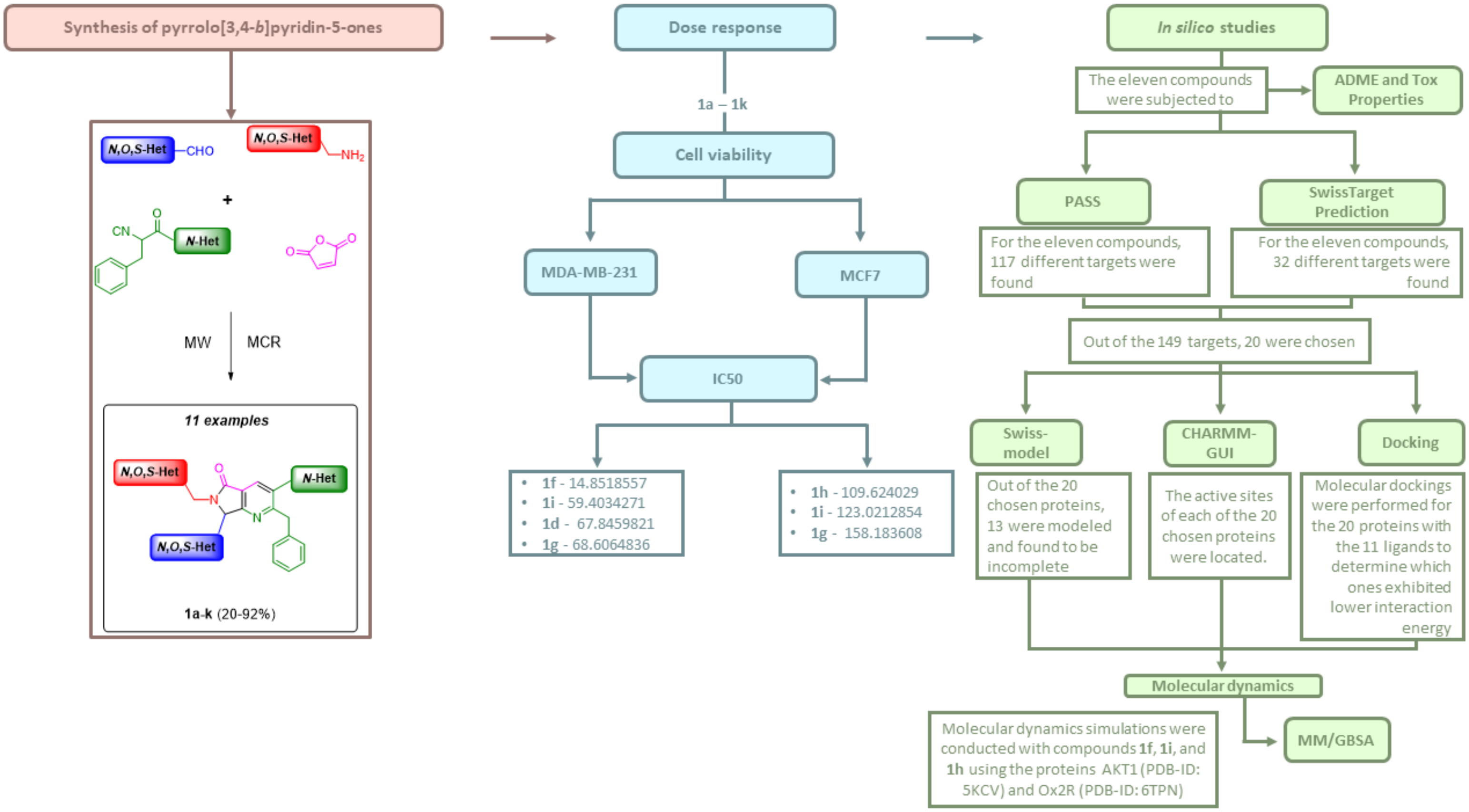

Synthesis of pyrrolo[3,4-b]pyridin-5-ones
N,O,S-Het
CHO
N,O,S-Het
NH2
CN
O
N-Het
MW
MCR
11 examples
N,O,S-Het
N-Het
N,O,S-Het
1a-k (20-92%)
Dose response
1a – 1k
Cell viability
MDA-MB-231
MCF7
IC50
1f - 14.8518557
1i - 59.4034271
1d - 67.8459821
1g - 68.6064836
1h - 109.624029
1i - 123.0212854
1g - 158.183608
In silico studies
The eleven compounds were subjected to
ADME and Tox Properties
PASS
SwissTarget Prediction
For the eleven compounds, 117 different targets were found
For the eleven compounds, 32 different targets were found
Out of the 149 targets, 20 were chosen
Swiss-model
CHARMM-GUI
Docking
Out of the 20 chosen proteins, 13 were modeled and found to be incomplete
The active sites of each of the 20 chosen proteins were located.
Molecular dockings were performed for the 20 proteins with the 11 ligands to determine which ones exhibited lower interaction energy
Molecular dynamics
Molecular dynamics simulations were conducted with compounds 1f, 1i, and 1h using the proteins AKT1 (PDB-ID: 5KCV) and Ox2R (PDB-ID: 6TPN)
MM/GBSA

2 Compounds *in vitro* assayed

**Table S1.** Compounds *in vitro* assayed	
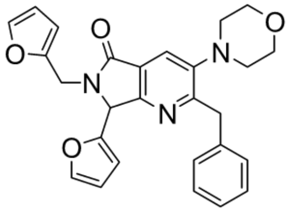 **1a** [Ref. S1]	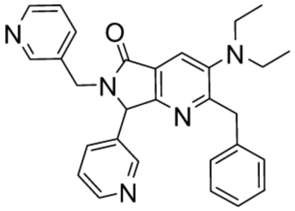 **1g** [New Compound]
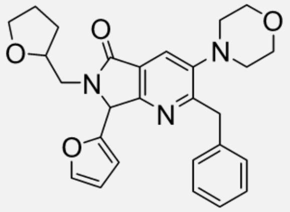 **1b** [Ref. S1]	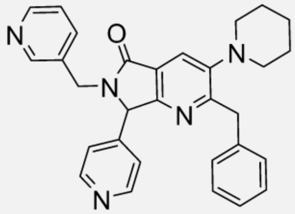 **1h** [New Compound]
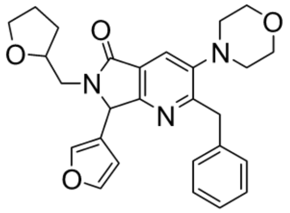 **1c** [Ref. S1]	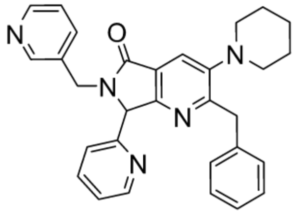 **1i** [New Compound]
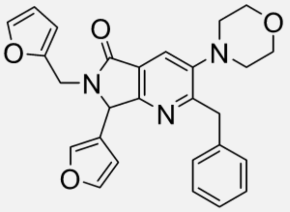 **1d** [Ref. S1]	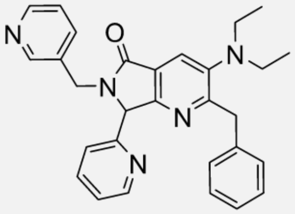 **1j** [New Compound]
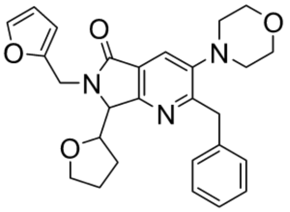 **1e** [Ref. S1]	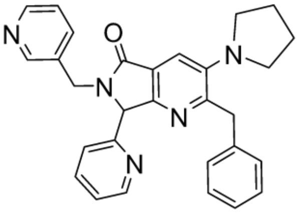 **1k** [New Compound]
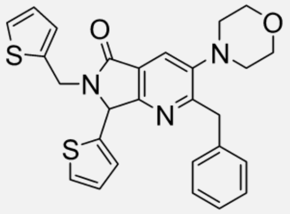 **1f** [Ref. S2]	

3 Spectra of new compounds

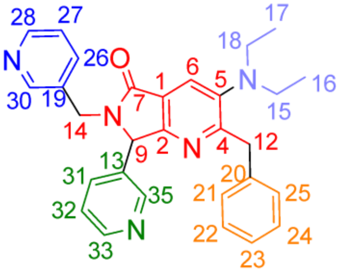

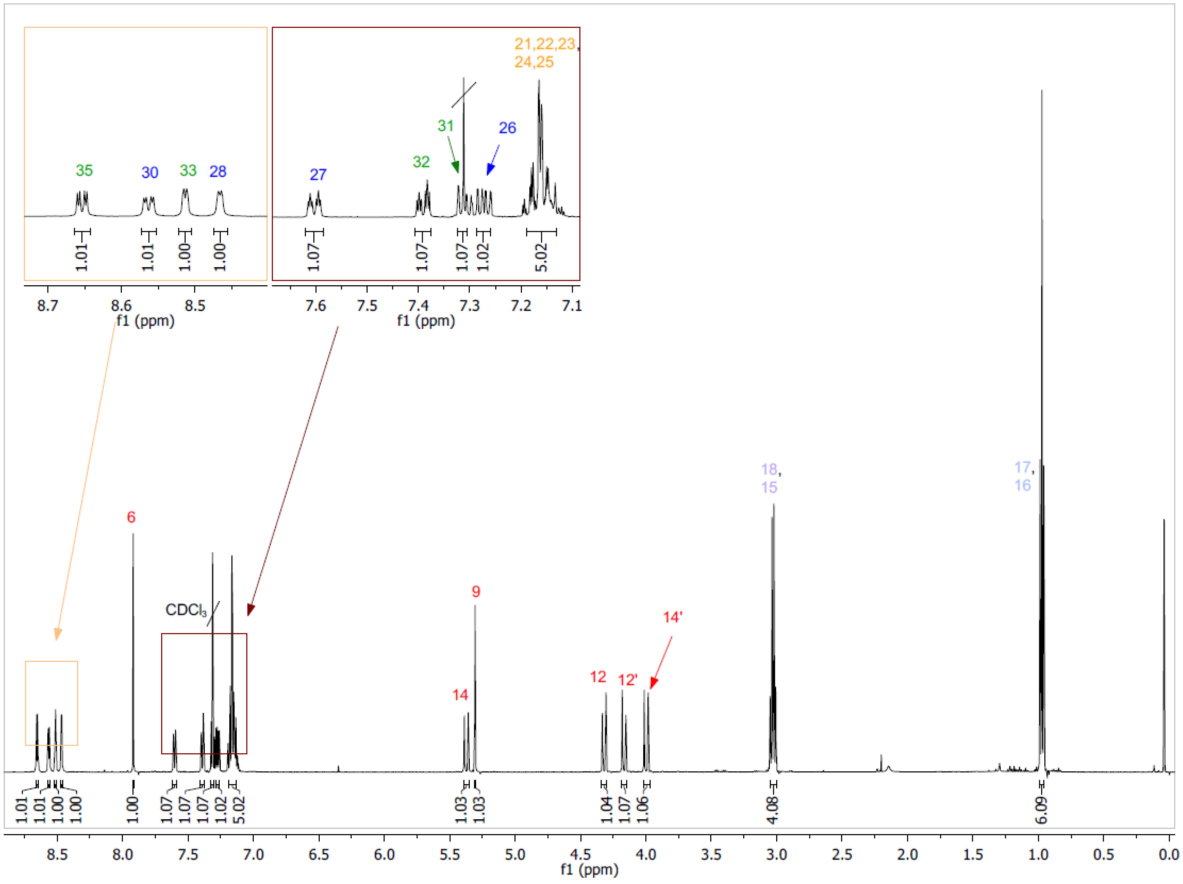

Figure S1. ^{1}H NMR spectrum of the compound **1g**

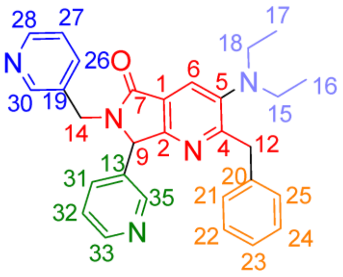

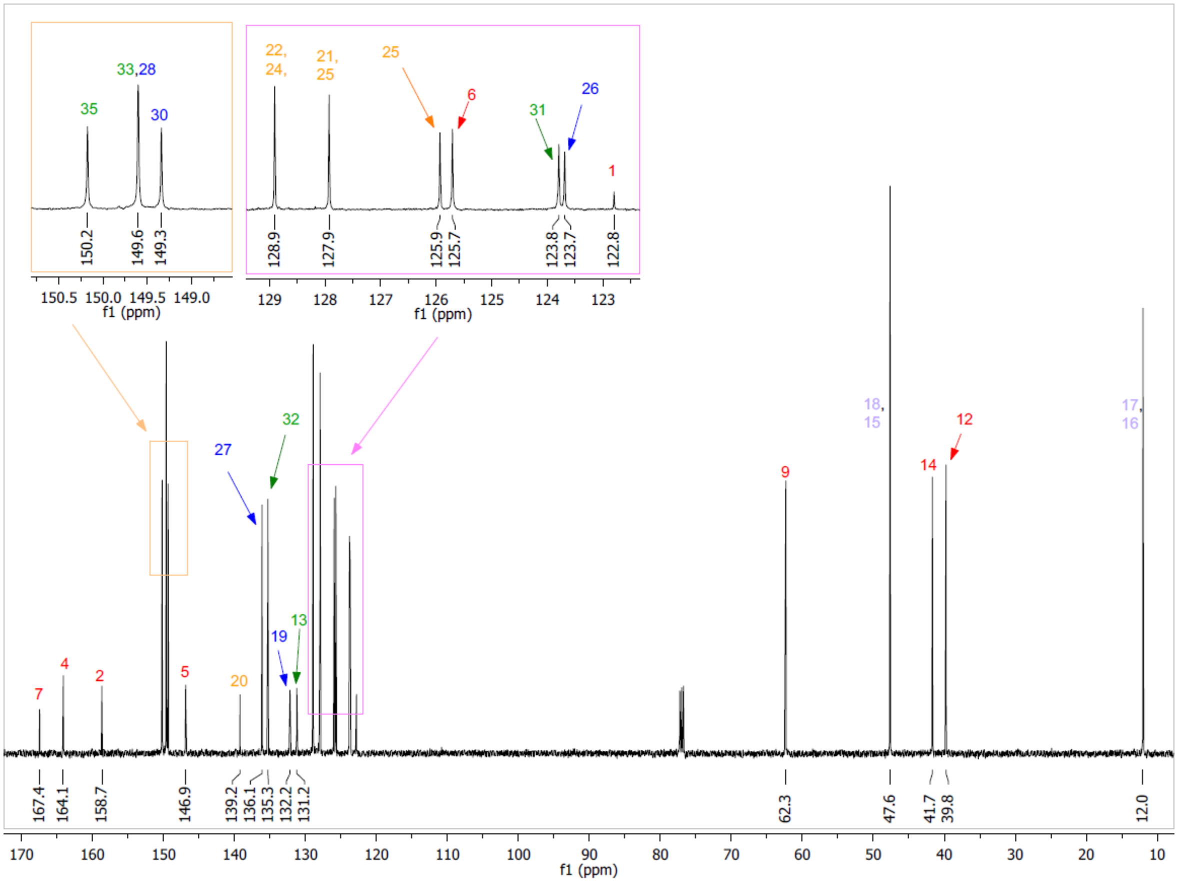

Figure S2. ^{13}C NMR spectrum of the compound **1g**

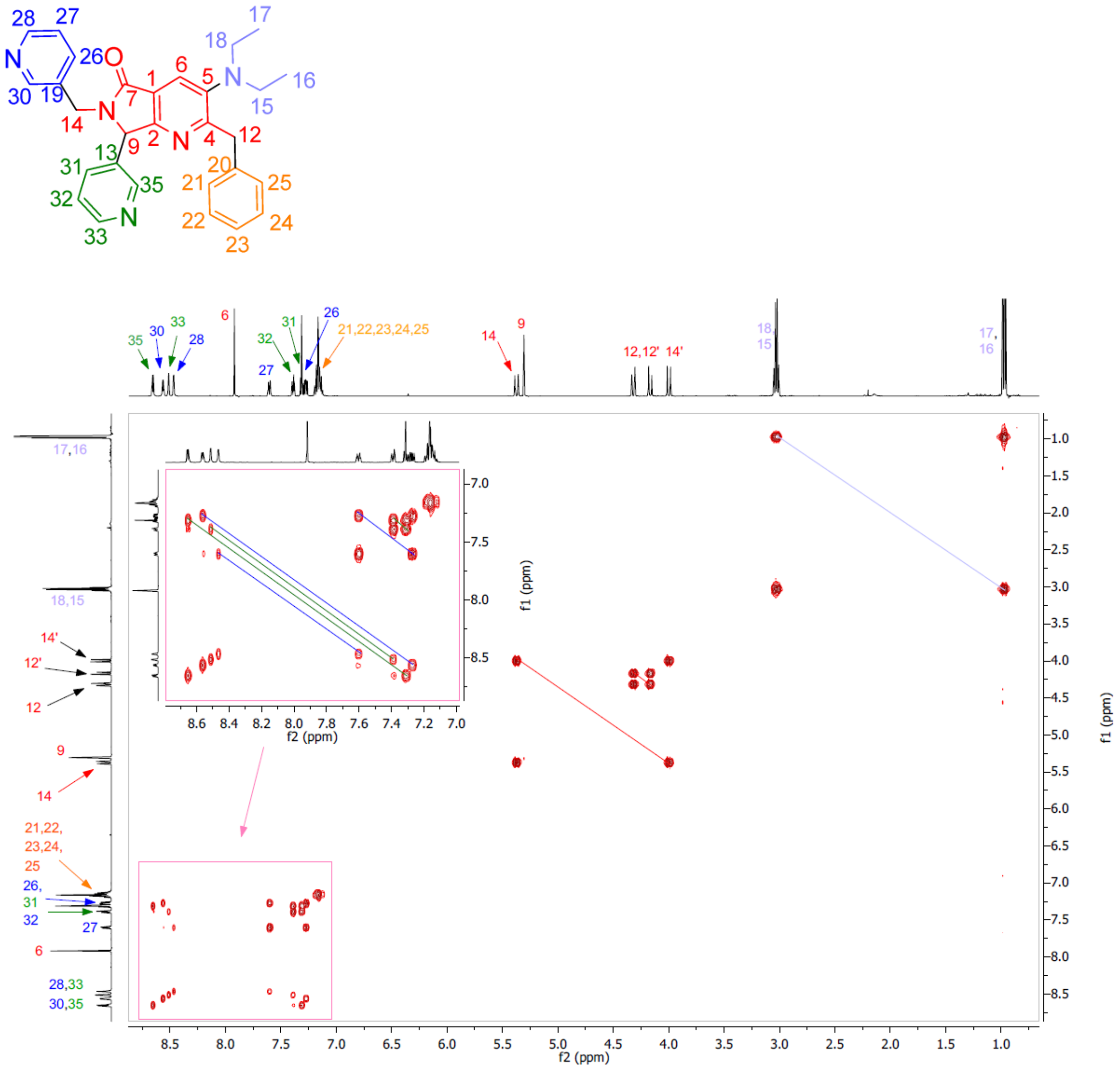

Figure S3. COSY spectrum of the compound **1g**

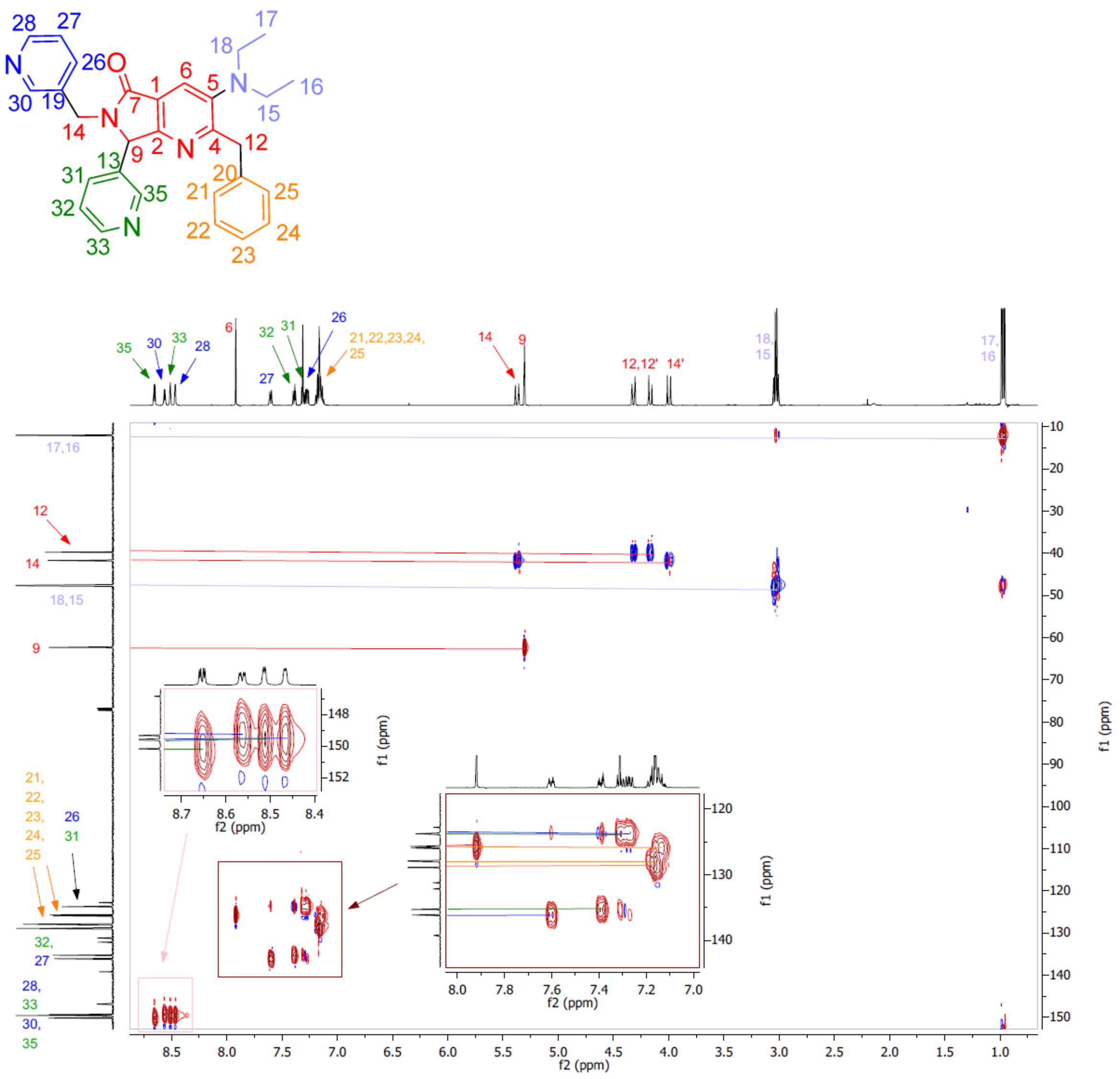

Figure S4. HSQC (^{1}H, ^{13}C) spectrum of the compound **1g**

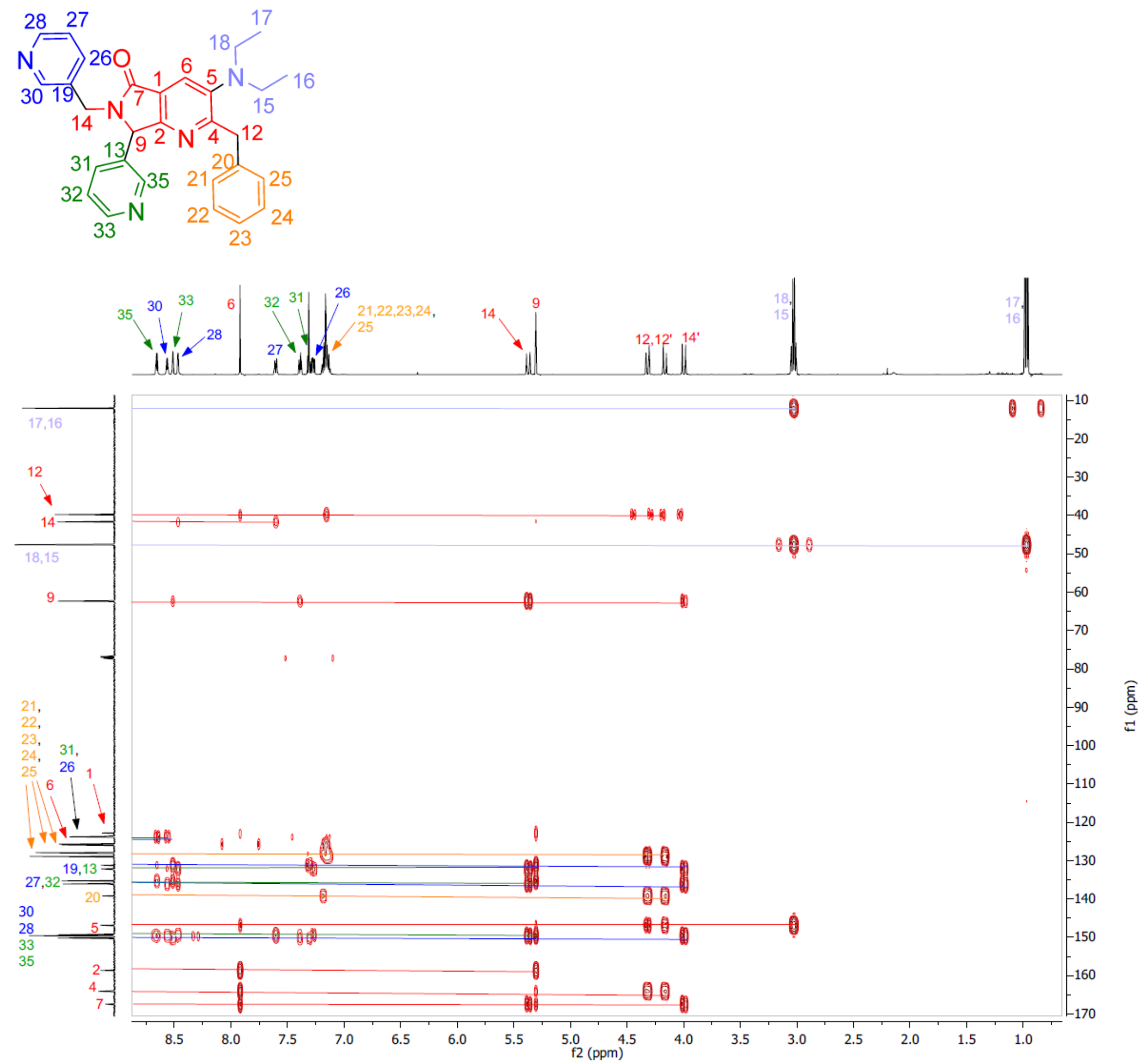

Figure S5. HMBC (^{1}H, ^{13}C) spectrum of the compound **1g**

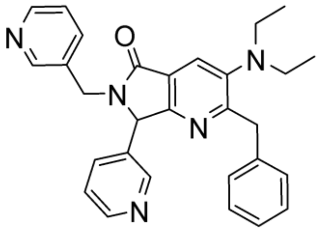

1g

Acquisition Parameter

Source Type	ESI	Ion Polarity	Positive	Set Nebulizer	0.5 Bar
Focus	Active			Set Dry Heater	150 °C
Scan Begin	50 m/z	Set Capillary	4500 V	Set Dry Gas	4.0 l/min
Scan End	3000 m/z	Set End Plate Offset	-500 V	Set Divert Valve	Waste

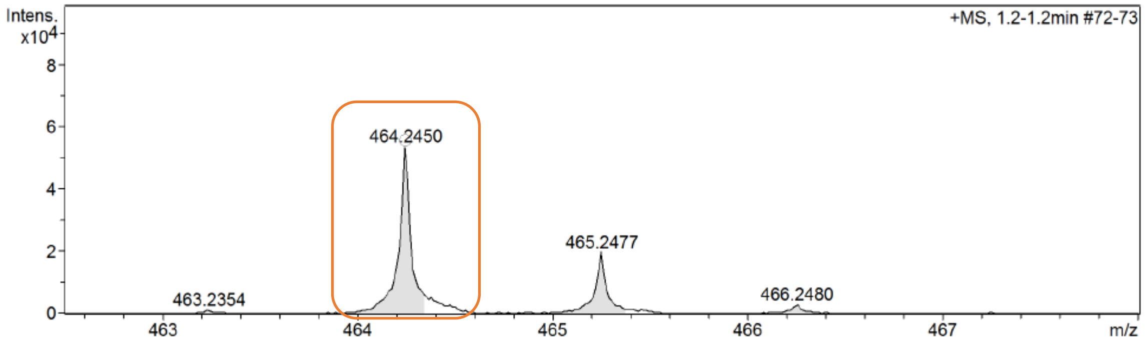

Meas. m/z	#	Ion Formula	Sum Formula	m/z	err [ppm]	|err| [mDa]	mSigma	# Sigma
464.2450	1	C29H30N5O	C29 H29 N5 O	464.2445	1.2	0.5	15.2	2

Figure S6. HRMS spectrum of the compound **1g**

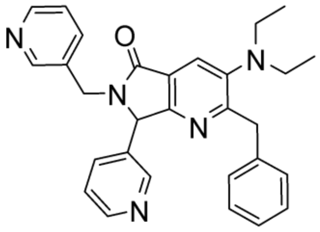

1g

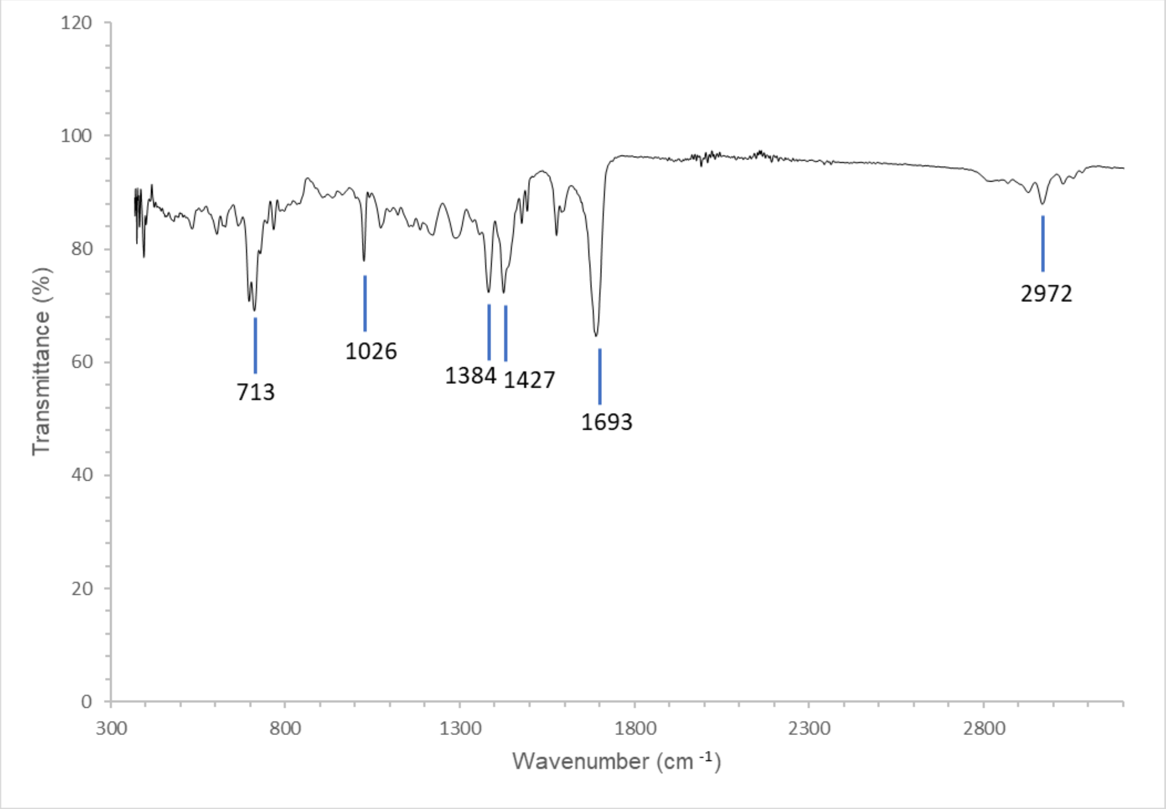

Figure S7. FT-IR (ATR) spectrum of the compound **1g**

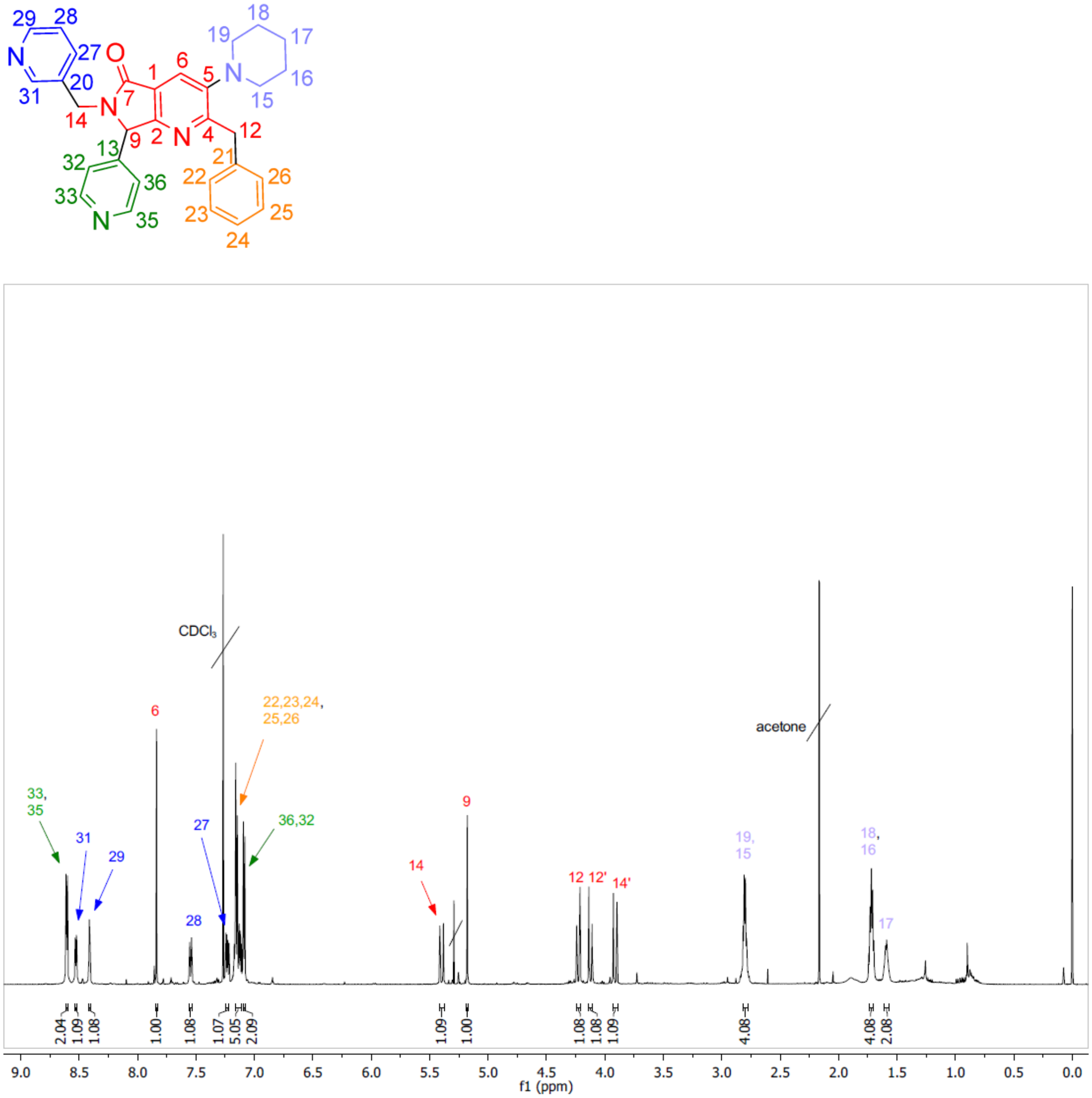

Figure S8. ^{1}H NMR spectrum of the compound **1h**

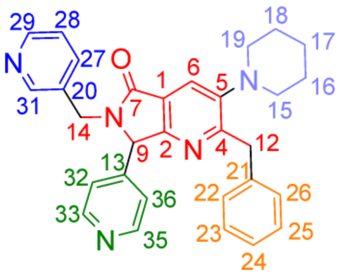

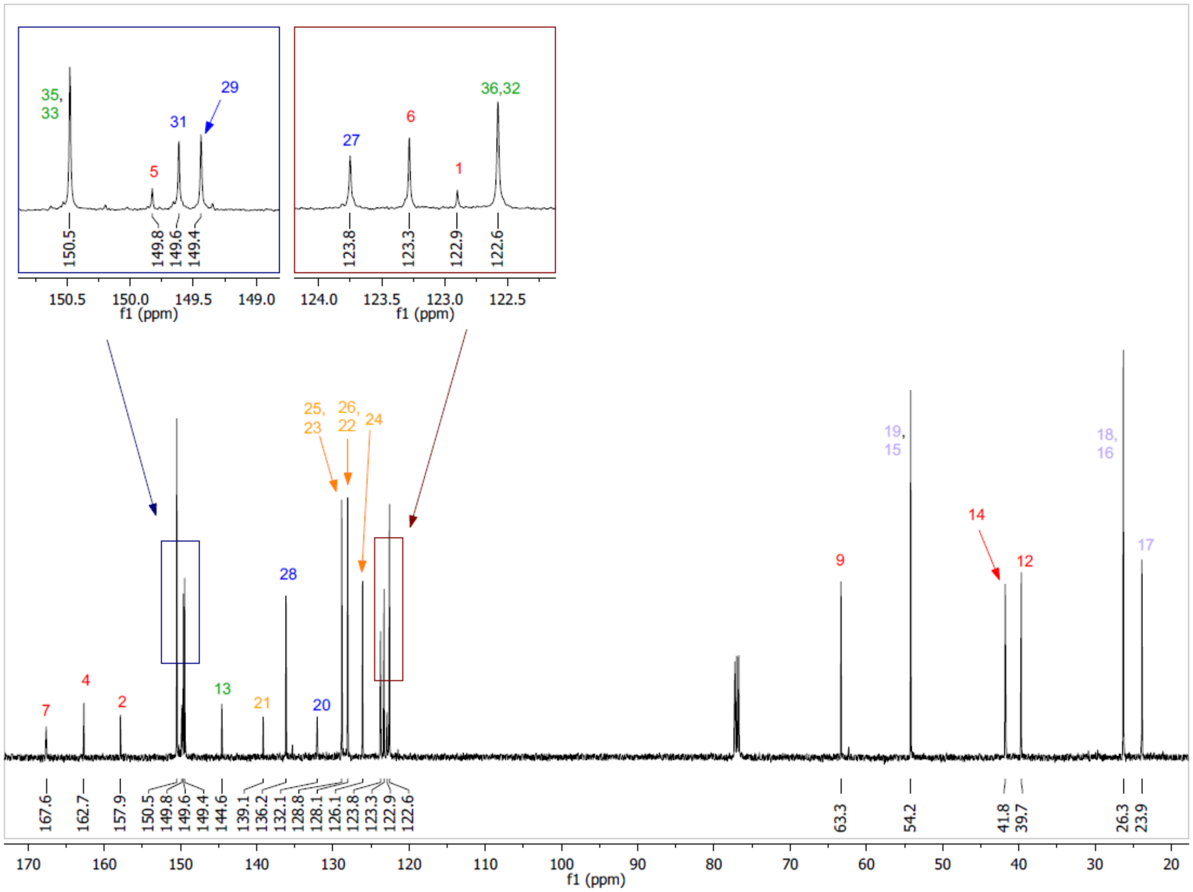

Figure S9. ^{13}C NMR spectrum of the compound **1h**

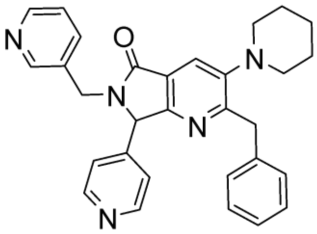

1h

Acquisition Parameter

Source Type	ESI	Ion Polarity	Positive	Set Nebulizer	0.5 Bar
Focus	Active			Set Dry Heater	150 °C
Scan Begin	50 m/z	Set Capillary	4500 V	Set Dry Gas	4.0 l/min
Scan End	3000 m/z	Set End Plate Offset	-500 V	Set Divert Valve	Waste

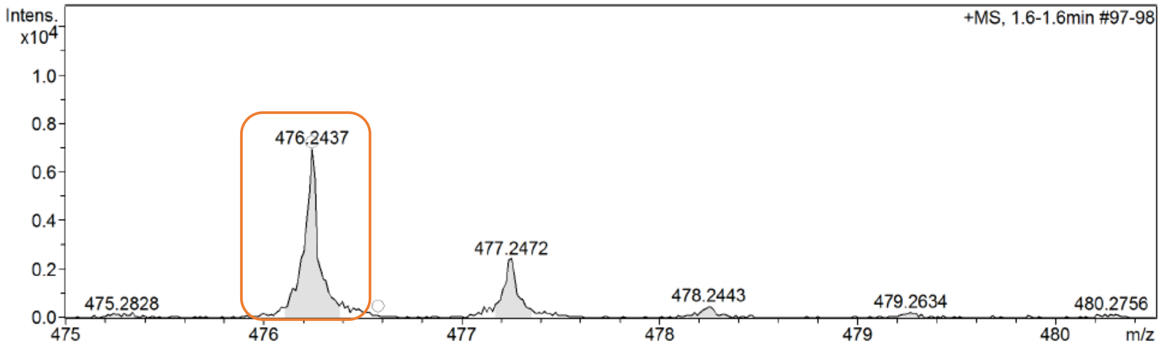

Meas. m/z	#	Ion Formula	Sum Formula	m/z	err [ppm]	|err| [mDa]	mSigma	# Sigma
476.2437	1	C30H30N5O	C30 H29 N5 O	476.2445	1.7	0.8	13.1	2

Figure S10. HRMS spectrum of the compound **1h**

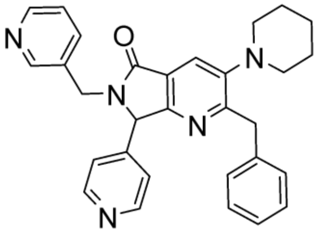

1h

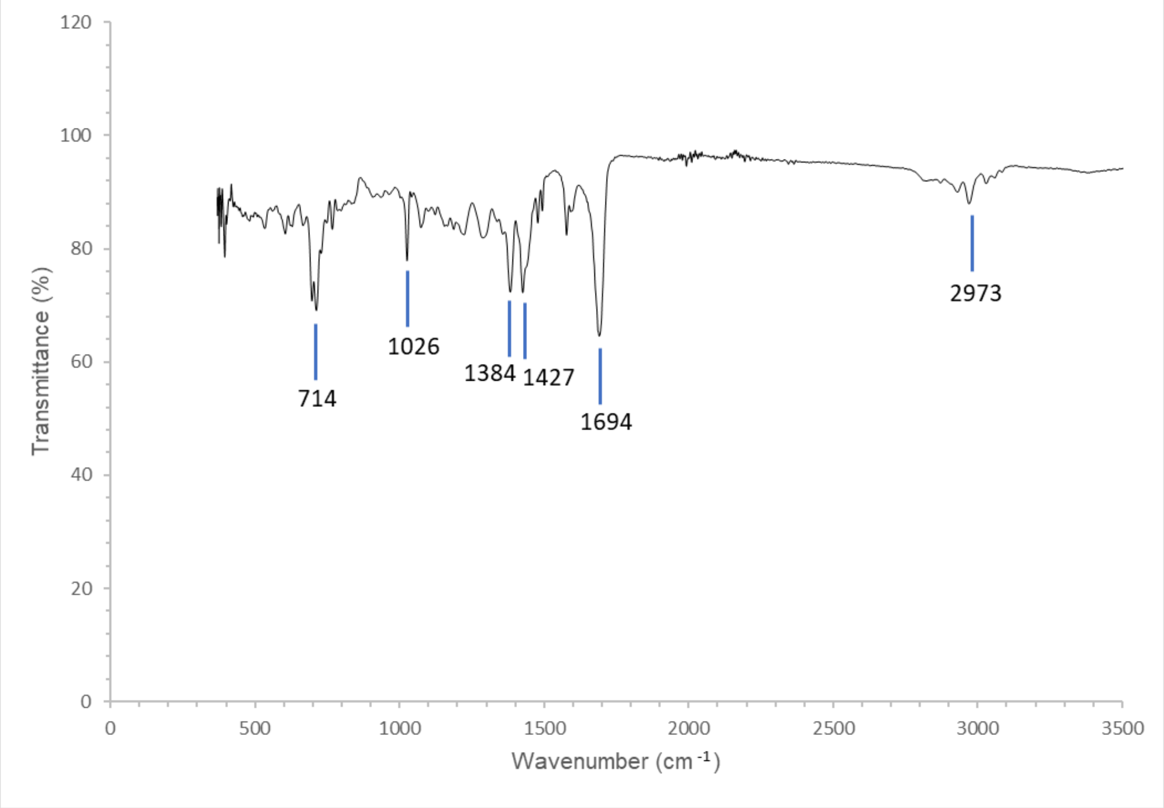

Figure S11. FT-IR (ATR) spectrum of the compound **1h**

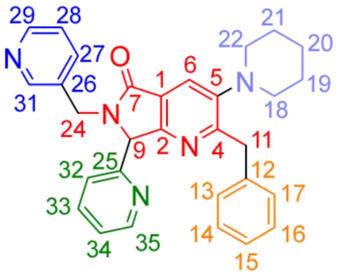

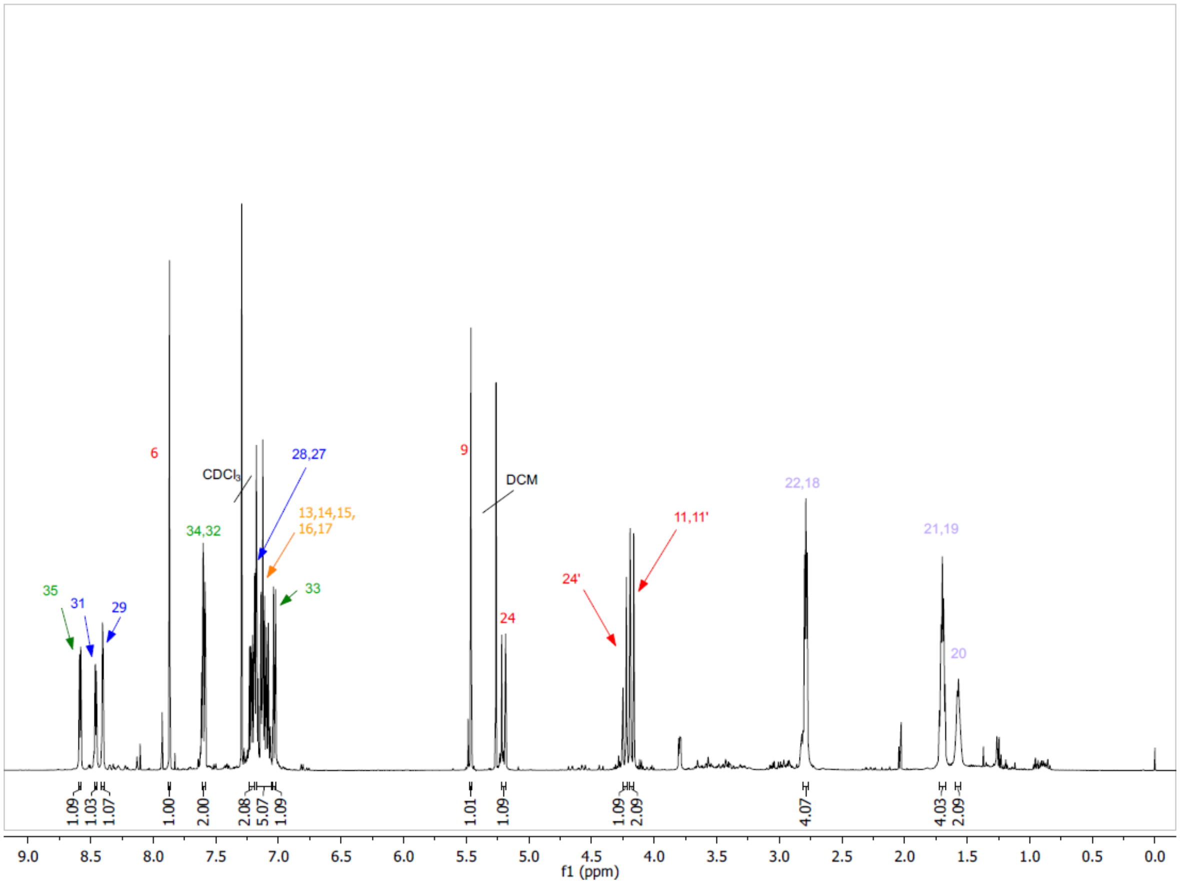

Figure S12. ^{1}H NMR spectrum of the compound **1i**

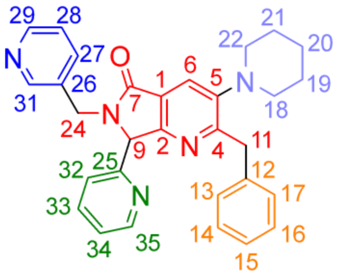

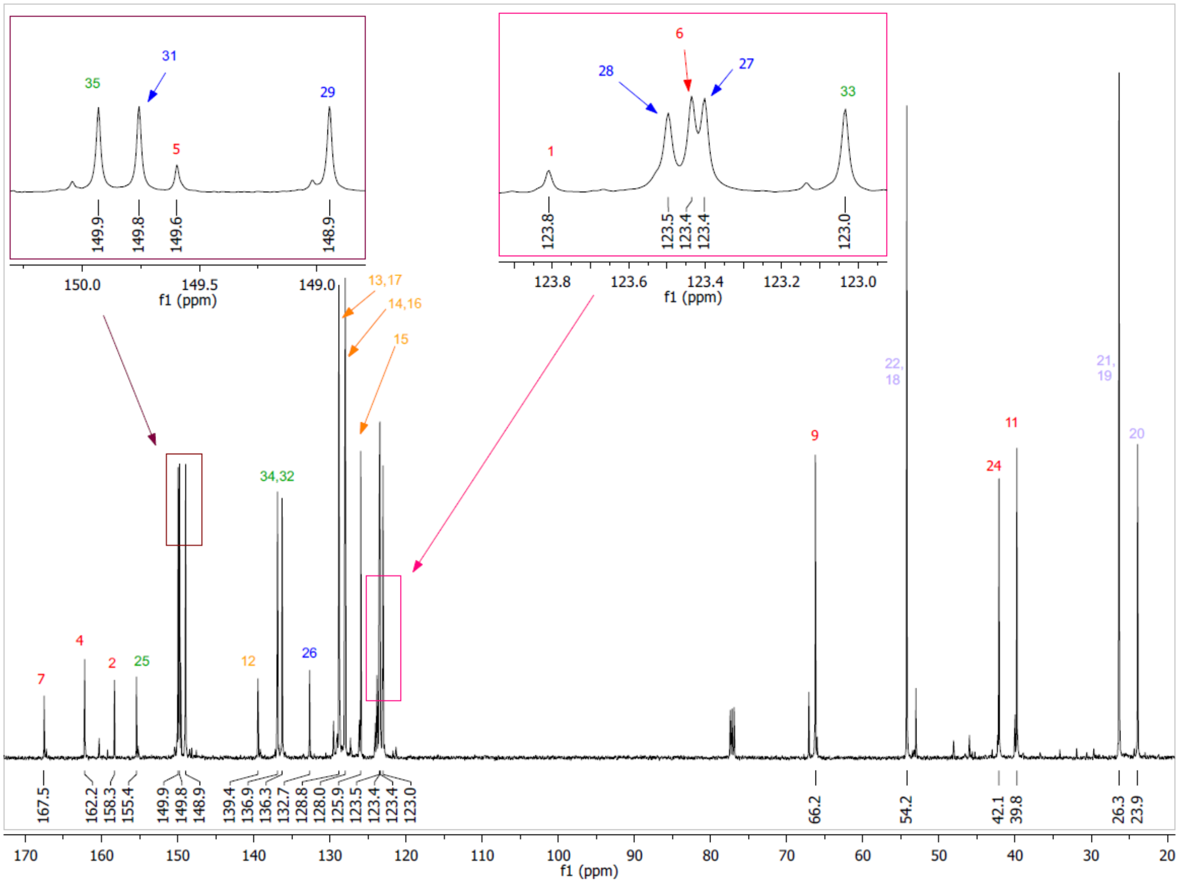

Figure S13. ^{13}C NMR spectrum of the compound **1i**

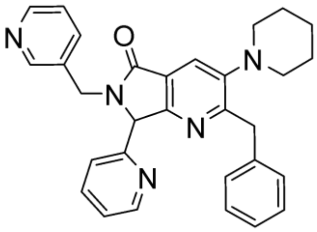

1i

Acquisition Parameter

Source Type	ESI	Ion Polarity	Positive	Set Nebulizer	0.5 Bar
Focus	Active			Set Dry Heater	150 °C
Scan Begin	50 m/z	Set Capillary	4500 V	Set Dry Gas	4.0 l/min
Scan End	3000 m/z	Set End Plate Offset	-500 V	Set Divert Valve	Waste

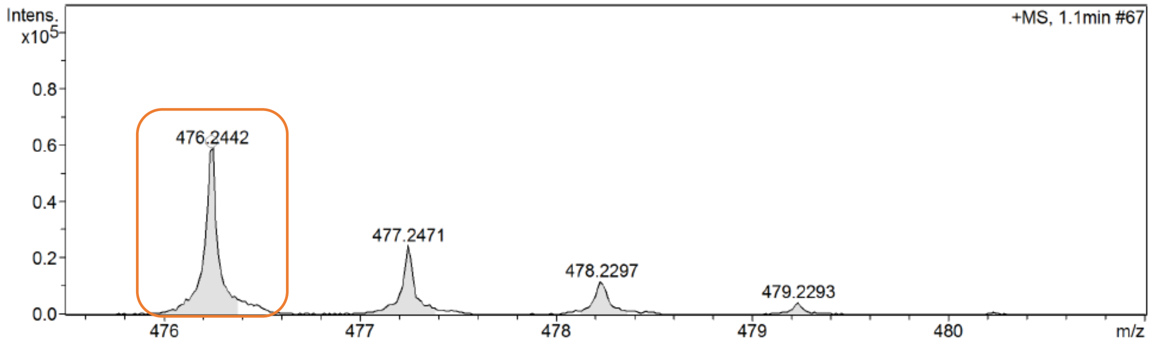

Meas. m/z	#	Ion Formula	Sum Formula	m/z	err [ppm]	|err| [mDa]	mSigma	# Sigma
476.2442	1	C30H30N5O	C30 H29 N5 O	476.2445	-0.5	0.2	83.4	2

Figure S14. HRMS spectrum of the compound **1i**

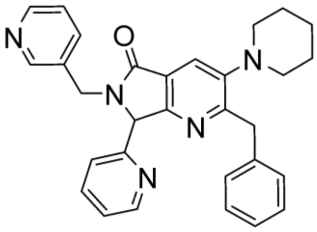

1i

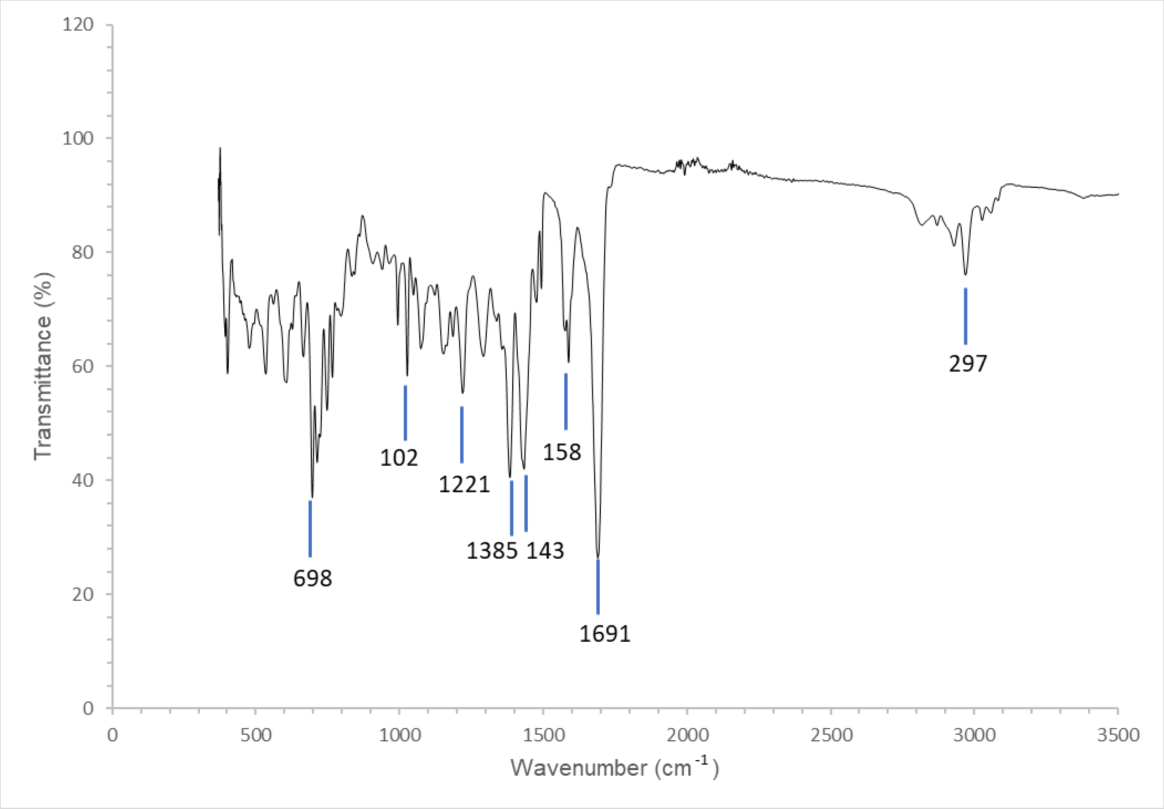

Figure S15. FT-IR (ATR) spectrum of the compound **1i**

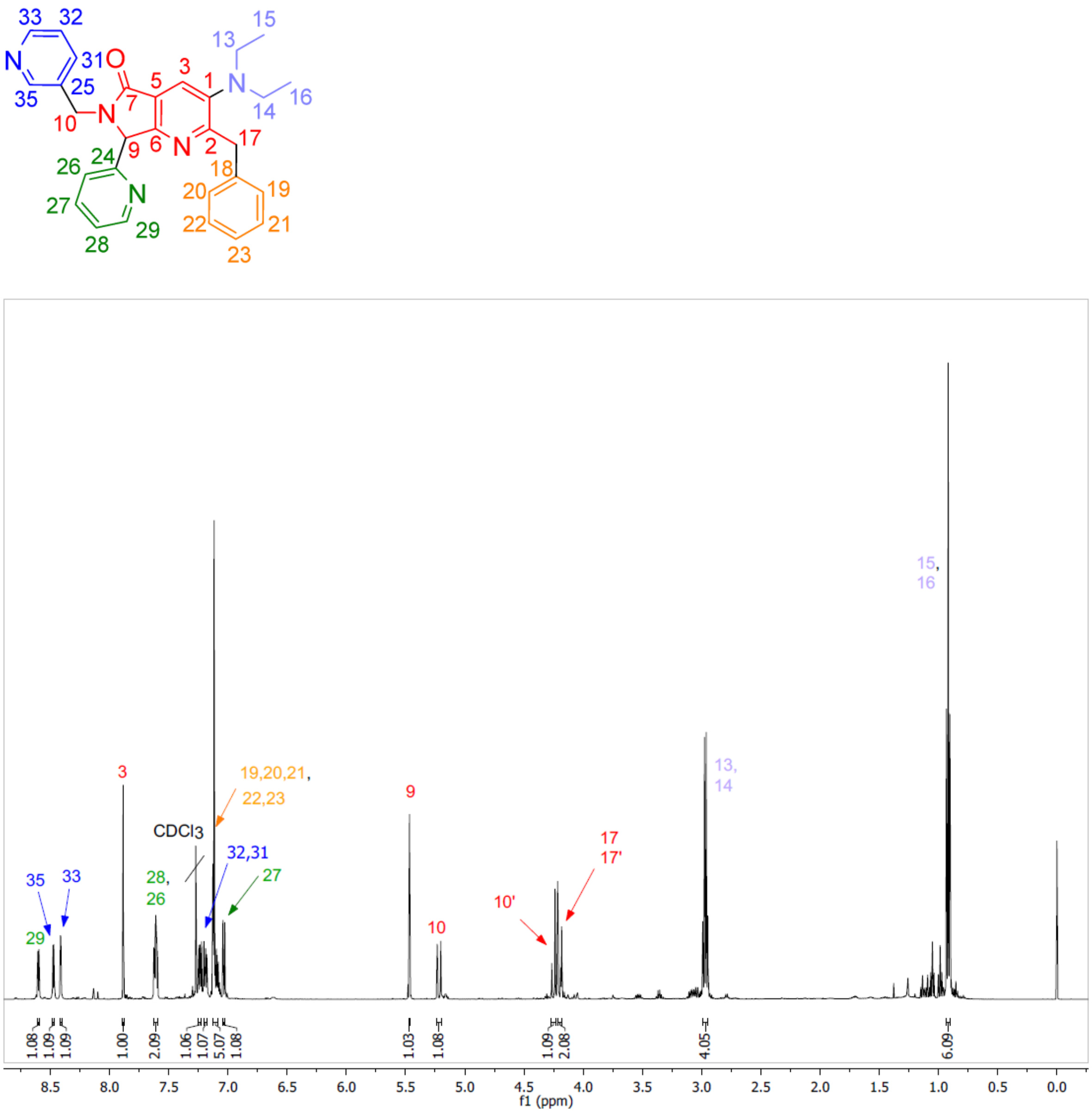

Figure S16. ^{1}H NMR spectrum of the compound **1j**

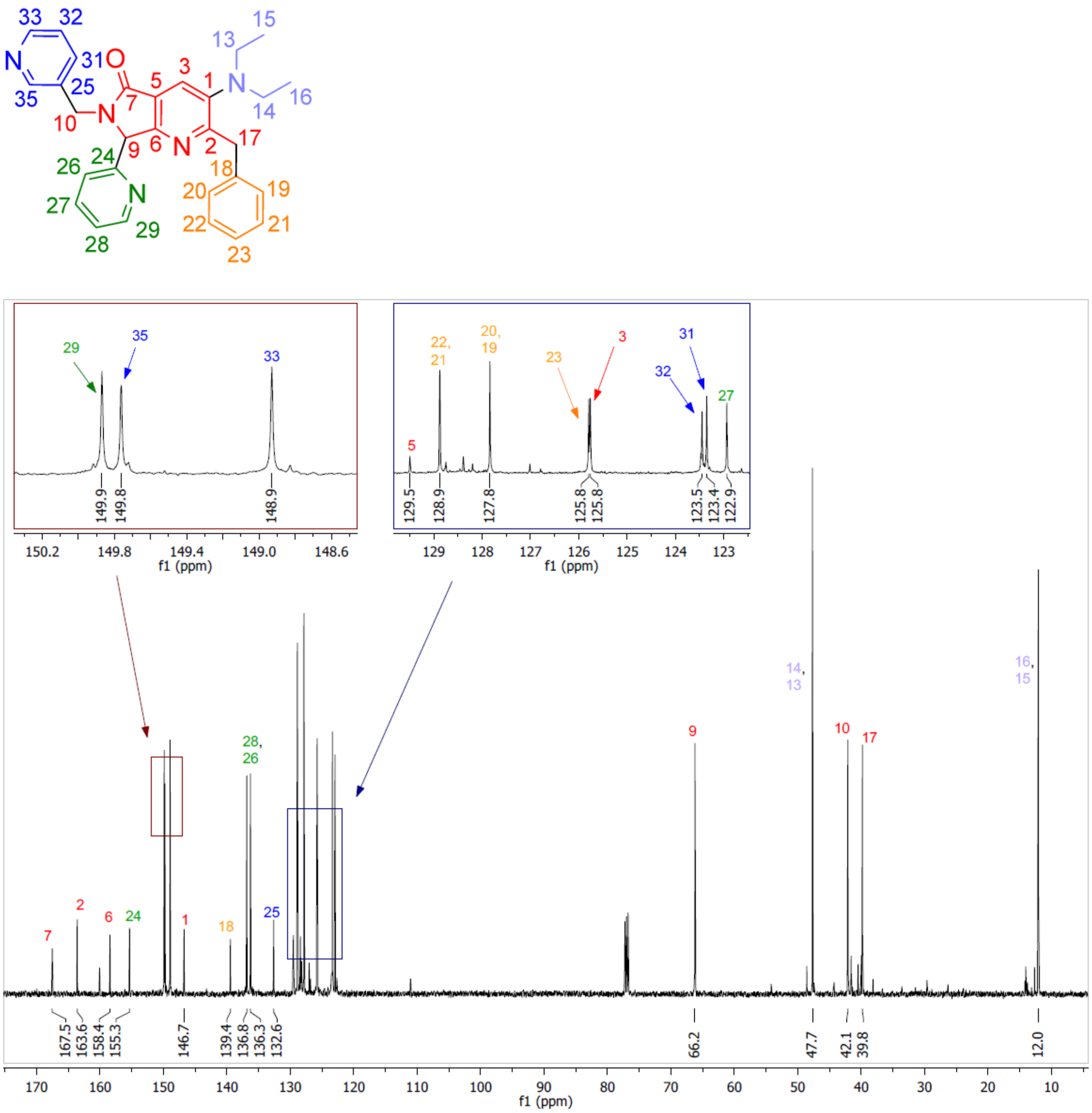

Figure S17. ^{13}C NMR spectrum of the compound **1j**

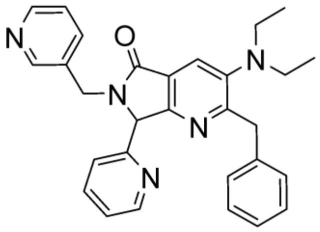

1j

Acquisition Parameter

Source Type	ESI	Ion Polarity	Positive	Set Nebulizer	0.5 Bar
Focus	Active			Set Dry Heater	150 °C
Scan Begin	50 m/z	Set Capillary	4500 V	Set Dry Gas	4.0 l/min
Scan End	3000 m/z	Set End Plate Offset	-500 V	Set Divert Valve	Waste

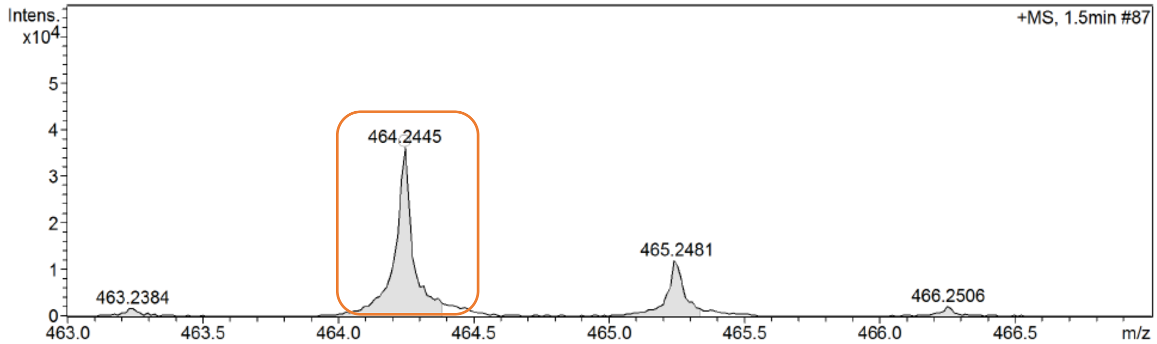

Meas. m/z	#	Ion Formula	Sum Formula	m/z	err [ppm]	|err| [mDa]	mSigma	# Sigma
464.2445	1	C29H30N5O	C29 H29 N5 O	464.2445	-0.1	0.1	1.9	1

Figure S18. HRMS spectrum of the compound **1j**

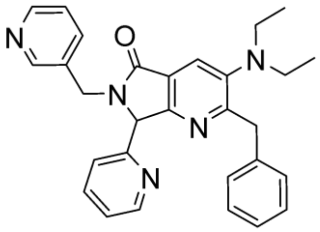

1j

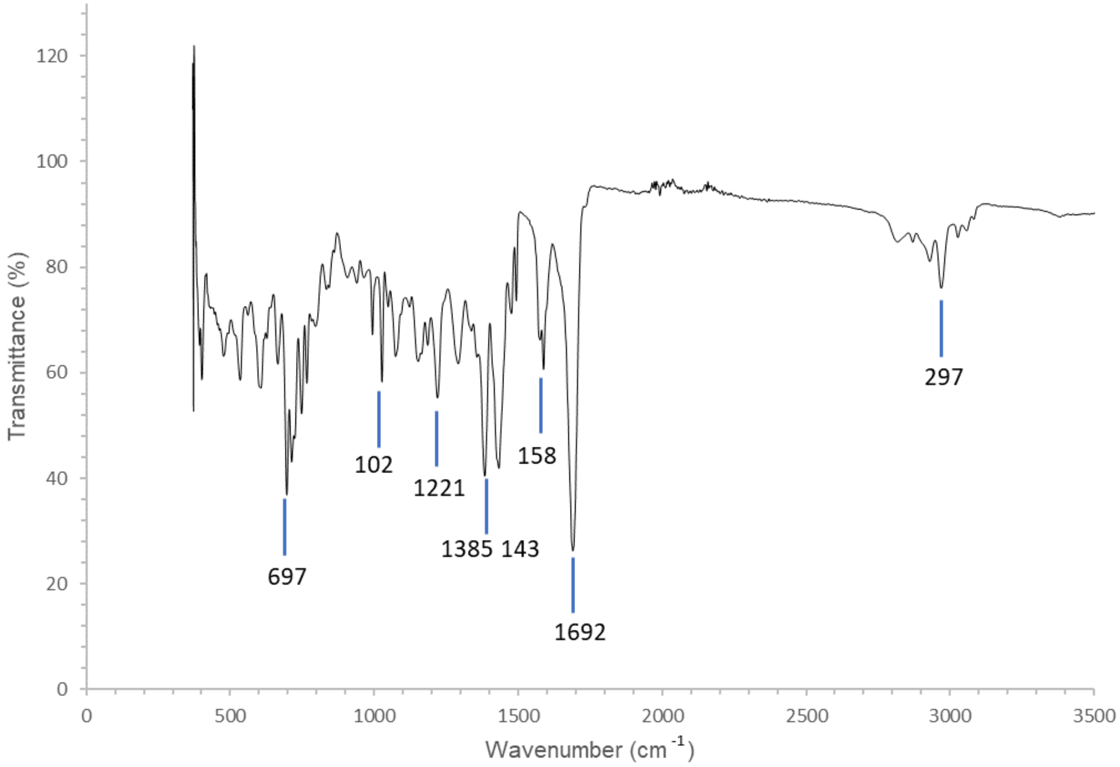

Figure S19. FT-IR (ATR) spectrum of the compound **1j**

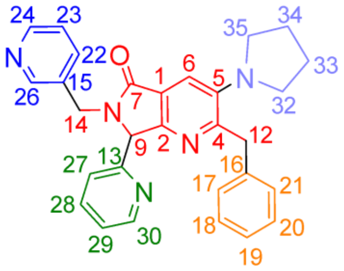

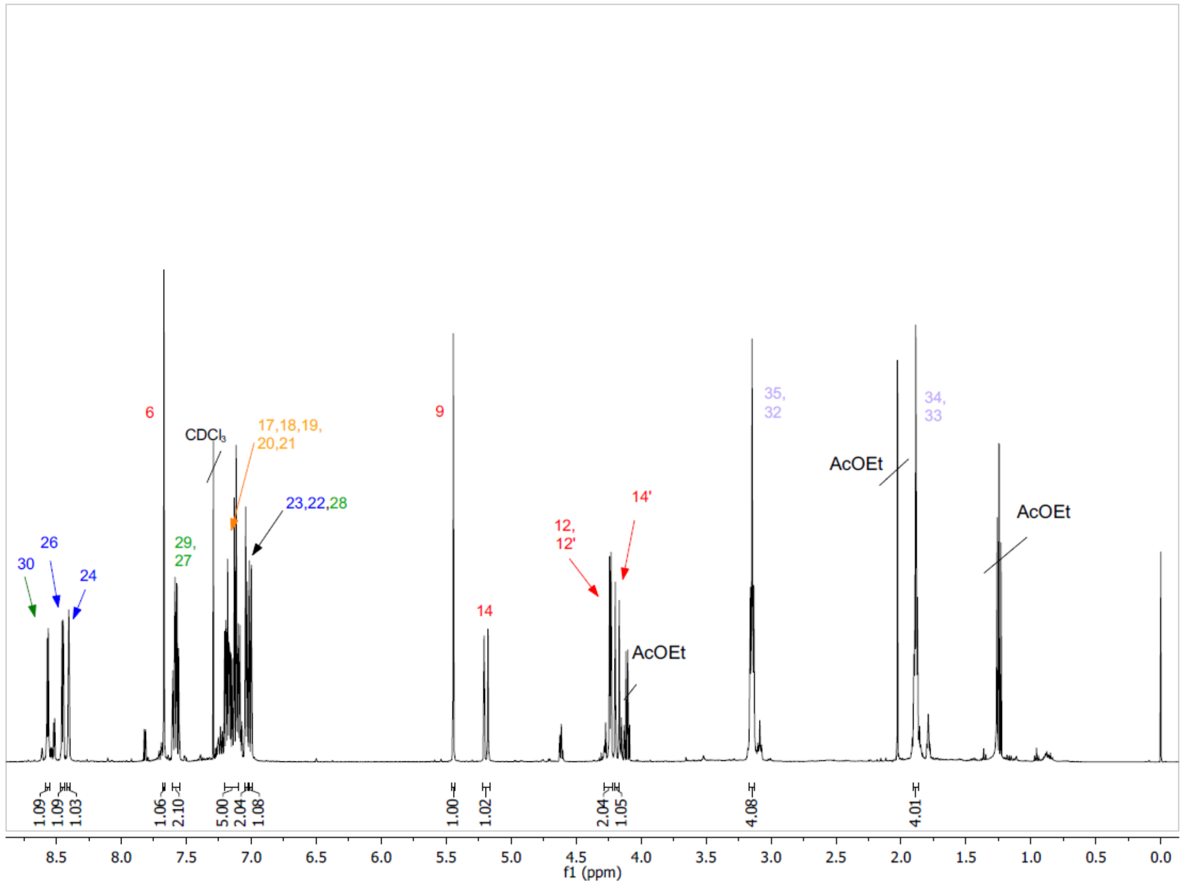

Figure S20. ^{1}H NMR spectrum of the compound **1k**

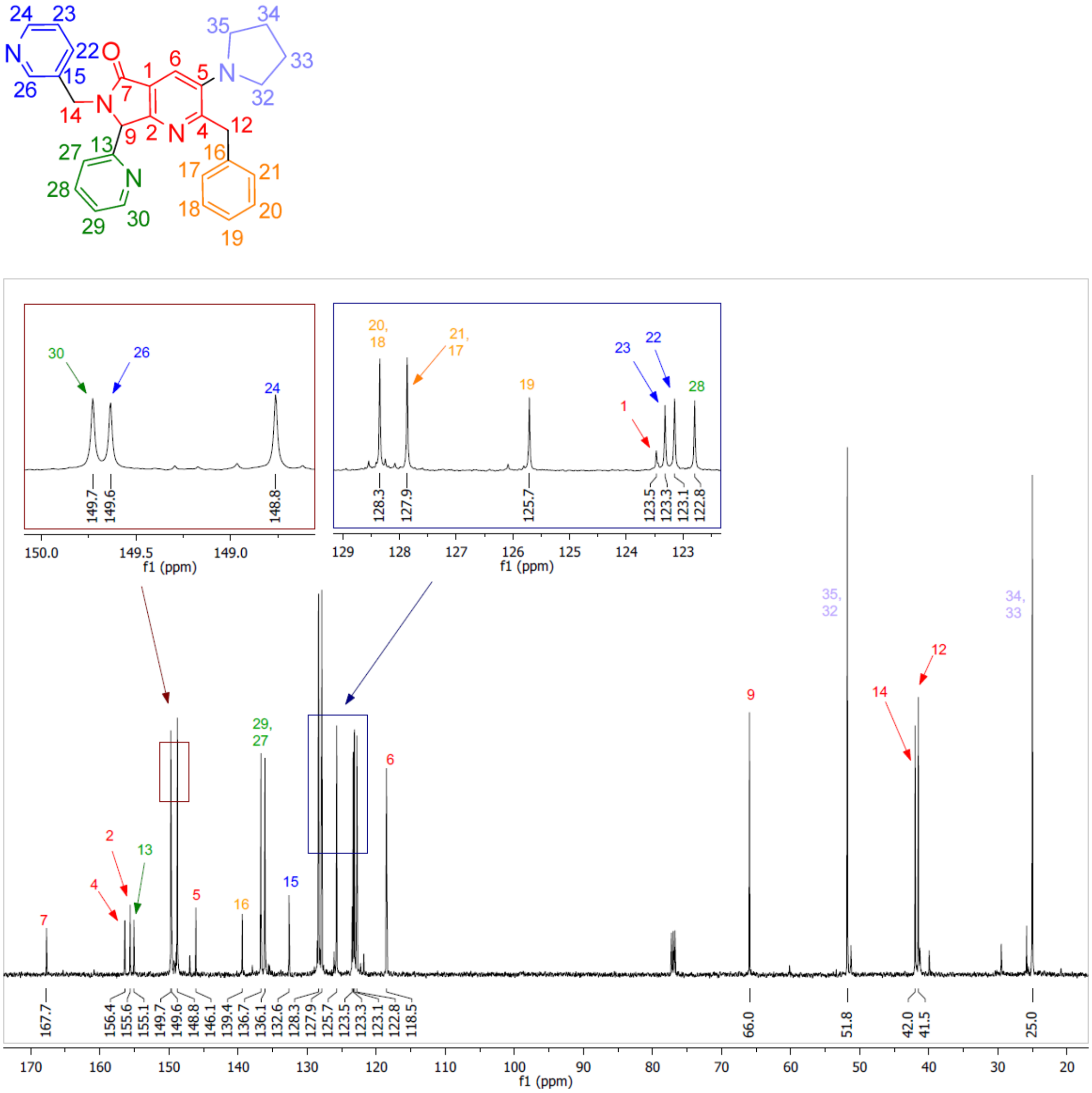

Figure S21. ^{13}C NMR spectrum of the compound **1k**

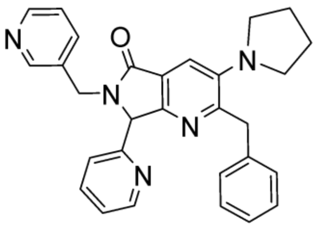

1k

Acquisition Parameter

Source Type	ESI	Ion Polarity	Positive	Set Nebulizer	0.5 Bar
Focus	Active			Set Dry Heater	150 °C
Scan Begin	50 m/z	Set Capillary	4500 V	Set Dry Gas	4.0 l/min
Scan End	3000 m/z	Set End Plate Offset	-500 V	Set Divert Valve	Waste

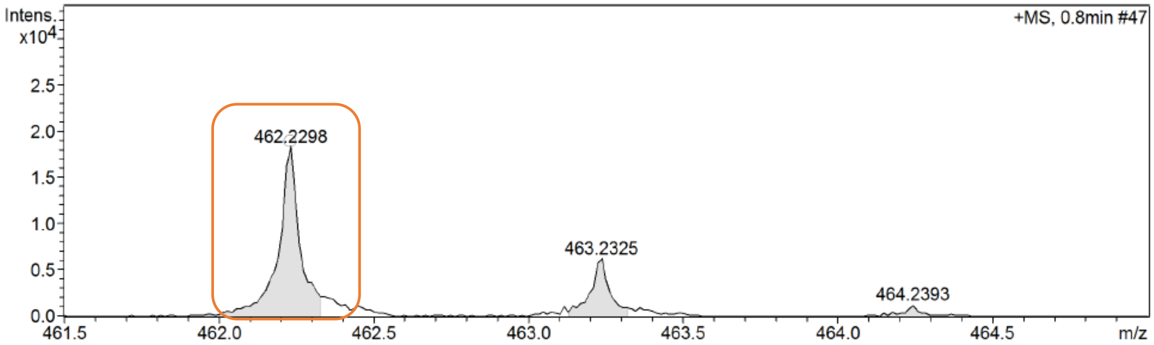

Meas. m/z	#	Ion Formula	Sum Formula	m/z	err [ppm]	err [mDa]	mSigma	# Sigma
462.2298	1	C29H28N5O	C29 H27 N5 O	462.2288	2.0	0.9	6.8	1

Figure S22. HRMS spectrum of the compound **1k**

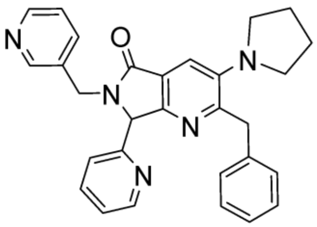

1k

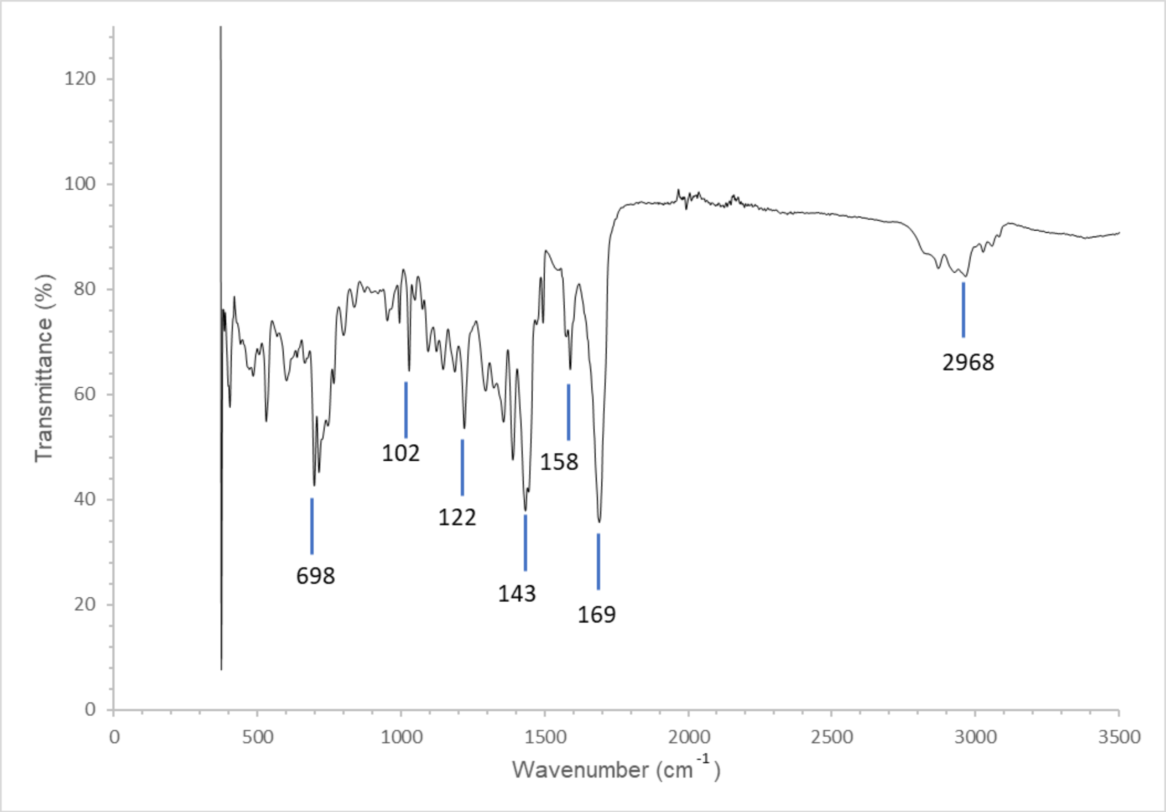

Figure S23. FT-IR (ATR) spectrum of the compound **1k**

4 *In silico* **studies**

Table S2: Receptor and Cavity Information. The **Table S2** contains comprehensive information regarding the receptors and their corresponding cavities. The columns of the dataset consist of several key attributes, including names and acronyms of the targets, PDB IDs, hydrophobic and electrostatic representations of the cavity, identified amino acids within the cavity, and dimensions of the docking box, including its matching centers.

Protein	PDB-ID	Cavity location and representation		Cavity conformation	Docking Box	
		Hydrophobic Surface	Electrostatic Surface	Amino Acids	Size	Center
Dual specificity protein phosphatase 3 (DUSP3)	3F81	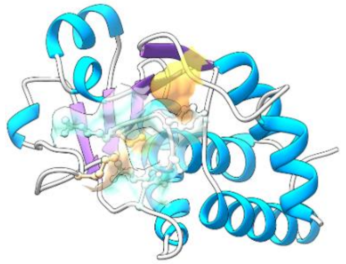	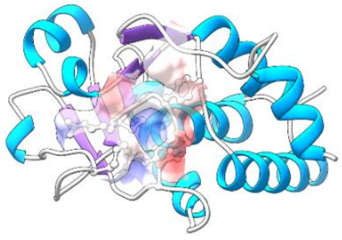	**CHAIN A** LEU25; MET69; ASP92; CYS24; GLU26; GLY27; TYR28; SER29; ARG30; SER31	X: 20.25<br>Y: 20.00<br>Z: 10.00	X: 1.63<br>Y: 2.26<br>Z: -3.4
MAP kinase-activated protein kinase 3 (MAPK3)	3FHR	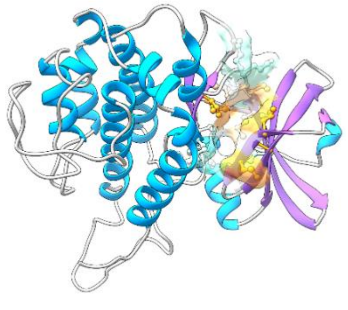	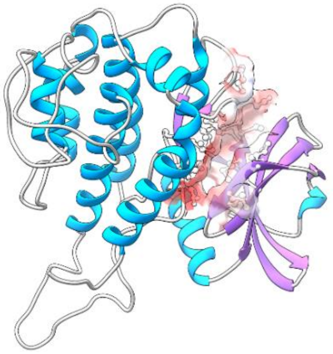	**CHAIN A** LEU50; GLY51; LEU52; GLY53; VAL58; ALA71; LYS73; VAL98; MET118; GLU119; CYS120; MET121; GLU122; GLY124; GLU170; ASN171; LEU173; LYS177; THR 186; ASP187	X: 22.30<br>Y: 18.08<br>Z: 25.23	X: 20.15<br>Y: 3.63<br>Z: 13.75
Orexin receptor type 2 (Ox2R).	6TPN	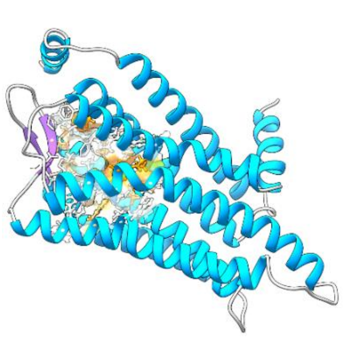	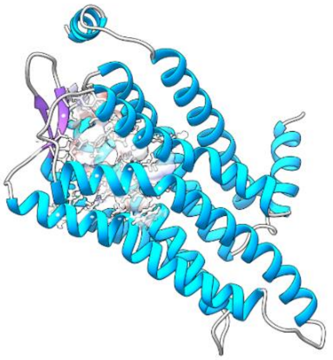	**CHAIN A** CYS107; THR111; VAL114; TRP 20; ILE 130; PRO131; GLN134; THR135; VAL138; GLN187; MET191; CYS210; GLU212; TYR223; HIS224; PHE227; THR231; TYR 232; TYR317; ILE320; SER321; ASN324; HIS350; VAL353; TYR354;	X: 28.13<br>Y: 21.75<br>Z: 21.75	X: 95.48<br>Y: 225.08<br>Z: 2.25

Metabotropic glutamate receptor 2 (mGluR2)	7EPF	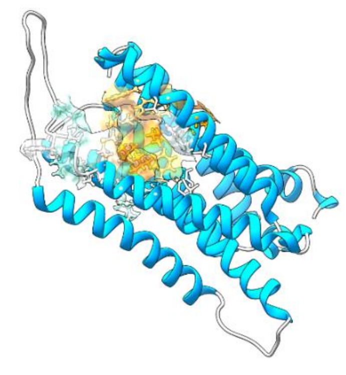	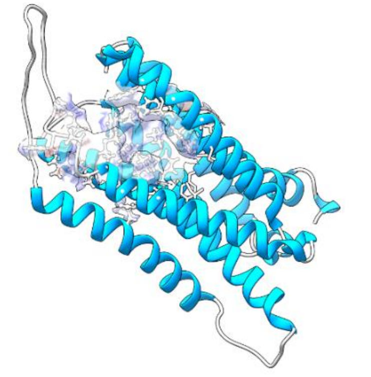	**CHAIN A**<br>PHE623; ARG636<br>LEU 639; GLY640;<br>PHE643; GLN690;<br>HIS723; ARG724;<br>ASP725; SER727;<br>MET728; SER731;<br>LEU732; ASN735;<br>ILE772; TRP773;<br>PHE776; PHE780;<br>MET794; SER797;<br>VAL798; SER801	X: 20.59<br>Y: 26.05<br>Z: 21.02	X: 13.83<br>Y: -50.33<br>Z: 41.76
Histamine H3 receptor (H3)	7F61	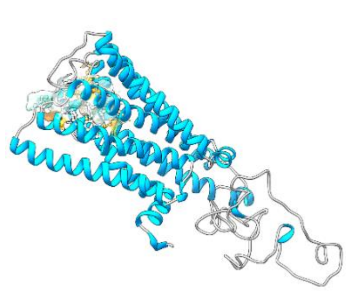	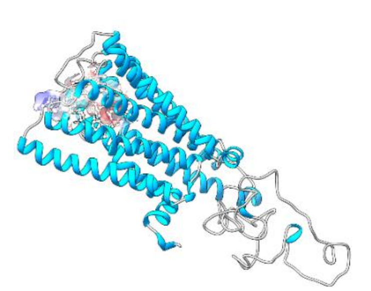	**CHAIN A**<br>ARG27; TYR91;<br>TYR94; VAL95;<br>TRP110; LEU111;<br>ASP114; TYR115;<br>CYS118; CYS188;<br>TYR189; ALA190;<br>PHE193; TYR374;<br>MET378; TYR394;<br>GLU395; PHE398<br>LEU401; TRP402	X: 13.77<br>Y: 16.95<br>Z: 9.16	X: -18.79<br>Y: 51.44<br>Z: 0.81
Ghrelin receptor-(ghrelinR)	7NA8	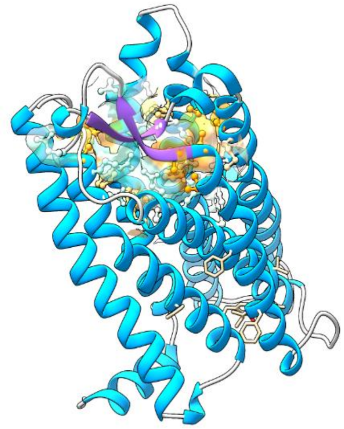	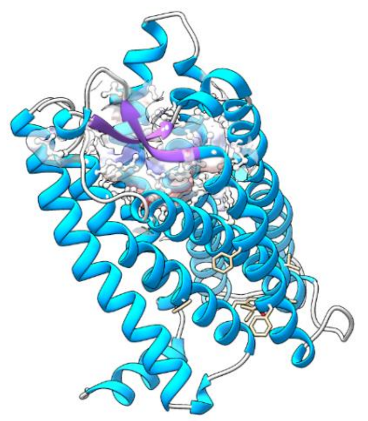	**CHAIN E [AUTH R]**<br>ASP99;ARG102;<br>LEU103;GLN105;<br>PHE119;GLN120;<br>SER123;GLU124;<br>TYR128;ILE178;<br>LEU181;VAL182;<br>PRO200;LEU210;<br>MET213;VAL214;<br>SER217;PHE279;<br>ARG283;PHE286;<br>SER287;SER301;<br>GLN302;ASN305;<br>LEU306;PHE309;<br>PHE312	X: 27.52<br>Y: 27.24<br>Z: 25.14	X: 125.52<br>Y: 137.06<br>Z: 95.38
GTPase KRas (KRAS)	7SCX	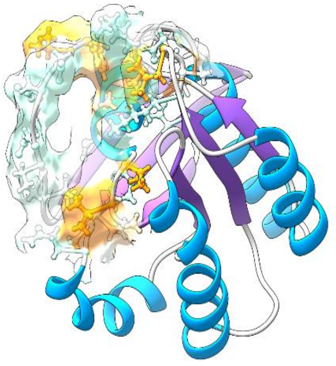	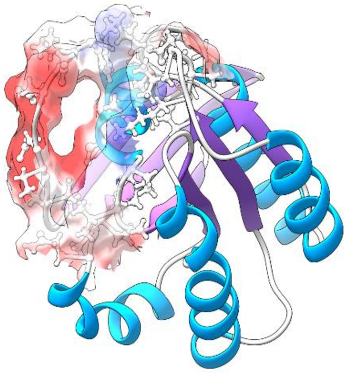	**CHAIN A**<br>ALA11;VAL12;<br>GLY13;VAL14;<br>GLY15;LYS16;<br>SER17;ALA18;<br>PHE28;VAL29;<br>ASP30;GLU31;<br>ASP33;PRO34;<br>THR35;THR58;<br>ALA59;GLY60;<br>GLN61;ASN116;<br>LYS117;SER118;<br>ASP119;LEU120;	X: 33.50<br>Y: 27.19<br>Z: 25.18	X: 13.52<br>Y: -8.64<br>Z: 25.47

				SER145;ALA146;LYS147		
Serotonin 6 receptor (5-HT6)	7XTB	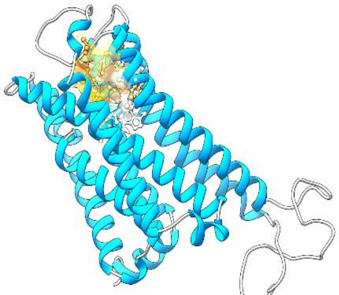	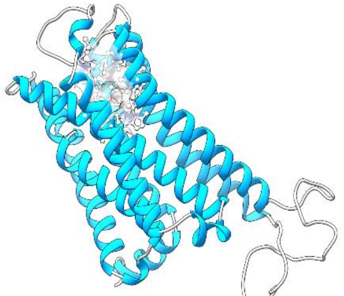	**CHAIN E** ASP106;VAL107; CYS110;SER111; LEU182;LEU183; PHE188;VAL189; ALA192;SER193; THR196;TRP281; PHE284;PHE285; ASN288;THR306; TYR310	X: 13.77 Y: 16.95 Z: 9.16	X: 85.41 Y: 109.95 Z: 82.19
Cathepsin L (CTSL)	8C77	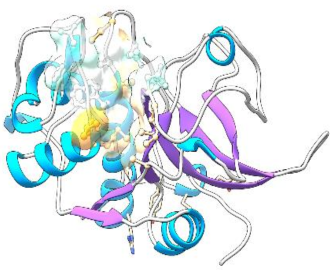	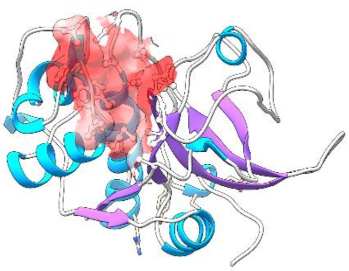	**CHAIN A** GLN19;CYS22; GLY23;SER24; CYS25;TRP26; GLU63;ASN66; GLY67;GLY68; LEU69;MET70; ALA135;MET161; ASP162;HIS163; GLY164;ALA214	X: 21.56 Y: 14.36 Z: 19.11	X: 16.53 Y: -21.60 Z: -0.97
Cytochrome P450 2C9 (CYP2C9),	1R9O	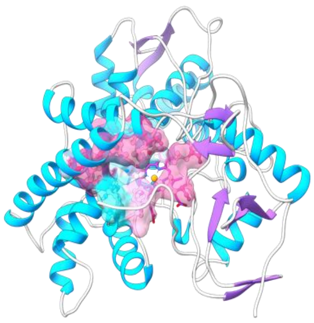	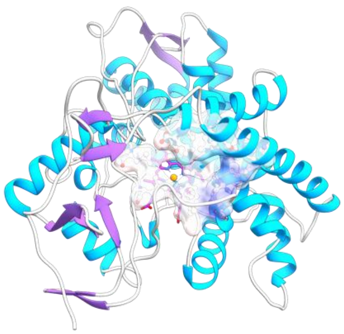	**CHAIN A** LEU87, ARG97, ILE112, VAL113, TRP120, ARG124, LEU131, ILE178, ASP293, LEU294, ALA297, GLY298, THR301, THR302, THR305, GLN356, LEU361, LEU362, SER365, LEU366, HIS368, LEU391, PRO427, PHE428, SER429, ALA430, ARG433, ILE434, CYS435, VAL436, GLY437, LEU440, ALA441, GLU444, LEU445	X: 20.47 Y: 18.83 Z: 17.69	X: 8.07 Y: 35.40 Z: -3.03

Cathepsin S (CTSS)	2FRA	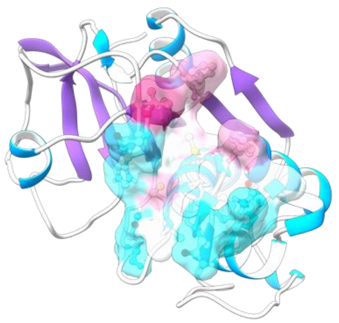	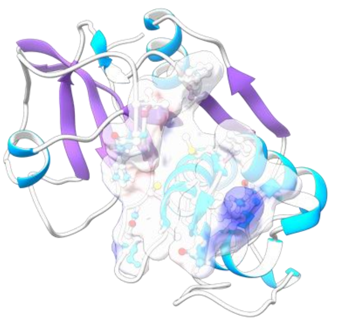	**CHAIN A** GLN19, GLY23, ALA24, CYS25, TRP26, GLY59, ASN60, LYS61, ASN64, GLY65, GLY66, PHE67, MET68, VAL132, GLY133, VAL157, ASN158, HIS159, GLY160, PHE205	X: 19.66 Y: 18.21 Z: 15.66	X: 51.26 Y: 21.23 Z: 125.59
Cyclin-dependent kinase 2/cyclin A (Cdk2/CA)	2R3F	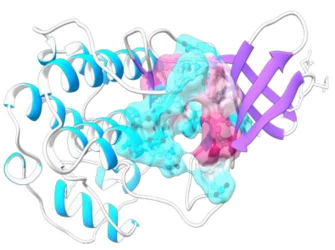	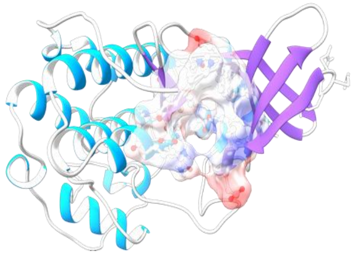	**CHAIN A** ILE10, GLY11, GLU12, GLY13, VAL18, ALA31, LYS33, VAL64, PHE80, GLU81, PHE82, LEU83, HIS84, GLN85, ASP86, LYS89, GLN131, ASN132, LEU134, ALA144, ASP145	X: 19.64 Y: 22.55 Z: 16.82	X: 1.53 Y: 28.87 Z: 9.785
Cyclin-dependent kinase 4 CDK4	2W9Z	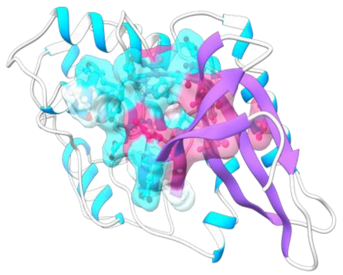	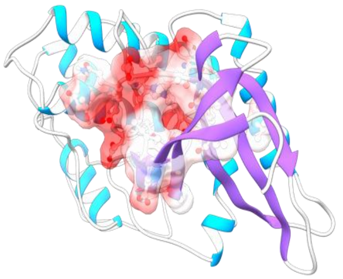	**CHAIN A** GLU11, ILE12, GLY13, GLY18, VAL20, VAL32, LEU34, VAL57, VAL72, PHE93, GLU94, HIS95, VAL96, ASP97, GLN98, ASP99, THR102, GLU144, ASN145, LEU147, ALA157, ASP158	X: 18.10 Y: 17.93 Z: 22.78	X: 20.08 Y: 25.65 Z: 11.26
Troponin, cardiac muscle (cTn)	4Y99	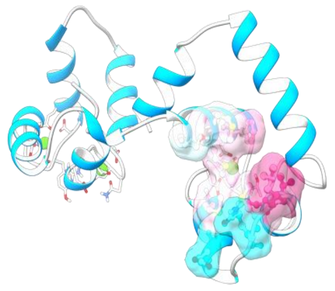	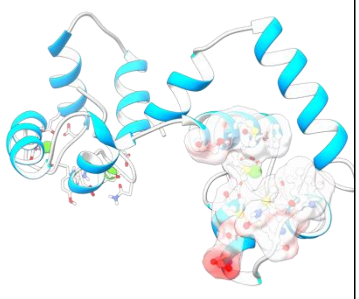	**CHAIN A** LEU41, VAL44, MET45, LEU48, GLN50, GLU56, MET60, PHE77, MET80, MET81, SER84	X: 17.03 Y: 14.93 Z: 20.53	X: 72.23 Y: 92.45 Z: 25.68

Mitogen-activated protein kinase kinase 8 (MAPK8)	5IU2	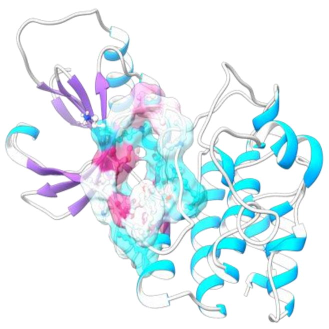	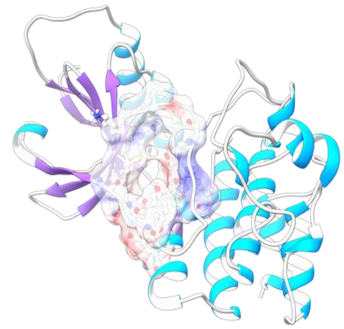	**CHAIN A** TRP132, LEU134, TYR136, PRO145, ALA148, PHE149, GLY150, LYS151, VAL152, ALA165, LYS167, MET207, GLU208, ALA209, GLY210, GLY213, SER214, GLU217, LYS255, SER257, ASN258, VAL260, VAL269, ASP270	X: 29.78<br>Y: 25.70<br>Z: 22.57	X: -43.77<br>Y: 1.45<br>Z: -11.26
Serine/threonine-protein kinase AKT (AKT1)	5KCV	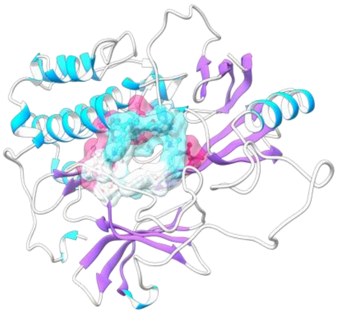	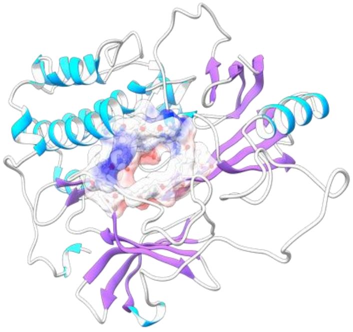	**CHAIN A** ASN53, ASN54, GLN79, TRP80, THR82, ILE84, SER205, LEU210, THR211, LEU264, LYS268, VAL270, VAL271, TYR272, ARG273, ASP274, ILE290, THR291, ASP292, GLY294	X: 23.98<br>Y: 19.84<br>Z: 23.83	X: -7.90<br>Y: -0.002<br>Z: -16.31
Insulin-degrading enzyme (IDE)	6EDS	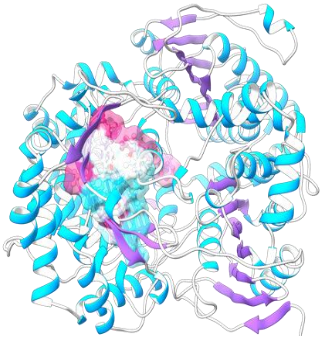	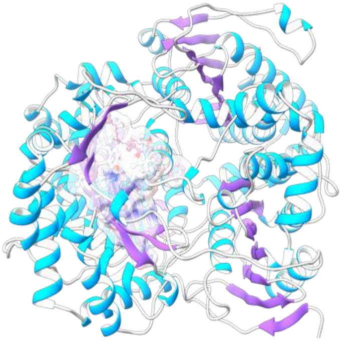	**CHAIN A** ALA198, LEU201, PHE202, LEU204, GLU205, THR208, TYR302, ILE304, TYR314, THR316, VAL360, GLY361, GLY362, GLN363, LYS364, ILE374, ASN376, ARG477, VAL478, ALA479	X: 24.96<br>Y: 24.78<br>Z: 28.49	X: 97.38<br>Y: -68.43<br>Z: -38.46
MAP kinase p38 alpha (p38α)	6SFI	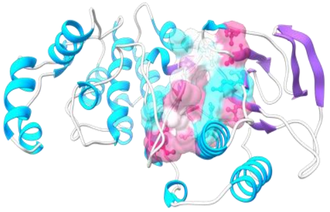	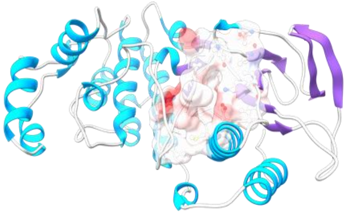	**CHAIN A** VAL30, TYR35, VAL38, ALA51, LYS53, GLU71, LEU74, LEU75, MET78, VAL83, ILE84, LEU104, THR106, HIS107, LEU108, MET109, GLY110, ALA111, ASP112, ILE141, ILE146, HIS148, ALA157, ILE166,	X: 18.98<br>Y: 20.78<br>Z: 18.22	X: 53.75<br>Y: 67.75<br>Z: 15.71

				LEU167, ASP168, PHE169, GLY170, LEU171, ALA172		
Cyclin-dependent kinase 2/cyclin E Cdk2/CE	7E34	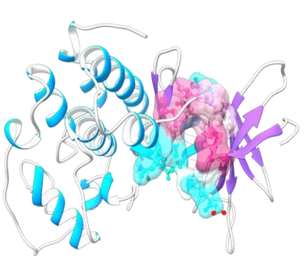	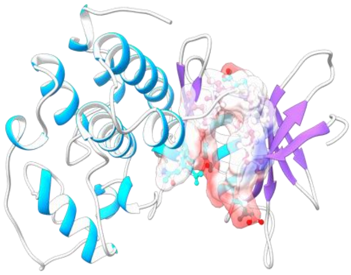	**CHAIN A** ILE10, GLY11, GLU12, GLY13, VAL18, ALA31, VAL64, PHE80, GLU81, PHE82, LEU83, GLN131, ASN132, LEU134, ALA144, ASP145	X: 13.91 Y: 17.85 Z: 18.32	X: -1.56 Y: -39.81 Z: 14.57
Cytochrome P450 3A4 CYP3A4	8EXB	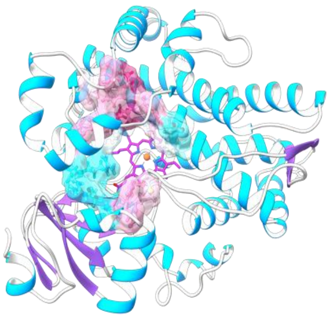	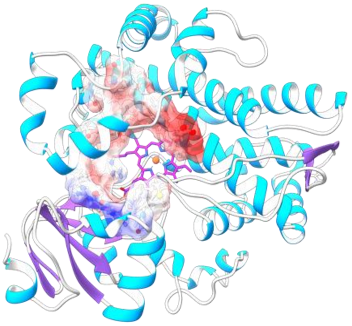	**CHAIN A** PHE57, ARG105, ARG106, PHE108, MET114, SER119, PHE241, ILE30, PHE301, ALA305, GLU308, THR309, ALA370, MET371, ARG372, GLU374	X: 22.06 Y: 26.52 Z: 30.24	X: -10.91 Y: -32.91 Z: -7.17

Table S3. Multiligand and Multitarget Docking Summary of **1a-k** with 20 Target Proteins. This table provides a summary of multiligand and multitarget docking results for compounds **1a-k** with 20 target proteins. Each ligand is color-coded, and within each ligand, each row corresponds to a calculated cluster.

**Protein**	**ID PDB**	**Ligand**	**Cluster Percentage**	**RMSD** (Å)	**Energy** (Kcal/mol)
DUSP3	3F81	**1a**	31.67%	1.015 ± 0.015	-6.840 ± 0.058
			26.67%	8.144 ± 0.005	-5.245 ± 0.135
		**1b**	33.33%	1.314 ± 0.022	-5.905 ± 0.127
			33.33%	4.761 ± 0.018	-6.114 ± 0.064
			33.33%	0.069 ± 0.049	-8.629 ± 0.039
		**1c**	33.33%	1.221 ± 0.028	-6.312 ± 0.084
			33.33%	4.773 ± 0.041	-6.314 ± 0.062
			31.67%	0.098 ± 0.064	-8.317 ± 0.034
		**1d**	48.33%	7.311 ± 0.027	-6.746 ± 0.193
			41.67%	7.801 ± 0.010	-5.793 ± 0.309
		**1e**	35.00%	0.925 ± 0.080	-6.833 ± 0.500
			30.00%	7.441 ± 0.100	-6.281 ± 0.056
		**1f**	65.00%	0.185 ± 0.105	-6.897 ± 0.122
			33.33%	3.712 ± 0.020	-5.125 ± 0.088
		**1g**	35.00%	8.339 ± 0.023	-6.003 ± 0.402
			33.33%	0.115 ± 0.048	-7.263 ± 0.021
			26.67%	6.210 ± 0.027	-6.419 ± 0.027
		**1h**	23.33%	0.171 ± 0.090	-7.850 ± 0.040
			33.33%	1.258 ± 0.019	-5.488 ± 0.157
			30.00%	7.326 ± 0.059	-6.967 ± 0.114
		**1i**	33.33%	5.194 ± 0.012	-6.238 ± 0.201
			31.67%	7.801 ± 0.050	-7.121 ± 0.048
		**1j**	31.67%	0.317 ± 0.084	-7.103 ± 0.056
			26.67%	8.204 ± 0.003	-6.056 ± 0.009
		**1k**	41.67%	3.378 ± 0.034	-7.099 ± 0.181
			35.00%	7.816 ± 0.067	-3.873 ± 2.879
MAPK3	3FHR	**1a**	33.33%	7.400 ± 0.006	-4.055 ± 0.051
			30.00%	4.589 ± 0.100	-7.170 ± 0.100
		**1b**	33.33%	8.163 ± 0.005	-5.474 ± 0.177
			33.33%	1.819 ± 0.059	-6.827 ± 0.030
			31.67%	0.111 ± 0.016	-7.114 ± 0.025
		**1c**	33.33%	1.819 ± 0.059	-6.430 ± 0.668

			33.33%	8.163 ± 0.005	-6.405 ± 0.659
			33.33%	0.111 ± 0.016	-6.319 ± 0.685
		**1d**	30.00%	7.178 ± 0.035	-6.117 ± 0.203
			28.33%	8.602 ± 0.041	-4.106 ± 0.359
		**1e**	33.33%	2.112 ± 0.039	-7.118 ± 0.025
			33.33%	7.208 ± 0.012	-2.807 ± 0.091
			28.33%	0.078 ± 0.077	-7.149 ± 0.012
		**1f**	33.33%	1.865 ± 0.011	-5.933 ± 0.008
			33.33%	0.062 ± 0.018	-6.943 ± 0.016
		**1g**	31.67%	3.346 ± 0.010	-5.593 ± 0.083
			31.67%	7.317 ± 0.012	-6.435 ± 0.014
			28.33%	8.012 ± 0.004	-6.232 ± 0.042
		**1h**	33.33%	0.066 ± 0.051	-7.222 ± 0.017
			33.33%	1.843 ± 1.163	-6.698 ± 0.348
		**1i**	33.33%	1.315 ± 0.006	-7.200 ± 0.044
			33.33%	0.088 ± 0.040	-8.546 ± 0.073
		**1j**	40.00%	8.413 ± 0.022	-6.747 ± 1.175
			33.33%	0.178 ± 0.024	-7.291 ± 0.043
		**1k**	33.33%	6.141 ± 0.008	-6.347 ± 0.169
			33.33%	0.045 ± 0.017	-7.935 ± 0.018
			31.67%	8.497 ± 0.002	-7.880 ± 0.022
5-HT6	7XTB	**1a**	33.33%	0.037 ± 0.013	-0.725 ± 0.022
			33.33%	8.492 ± 0.001	0.064 ± 0.033
			33.33%	8.578 ± 0.014	3.428 ± 0.128
		**1b**	66.67%	0.104 ± 0.075	-2.453 ± 0.219
			33.33%	0.906 ± 0.001	-0.303 ± 0.015
		**1c**	66.67%	0.220 ± 0.183	-2.593 ± 0.664
			33.33%	0.736 ± 0.003	-1.319 ± 0.056
		**1e**	66.67%	0.123 ± 0.092	0.093 ± 0.346
			33.33%	1.688 ± 0.005	0.348 ± 0.066
		**1g**	66.67%	0.313 ± 0.296	-2.302 ± 0.256
			33.33%	0.852 ± 0.003	-1.632 ± 0.094
H3	7F61	**1a**	66.67%	0.096 ± 3.930	-1.034 ± 0.117
		**1b**	100.00%	0.156 ± 0.090	-1.672 ± 0.154
		**1c**	63.33%	0.208 ± 0.160	-3.354 ± 0.512
			33.33%	4.235 ± 0.002	-0.148 ± 0.046
		**1e**	66.67%	0.104 ± 0.068	-1.930 ± 0.112

Protein	ID PDB	Ligand	Cluster Percentage	RMSD (Å)			Energy (Kcal/mol)		
KRAS	7SCX	1a	33.33%	0.066	±	0.023	-9.083	±	0.026
			33.33%	7.906	±	0.004	-8.106	±	0.012
			33.33%	8.869	±	0.006	-6.680	±	0.080
		1b	33.33%	0.060	±	0.028	-9.329	±	0.020
			33.33%	5.741	±	1.763	-7.370	±	0.998
		1c	33.33%	0.109	±	0.049	-9.106	±	0.060
			33.33%	7.738	±	0.008	-6.763	±	0.077
		1d	31.67%	0.076	±	0.027	-8.734	±	0.019
			33.33%	2.600	±	0.034	-8.069	±	0.010
		1e	33.33%	0.084	±	0.030	-9.542	±	0.017
			33.33%	6.789	±	0.004	-7.880	±	0.015
		1f	33.33%	0.037	±	0.019	-8.201	±	0.007
			33.33%	1.367	±	0.007	-8.159	±	0.009
			33.33%	9.258	±	0.053	-7.275	±	0.201
		1g	33.33%	0.113	±	0.051	-8.883	±	0.023
			33.33%	7.084	±	0.075	-3.451	±	0.658
		1h	33.33%	0.217	±	0.119	-8.959	±	0.034
			26.67%	1.229	±	0.112	-8.412	±	0.062
			25.00%	5.704	±	0.085	-4.936	±	1.538
		1i	33.33%	0.056	±	0.025	-9.698	±	0.015
			33.33%	8.161	±	0.007	-7.850	±	0.013
		1j	33.33%	0.139	±	0.043	-8.137	±	0.022
			33.33%	1.869	±	0.016	-7.915	±	0.004
			33.33%	7.855	±	0.027	-5.002	±	0.220
		1k	33.33%	0.066	±	0.024	-8.754	±	0.011
			33.33%	0.846	±	0.002	-8.643	±	0.005
			31.67%	7.335	±	0.508	-5.232	±	0.612
mGluR2	7EPF	1a	61.67%	0.257	±	0.194	-9.907	±	0.089
			33.33%	0.959	±	0.009	-9.078	±	0.040
		1b	66.67%	0.181	±	0.137	-9.616	±	0.022
			33.33%	3.618	±	0.014	-8.078	±	0.060
		1c	66.67%	0.190	±	0.156	-9.772	±	0.073
			33.33%	5.840	±	0.002	-6.951	±	0.032
		1d	33.33%	0.750	±	0.008	-9.769	±	0.021
			33.33%	1.895	±	0.003	-9.073	±	0.072
			33.33%	0.075	±	0.036	-9.912	±	0.031
		1e	66.67%	4.372	±	0.093	-9.632	±	0.073

			33.33%	0.028	±	0.011	-9.148	±	0.065
		1f	66.67%	0.252	±	0.187	-9.854	±	0.113
			33.33%	6.641	±	0.003	-7.667	±	0.048
		1g	33.33%	2.093	±	0.003	-9.249	±	0.005
			33.33%	0.045	±	0.017	-9.496	±	0.015
			33.33%	7.809	±	0.016	-8.116	±	0.262
		1h	63.33%	0.154	±	0.095	-10.911	±	0.144
			33.33%	1.576	±	0.005	-7.773	±	0.066
		1i	33.33%	0.048	±	0.013	-11.007	±	0.013
			33.33%	0.941	±	0.003	-10.630	±	0.006
			33.33%	2.210	±	0.008	-8.603	±	0.102
		1j	66.67%	0.165	±	0.051	-9.590	±	0.052
			33.33%	6.894	±	0.022	-7.691	±	0.137
		1k	33.33%	0.027	±	0.010	-10.766	±	0.008
			33.33%	0.898	±	0.005	-10.430	±	0.003
			33.33%	6.815	±	0.010	-8.274	±	0.147
CYP3A4	8EXB	1a	33.33%	11.441	±	0.030	-4.394	±	0.210
		1b	33.33%	3.400	±	0.026	-8.502	±	0.015
			33.33%	14.114	±	0.012	-5.751	±	0.134
		1c	63.33%	0.161	±	0.072	-8.818	±	0.068
		1d	33.33%	7.278	±	0.028	-4.545	±	0.220
			33.33%	11.036	±	0.009	-8.505	±	0.010
		1e	33.33%	0.213	±	0.077	-9.286	±	0.045
			33.33%	11.779	±	0.015	-8.204	±	0.009
		1f	33.33%	0.079	±	0.042	-9.339	±	0.026
		1g	33.33%	11.174	±	0.005	-8.100	±	0.052
		1h	33.33%	1.883	±	0.393	-9.484	±	0.182
		1i	33.33%	0.060	±	0.026	-10.209	±	0.021
			33.33%	12.226	±	0.001	-9.097	±	0.002
		1j	33.33%	10.396	±	0.023	-8.024	±	0.067
		1k	33.33%	0.096	±	0.041	-9.432	±	0.038
			33.33%	5.095	±	0.013	-5.720	±	0.085
			33.33%	10.671	±	0.011	-8.871	±	0.017
Cdk2/CE	7E34	1a	33.33%	0.047	±	0.019	-9.217	±	0.015
			33.33%	3.634	±	0.015	-7.755	±	0.010
			33.33%	7.780	±	0.007	-6.229	±	0.080
		1b	33.33%	0.062	±	0.018	-8.401	±	0.009
			33.33%	2.483	±	0.001	-8.083	±	0.013
		1c	33.33%	6.885	±	0.008	-7.532	±	0.073

			33.33%	8.494	±	0.001	-7.044	±	0.006
		1d	33.33%	0.078	±	0.029	-8.615	±	0.020
			33.33%	7.300	±	0.001	-7.937	±	0.006
			33.33%	8.431	±	0.010	-7.488	±	0.139
		1e	33.33%	0.103	±	0.029	-8.213	±	0.022
			33.33%	6.945	±	0.001	-7.053	±	0.003
			33.33%	7.131	±	0.015	-6.187	±	0.147
		1f	65.00%	0.054	±	0.018	-8.640	±	0.013
		1g	53.33%	7.758	±	0.195	-7.415	±	0.701
		1h	33.33%	8.600	±	0.007	-7.234	±	0.291
		1i	33.33%	0.059	±	0.019	-7.994	±	0.015
			33.33%	2.198	±	0.035	-7.463	±	0.024
			33.33%	9.619	±	0.003	-5.745	±	0.041
		1j	33.33%	5.747	±	0.011	-6.977	±	0.047
		1k	33.33%	0.064	±	0.026	-8.901	±	0.033
			33.33%	7.294	±	0.004	-8.620	±	0.017
			33.33%	7.965	±	0.010	-6.851	±	0.010
IDE	6EDS	1a	50.00%	11.756	±	0.070	-6.662	±	0.230
		1b	61.67%	0.121	±	0.067	-8.905	±	0.098
		1c	66.67%	0.137	±	0.096	-8.669	±	0.106
		1d	33.33%	0.069	±	0.021	-8.000	±	0.014
		1e	33.33%	0.048	±	0.023	-8.710	±	0.010
			33.33%	5.783	±	0.003	-7.610	±	0.011
		1f	33.33%	0.062	±	0.021	-7.860	±	0.014
			33.33%	12.276	±	0.004	-6.622	±	0.004
		1g	33.33%	0.095	±	0.035	-8.550	±	0.026
		1h	33.33%	0.274	±	0.070	-8.305	±	0.022
		1i	31.67%	8.030	±	0.008	-7.780	±	0.008
			31.67%	12.749	±	0.036	-4.619	±	0.468
		1j	31.67%	0.216	±	0.087	-8.750	±	0.053
		1k	56.67%	7.098	±	0.051	-7.160	±	1.442
AKT1	5KCV	1a	33.33%	0.071	±	0.030	-11.183	±	0.010
			33.33%	8.267	±	0.002	-9.999	±	0.006
			33.33%	8.813	±	0.008	-6.492	±	0.121
		1b	33.33%	0.007	±	0.005	-10.306	±	0.004
			33.33%	0.535	±	0.010	-10.132	±	0.009
		1c	48.33%	0.520	±	0.009	-10.244	±	0.013
		1d	66.67%	0.110	±	0.062	-11.240	±	0.032

		1e	33.33%	0.049	±	0.031	-10.610	±	0.009
			33.33%	5.422	±	0.001	-10.396	±	0.006
			33.33%	5.737	±	0.005	-7.074	±	0.005
		1f	66.67%	0.135	±	0.035	-10.938	±	0.024
		1g	33.33%	0.038	±	0.016	-10.823	±	0.013
			33.33%	5.941	±	0.006	-9.988	±	0.011
			33.33%	6.889	±	0.053	-5.885	±	0.272
		1h	33.33%	0.074	±	0.023	-11.930	±	0.020
		1i	60.00%	0.811	±	0.010	-10.717	±	0.044
		1j	33.33%	0.058	±	0.024	-10.342	±	0.020
			33.33%	5.886	±	0.028	-5.838	±	0.255
			33.33%	7.727	±	0.036	-9.648	±	0.079
		1k	66.67%	0.177	±	0.110	-11.185	±	0.047
cTn	4Y99	1a	35.00%	10.291	±	0.064	-5.744	±	0.090
		1b	33.33%	1.978	±	0.008	-4.920	±	0.005
			33..33%	9.893	±	0.005	-4.403	±	0.047
		1c	66.67%	6.175	±	0.047	-4.163	±	1.153
		1d	33.67%	8.611	±	0.010	-4.324	±	0.238
		1e	35.00%	8.044	±	0.069	-4.113	±	0.383
		1f	33.33%	0.046	±	0.019	-5.886	±	0.008
			33.33%	10.688	±	0.037	-4.681	±	0.122
			33.33%	11.127	±	0.085	-5.483	±	0.129
		1g	33.33%	0.089	±	0.030	-5.550	±	0.015
			33.33%	1.818	±	0.016	-5.138	±	0.010
		1h	41.67%	5.569	±	0.010	-4.155	±	0.148
		1i	33.33%	0.044	±	0.017	-6.097	±	0.011
			33.33%	7.434	±	0.004	-5.129	±	0.015
			33.33%	9.762	±	0.025	-3.594	±	0.064
		1j	90..00%	7.385	±	0.074	-5.350	±	0.448
		1k	58.33%	10.651	±	0.014	-4.992	±	0.753
CYP2C9	1R9O	1a	65.00%	0.085	±	0.068	-7.865	±	0.056
		1b	100.00%	0.201	±	0.176	-8.319	±	0.289
		1c	66.67%	0.089	±	0.057	-8.825	±	0.070
		1d	66.67%	0.080	±	0.034	-6.895	±	0.028
		1e	66.67%	0.127	±	0.123	-9.125	±	0.107
		1f	66.67%	6.690	±	0.058	-6.603	±	0.302
		1g	33.33%	6.905	±	0.002	-7.464	±	0.010
			33.33%	7.119	±	0.027	-6.651	±	0.250
		1h	66.67%	0.004	±	0.004	-8.642	±	0.010

		1i	33.33%	0.063	±	0.022	-9.677	±	0.030
			33.33%	7.920	±	0.001	-8.343	±	0.006
			33.33%	8.112	±	0.001	-7.509	±	0.017
		1j	33.33%	0.016	±	0.008	-7.579	±	0.012
			33.33%	5.695	±	0.047	-5.641	±	0.190
		1k	33.33%	0.015	±	0.009	-7.447	±	0.010
			33.33%	1.175	±	0.001	-7.197	±	0.007
			33.33%	8.746	±	0.001	-6.514	±	0.042
CTSS	2FRA	1a	33.33%	0.096	±	0.033	-7.859	±	0.038
			33.33%	5.444	±	0.013	-5.409	±	0.084
			33.33%	8.425	±	0.004	-7.358	±	0.009
		1b	33.33%	0.069	±	0.019	-8.127	±	0.019
			33.33%	1.089	±	0.007	-6.677	±	0.069
			33.33%	7.935	±	0.004	-7.747	±	0.007
		1c	35.00%	0.674	±	0.086	-6.788	±	0.442
		1d	33.33%	0.135	±	0.052	-7.675	±	0.040
			33.33%	6.899	±	0.010	-5.892	±	0.099
			33.33%	8.278	±	0.004	-7.103	±	0.008
		1e	33.33%	0.057	±	0.020	-8.057	±	0.025
			33.33%	1.299	±	0.005	-6.160	±	0.021
			33.33%	8.084	±	0.013	-7.017	±	0.048
		1f	33.33%	0.084	±	0.024	-7.625	±	0.033
			33.33%	6.806	±	0.012	-5.677	±	0.107
			33.33%	7.730	±	0.013	-7.471	±	0.045
		1g	33.33%	0.116	±	0.050	-8.290	±	0.046
			33.33%	7.687	±	0.015	-7.744	±	0.014
		1h	33.33%	8.296	±	0.061	-8.624	±	0.265
		1i	66.67%	0.251	±	0.203	-8.690	±	0.210
		1j	33.33%	0.046	±	0.016	-7.374	±	0.013
			33.33%	7.333	±	0.004	-6.747	±	0.011
			33.33%	8.601	±	0.009	-5.827	±	0.151
		1k	33.33%	0.065	±	0.030	-8.122	±	0.016
			33.33%	0.776	±	0.003	-7.904	±	0.019
			33.33%	1.371	±	0.008	-6.805	±	0.074
CDK4	2W9Z	1a	33.33%	0.041	±	0.018	-8.531	±	0.015
			33.33%	2.419	±	0.003	-7.834	±	0.010
			33.33%	8.323	±	0.001	-6.891	±	0.102
		1b	33.33%	0.088	±	0.030	-8.295	±	0.025
			33.33%	2.049	±	0.007	-8.163	±	0.015

			33.33%	8.048	±	0.005	-6.055	±	0.069
		**1c**	33.33%	1.327	±	0.015	-8.266	±	0.020
			33.33%	9.171	±	0.016	-6.337	±	0.063
		**1d**	33.33%	1.144	±	0.051	-8.083	±	0.015
		**1e**	33.33%	0.072	±	0.031	-8.338	±	0.026
			33.33%	9.458	±	2.703	-5.633	±	0.125
		**1f**	33.33%	0.097	±	0.090	-8.201	±	0.022
			33.33%	0.571	±	0.031	-8.141	±	0.009
			33.33%	3.658	±	0.006	-4.073	±	0.110
		**1g**	33.33%	0.275	±	0.069	-7.483	±	0.145
			33.33%	5.416	±	0.042	-5.589	±	0.237
		**1h**	33.33%	0.146	±	0.057	-8.535	±	0.126
		**1i**	33.33%	0.710	±	0.003	-8.548	±	0.003
		**1j**	33.33%	0.055	±	0.022	-8.517	±	0.026
			33.33%	8.700	±	0.004	-7.203	±	0.014
		**1k**	33.33%	0.065	±	0.050	-8.561	±	0.016
			33.33%	0.871	±	0.001	-8.230	±	0.004
Ox2R	6TPN	**1a**	66.67%	0.251	±	0.128	-9.764	±	0.141
		**1b**	66.67%	0.208	±	0.119	-9.865	±	0.182
		**1c**	66.67%	0.164	±	0.115	-9.830	±	0.157
			28.33%	5.138	±	1.016	-6.785	±	0.433
		**1d**	66.67%	0.236	±	0.218	-7.869	±	1.755
			31.67%	8.544	±	0.021	-6.185	±	0.105
		**1e**	66.67%	0.175	±	0.128	-9.558	±	0.206
			31.67%	11.561	±	0.012	-6.172	±	0.112
		**1f**	33.33%	0.865	±	0.001	-9.428	±	0.007
			33.33%	0.090	±	0.032	-9.933	±	0.026
			31.67%	9.105	±	0.004	-5.534	±	0.021
		**1g**	33.33%	0.787	±	0.004	-8.880	±	0.017
			33.33%	0.088	±	0.043	-9.021	±	0.032
			26.67%	11.292	±	0.118	-4.285	±	0.340
		**1h**	33.33%	0.057	±	0.023	-10.154	±	0.015
			33.33%	1.173	±	0.013	-9.354	±	0.015
			33.33%	4.436	±	0.017	-7.449	±	0.263
		**1i**	33.33%	0.840	±	0.001	-9.904	±	0.009
			33.33%	0.052	±	0.020	-10.110	±	0.014
			31.67%	8.633	±	0.005	-7.506	±	0.044
		**1j**	66.67%	0.246	±	0.159	-9.655	±	0.168
			28.33%	11.345	±	0.093	-7.111	±	0.345

		**1k**	66.67%	0.257	±	0.200	-10.590	±	0.245
			31.67%	9.110	±	0.012	-5.953	±	0.129
ghrelinR	7NA8	**1a**	33.33%	7.955	±	0.003	-6.926	±	0.039
			33.33%	0.060	±	0.018	-10.569	±	0.016
			33.33%	2.625	±	0.004	-9.383	±	0.008
		**1b**	33.33%	0.036	±	0.016	-9.874	±	0.012
			33.33%	5.439	±	0.001	-9.190	±	0.003
			33.33%	6.194	±	0.006	-8.147	±	0.074
		**1c**	66.67%	0.239	±	0.184	-9.740	±	0.109
			33.33%	9.159	±	0.072	-6.542	±	0.529
		**1d**	33.33%	7.561	±	0.005	-7.753	±	0.060
			33.33%	0.088	±	0.026	-10.394	±	0.020
			33.33%	9.010	±	0.001	-9.683	±	0.005
		**1e**	33.33%	9.432	±	0.007	-6.236	±	0.119
			33.33%	0.058	±	0.021	-9.700	±	0.023
			33.33%	8.803	±	0.002	-8.980	±	0.006
		**1f**	66.67%	0.183	±	0.101	-9.589	±	0.128
			33.33%	8.695	±	0.010	-7.588	±	0.078
		**1g**	66.67%	0.119	±	0.071	-9.471	±	0.113
			31.67%	4.882	±	0.061	-7.652	±	0.163
		**1h**	66.67%	0.208	±	0.123	-9.917	±	0.172
		**1i**	33.33%	11.375	±	0.007	-8.477	±	0.036
			33.33%	0.067	±	0.029	-10.711	±	0.034
			33.33%	8.672	±	0.002	-9.767	±	0.006
		**1j**	66.67%	0.131	±	0.070	-9.929	±	0.419
		**1k**	33.33%	7.546	±	0.006	-8.342	±	0.038
			33.33%	0.047	±	0.019	-10.913	±	0.015
			33.33%	9.272	±	0.001	-10.050	±	0.006
Cdk2/CA	2R3F	**1a**	38.33%	0.035	±	0.020	-7.660	±	0.009
		**1b**	66.67%	0.131	±	0.058	-8.180	±	0.083
		**1c**	66.67%	0.152	±	0.092	-8.170	±	0.119
		**1d**	33.33%	0.093	±	0.027	-6.680	±	0.010
			33.33%	7.373	±	0.002	-5.272	±	0.015
		**1e**	33.33%	0.049	±	0.022	-8.060	±	0.025
			33.33%	7.589	±	0.037	-6.160	±	0.021
			33.33%	9.074	±	0.007	-7.017	±	0.048
		**1f**	61.67%	7.302	±	0.191	6.538	±	22.900
		**1g**	66.67%	7.676	±	0.131	-6.270	±	0.404
		**1h**	66.67%	7.661	±	0.147	-6.031	±	0.339

		**1i**	33.33%	0.028	±	0.009	-8.000	±	0.014
			33.33%	9.401	±	0.006	-5.229	±	0.105
		**1j**	33.33%	0.067	±	0.026	-6.915	±	0.038
			33.33%	10.191	±	0.038	-5.512	±	0.207
		**1k**	33.33%	0.026	±	0.010	-7.133	±	0.010
p38α	6SF1	**1a**	66.67%	1.091	±	0.007	-7.122	±	0.145
		**1b**	66.67%	0.196	±	0.155	-7.825	±	0.169
		**1c**	66.67%	0.176	±	0.104	-7.397	±	0.125
		**1d**	50.00%	0.779	±	0.139	-5.644	±	0.635
		**1e**	66.67%	0.173	±	0.125	-7.351	±	0.206
		**1f**	35.00%	0.763	±	0.021	-5.520	±	0.148
		**1g**	61.67%	0.724	±	0.231	-7.530	±	0.203
		**1h**	66.67%	0.208	±	0.153	-7.753	±	0.269
		**1i**	40.00%	0.930	±	0.018	-6.352	±	0.728
		**1j**	33.33%	0.030	±	0.009	-6.871	±	0.028
			33.33%	0.910	±	0.006	-6.157	±	0.098
			33.33%	8.927	±	0.010	-5.235	±	0.111
		**1k**	33.33%	0.050	±	0.017	-8.146	±	0.049
			33.33%	0.989	±	0.005	-6.410	±	0.053
			33.33%	8.960	±	0.004	-5.878	±	0.014
MAPK8	5IU2	**1a**	33.33%	0.052	±	0.020	-9.490	±	0.013
		**1b**	33.33%	0.060	±	0.017	-9.610	±	0.013
			33.33%	1.706	±	0.002	-9.206	±	0.005
			33.33%	2.157	±	0.003	-8.840	±	0.115
		**1c**	33.33%	0.066	±	0.019	-9.370	±	0.035
			33.33%	2.044	±	0.008	-7.963	±	0.038
		**1d**	33.33%	0.055	±	0.022	-9.442	±	0.014
			33.33%	7.782	±	0.007	-6.128	±	0.058
		**1e**	33.33%	0.099	±	0.038	-9.822	±	0.037
			33.33%	1.022	±	0.001	-9.560	±	0.008
			33.33%	2.833	±	0.024	-5.766	±	0.145
		**1f**	35.00%	7.810	±	0.023	-6.073	±	0.236
		**1g**	33.33%	0.043	±	0.020	-9.216	±	0.016
			33.33%	5.202	±	0.010	-7.676	±	0.082
			33.33%	11.167	±	0.004	-5.219	±	0.010
		**1h**	58.00%	0.080	±	0.055	-9.228	±	0.091
		**1i**	55.00%	0.086	±	0.045	-9.648	±	1.443
		**1j**	38.33%	7.605	±	0.095	-5.640	±	0.467
		**1k**	65.00%	1.533	±	0.249	-9.750	±	0.072

Protein	ID PDB	Ligand	Cluster Percentage	RMSD (Å)	Energy (Kcal/mol)
CTSL	8C77	**1a**	31.67%	0.038 ± 0.015	-7.101 ± 0.008
			33.33%	4.376 ± 0.004	-3.585 ± 0.068
			33.33%	5.697 ± 0.022	-5.715 ± 0.019
		**1b**	33.33%	0.067 ± 0.023	-6.674 ± 0.020
			33.33%	8.469 ± 0.004	-5.320 ± 0.009
			30.00%	9.580 ± 0.058	-4.249 ± 0.579
		**1c**	33.33%	0.105 ± 0.034	-7.042 ± 0.021
			30.00%	3.995 ± 0.003	-5.666 ± 0.004
			33.33%	9.771 ± 0.042	-5.006 ± 0.175
		**1d**	66.67%	0.181 ± 0.081	-6.444 ± 0.082
			26.67%	8.125 ± 0.006	-3.153 ± 0.097
		**1e**	33.33%	0.054 ± 0.020	-6.759 ± 0.009
		**1f**	33.33%	0.109 ± 0.102	-6.521 ± 0.076
			33.33%	1.165 ± 0.013	-6.320 ± 0.009
			33.33%	5.285 ± 0.060	-4.216 ± 0.357
		**1g**	33.33%	1.678 ± 0.116	-2.654 ± 0.213
			33.33%	6.086 ± 0.019	-5.367 ± 0.010
		**1h**	30.00%	7.435 ± 0.013	-4.927 ± 0.187
			33.33%	10.265 ± 0.028	-5.904 ± 0.012
		**1i**	31.67%	1.634 ± 0.021	-7.342 ± 0.031
			33.33%	7.967 ± 0.000	-5.916 ± 0.002
			33.33%	10.072 ± 0.006	-4.726 ± 0.115
		**1j**	33.33%	1.236 ± 0.087	-4.931 ± 0.873
			33.33%	7.578 ± 0.003	-5.802 ± 0.006
		**1k**	33.33%	0.067 ± 0.024	-6.583 ± 0.014
			33.33%	5.292 ± 0.165	-5.407 ± 0.910

Table S4. Key ADMETox Predicted Properties of the compounds **1a-k**. This table summarizes the most relevant ADMETox (Absorption, Distribution, Metabolism, Excretion, and Toxicity) predicted properties for the compounds **1a-k**.

	**Parameters**	**1a**	**1b**	**1c**	**1d**	**1e**	**1f**	**1g**	**1h**	**1i**	**1j**	**1k**
**Properties**	Molecular weight (g/mol)	455.5	459.54	459.54	455.51	469.54	487.64	463.57	475.58	475.58	463.57	461.56
	Heavy atoms	34	34	34	34	34	34	35	36	36	35	35
	Arom. heavy atoms	22	17	17	22	17	33	24	24	24	24	24
	Rotatable bonds	6	6	6	6	6	6	8	6	6	8	6
	H-bond acceptors	5	5	5	5	5	3	4	4	4	4	4
	H-bond donors	0	0	0	0	0	0	0	0	0	0	0
	Molar Refractivity	132.78	134.23	134.23	132.78	134.23	144	141.93	147.56	147.56	141.93	142.75
	TPSA (Å)	71.95	68.04	68.04	71.95	68.04	102.15	62.22	62.22	62.22	62.22	62.22
Lipophilicity	Log Po/w	3.98	4.04	4.01	3.92	3.87	4.46	3.78	3.83	3.87	3.79	3.6
Water solubility	Log S (ESOL)	-4.65	-4.45	-4.42	-4.62	-4.34	-5.62	-5.07	-5.34	-5.36	-5.1	-5.06
Pharmacokinetics	GI absorption	High	High	High	High	High	High	High	High	High	High	High
	BBB	Yes	Yes	Yes	Yes	Yes	No	Yes	Yes	Yes	Yes	Yes
	CYP1A2 inhibitor	No	No	No	No	No	No	Yes	No	No	Yes	Yes
	CYP2C19 inhibitor	No	No	No	No	Yes	No	Yes	No	No	Yes	No
	CYP2C9 inhibito	Yes	Yes	Yes	Yes	Yes	Yes	Yes	Yes	Yes	Yes	Yes
	CYP2D6	Yes	Yes	Yes	Yes	Yes	Yes	Yes	Yes	Yes	Yes	Yes
	CYP3A4 inhibitor	Yes	Yes	Yes	Yes	Yes	Yes	Yes	Yes	Yes	Yes	Yes
Drug likeness	Lipinski, Violation	Yes, 0	Yes, 0	Yes, 0	Yes, 0	Yes, 0	Yes, 0	Yes, 0	Yes, 0	Yes, 0	Yes, 0	Yes, 0
	Bioavailability Score	0.55	0.55	0.55	0.55	0.55	0.55	0.55	0.55	0.55	0.55	0.55
Medicinal Chemistry	Synthetic accessibility	4.24	4.62	4.6	4.28	4.6	4.18	4.2	4.18	4.19	4.17	4.1
Organ toxicity	Hepatotoxicity	Inactive	Inactive	Inactive	Inactive	Inactive	Inactive	Inactive	Inactive	Inactive	Inactive	Inactive
Toxicity end points	Carcinogenicity	Inactive	Inactive	Inactive	Inactive	Inactive	Inactive	Inactive	Inactive	Inactive	Inactive	Inactive
	Immunotoxicity	Inactive	Inactive	Inactive	Inactive	Inactive	Inactive	Inactive	Inactive	Inactive	Active	Inactive
	Mutagenicity	Inactive	Inactive	Inactive	Inactive	Inactive	Inactive	Inactive	Inactive	Inactive	Inactive	Inactive
	Cytotoxicity	Inactive	Inactive	Inactive	Inactive	Inactive	Inactive	Inactive	Inactive	Inactive	Inactive	Inactive

Tox21-Nuclear receptor signalling pathways	Androgen Receptor (AR)	Inactive	Inactive	Inactive	Inactive	Inactive	Inactive	Inactive	Inactive	Inactive	Inactive	Inactive
	Aryl hydrocarbon Receptor (AhR)	Inactive	Inactive	Inactive	Inactive	Inactive	Inactive	Inactive	Inactive	Inactive	Inactive	Inactive
	Estrogen Receptor Alpha (ER)	Inactive	Inactive	Inactive	Inactive	Inactive	Inactive	Inactive	Inactive	Inactive	Inactive	Inactive
	Peroxisome Proliferator Activated Receptor Gamma (PPAR-Gamma)	Inactive	Inactive	Inactive	Inactive	Inactive	Inactive	Inactive	Inactive	Inactive	Inactive	Inactive
Tox21-Stress response pathways	Heat shock factor response element (HSE)	Inactive	Inactive	Inactive	Inactive	Inactive	Inactive	Inactive	Inactive	Inactive	Inactive	Inactive
	Mitochondrial Membrane Potential (MMP)	Inactive	Inactive	Inactive	Inactive	Inactive	Inactive	Inactive	Inactive	Inactive	Inactive	Inactive
	Phosphoprotein (Tumor Supressor) p53	Inactive	Inactive	Inactive	Inactive	Inactive	Inactive	Inactive	Inactive	Inactive	Inactive	Inactive
	ATPase family AAA domain-containing protein 5 (ATAD5)	Inactive	Inactive	Inactive	Inactive	Inactive	Inactive	Inactive	Inactive	Inactive	Inactive	Inactive
**Predicted LD_{50} (mg/kg)**		**1600**	**1600**	**1600**	**1600**	**1600**	**1500**	**4000**	**110**	**110**	**2680**	**110**
Toxicity class		4	4	4	4	4	4	5	3	3	5	3

Table S5. Structural Time Equilibrium Descriptors. This table displays the average and standard deviation of the RMSD (Root Mean Square Deviation) for the protein, ligand, and radius of gyration for each of the replicas during 100 ns of simulation.

Target protein	Ligand	Replica	RMSD-Protein (Å)	RMSD Ligand (Å)	Rgyr (Å)
AKT1	**1f**	R1	2.4025 ± 0.2871	1.439 ± 0.289	21.9623 ± 0.1803
		R2	2.3412 ± 0.4007	1.5994 ± 0.1909	21.9238 ± 0.1099
		R3	2.0905 ± 0.2647	1.7344 ± 0.3844	21.8005 ± 0.1432
	**1h**	R1	2.8726 ± 0.3802	2.3619 ± 0.3555	21.8517 ± 0.1034
		R2	1.9754 ± 0.2241	2.5027 ± 0.2909	21.6567 ± 0.0969
		R3	2.1812 ± 0.2204	2.3952 ± 0.2048	21.8968 ± 0.1655
	**1k**	R1	2.4025 ± 0.2871	1.741 ± 0.259	21.9125 ± 0.1157
		R2	2.3412 ± 0.4007	1.3488 ± 0.3475	22.0108 ± 0.1306
		R3	2.0905 ± 0.2647	1.6949 ± 0.3008	21.7311 ± 0.0842
Ox2R	**1f**	R1	1.9067 ± 0.2909	2.423 ± 0.4735	22.0161 ± 0.1274
		R2	1.543 ± 0.1472	0.8904 ± 0.1294	21.8883 ± 0.0684
		R3	2.3756 ± 0.224	0.7163 ± 0.117	22.1307 ± 0.1128
	**1h**	R1	1.8107 ± 0.21	1.8773 ± 0.1452	21.676 ± 0.0824
		R2	1.8083 ± 0.2014	2.0377 ± 0.153	21.8907 ± 0.0888
		R3	2.2593 ± 0.2931	2.0817 ± 0.1868	21.913 ± 0.1278
	**1k**	R1	1.7244 ± 0.1655	1.4069 ± 0.3416	21.7798 ± 0.0976
		R2	1.6152 ± 0.195	1.2641 ± 0.3001	21.7052 ± 0.096
		R3	1.7094 ± 0.2523	1.4441 ± 0.5174	21.7755 ± 0.1255

Table S6. Decomposition of Key Amino Acids for Interaction at 5 Å Calculated Using MM/GBSA.

Target protein							
AKT_1				Ox_2R			
Aminoacid	Inhibitor			Aminoacid	Inhibitor		
	**1f**	**1h**	**1k**		**1f**	**1h**	**1k**
ASN67	-1.1 ± 1.12	-1.06 ± 0.79	-	VAL78	-1.56 ± 0.31	-	-1.53 ± 0.45
ASN68	-1.32 ± 0.86	-	-0.52 ± 0.25	THR83	-1.23 ± 0.36	-	-
GLN93	-2.69 ± 1.26	-1.22 ± 1.23	-1.12 ±0.65	ILE94	-0.76 ± 0.3	-0.6 ± 0.22	-1.04 ± 0.28
TRP94	-3.64 ± 1.29	-2.6 ± 0.86	-3.67 ± 0.7	PRO95	-0.76 ± 0.32	-0.92 ± 0.36	-1.38 ± 0.42
THR95	-	-	-0.59 ± 0.2	GLN98	-	-0.68 ± 0.59	-0.58 ± 0.32
THR96	-	-	-1.2 ± 0.38	THR99	-	-0.69 ± 0.5	-
GLN217	-0.58 ± 0.59	-	-	MET155	-0.8 ± 0.34	-1.0 ± 0.39	-0.9 ± 0.37
LEU224	-	-1.31 ± 0.57	-1.47 ± 0.37	VAL165	-1.24 ± 0.47	-	-1.03 ± 0.36
THR225	-	-	-0.57 ± 0.23	CYS166	-	-	-0.59 ± 0.45
TYR277	-	-	-0.57 ± 0.24	ASP167	0.84 ± 0.55	0.63 ± 0.44	1.55 ± 0.74
LEU278	-0.66 ± 0.24	-0.63 ± 0.27	-1.02 ± 0.29	GLU168	-	1.07 ± 1.08	-
LYS282	-0.81 ± 0.5	-1.07 ± 0.6	-0.94 ± 0.62	LYS252	0.52 ± 0.46	-0.91 ± 1.07	-
VAL284	-2.02 ± 0.29	-1.48 ± 0.56	-2.11 ± 0.29	PHE258	-	-0.5 ± 0.31	-
TYR286	-1.08 ± 0.36	-0.63 ± 0.31	-1.43 ± 0.31	ARG264	-0.55 ± 0.46	-	-
ASP288			0.63 ± 0.18	VAL267	-0.52 ± 0.27	-0.53 ± 0.2	-0.71 ± 0.27
ILE304			-0.63 ± 0.28	TYR268	-1.79 ± 0.65	-1.65 ± 1.18	-1.57 ± 0.64
THR305			-1.08 ± 0.33	PHE271	-1.17 ± 0.55	-2.44 ± 0.51	-1.63 ± 0.57
ASP306		0.74 ± 0.85	1.26 ± 0.63	HSP275		-2.19 ± 1.82	
Ligand	-3.98 ± 0.57	-8.37 ± 0.16	-11.77 ± 0.06	Ligand	-2.68 ± 0.21	-11.57 ± 0.81	-10.3 ± 0.85

Table S7. 2D and 3D Representations of Optimized Structures for ligands **1a-k**.

Ligand	2D Image	3D Image
**1a**	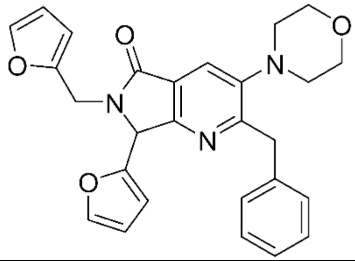	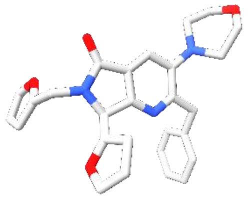
**1b**	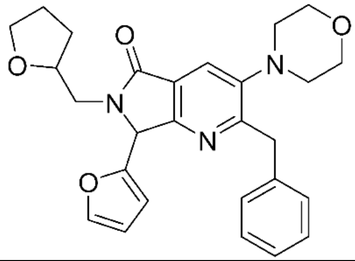	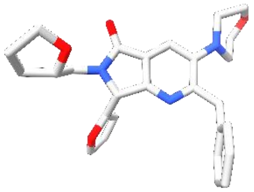
**1c**	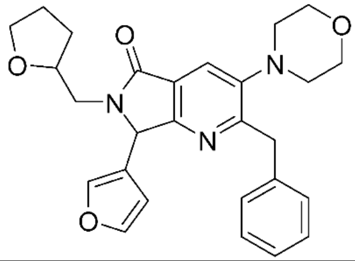	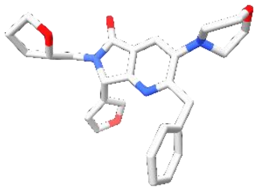
**1d**	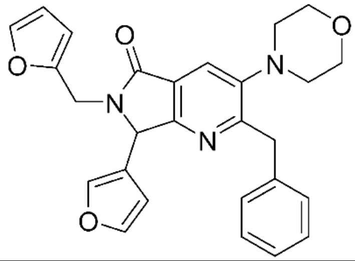	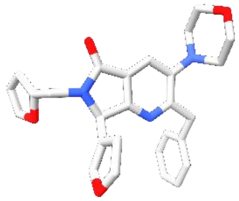
**1e**	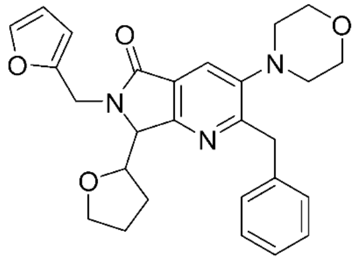	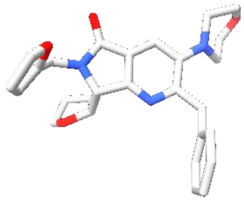
**1f**	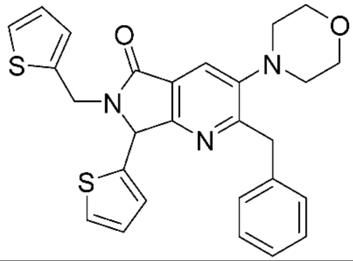	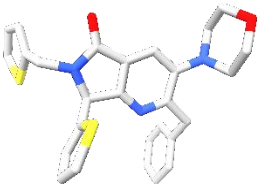

**1g**	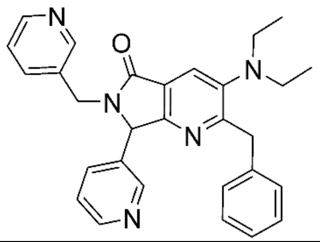	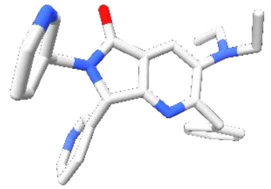
**1h**	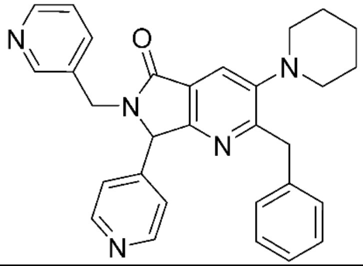	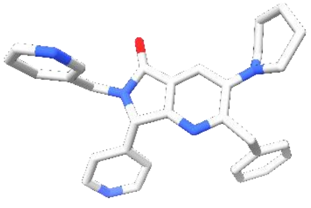
**1i**	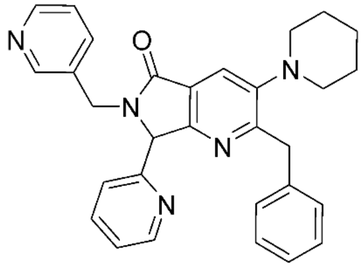	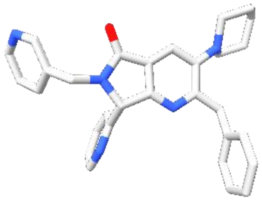
**1j**	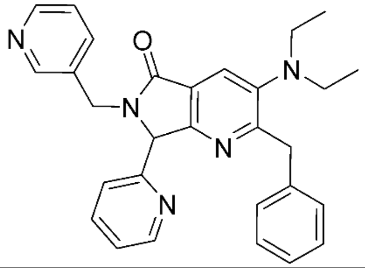	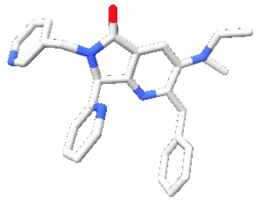
**1k**	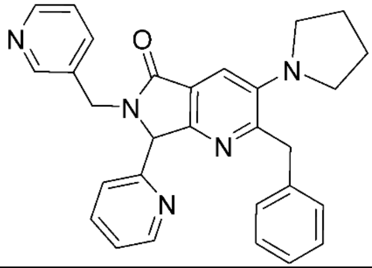	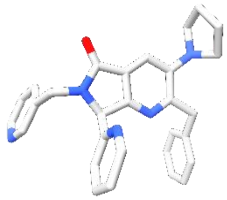

Table S8. Target Proteins. Ribbon representation of the 20 potential target proteins. Modeled sections for each protein are highlighted in orange.

PDB-D	Image
3FHR	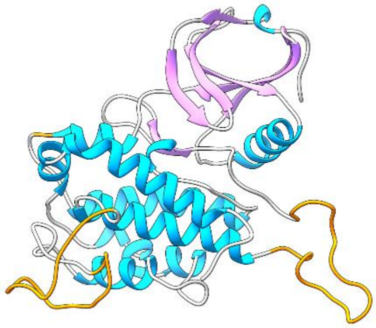
6TPN	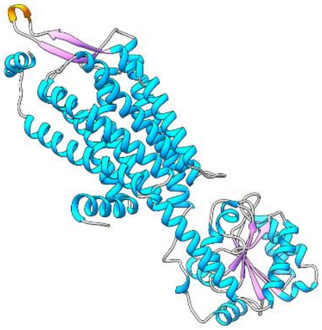
7EPF	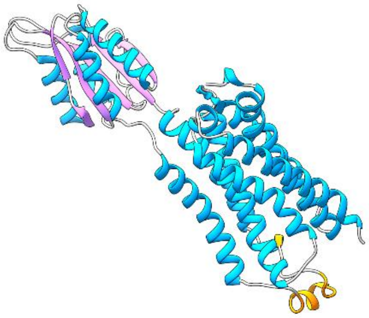
7F61	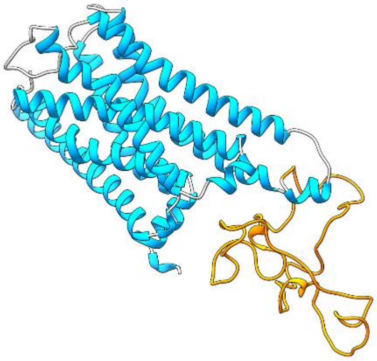

7NA8	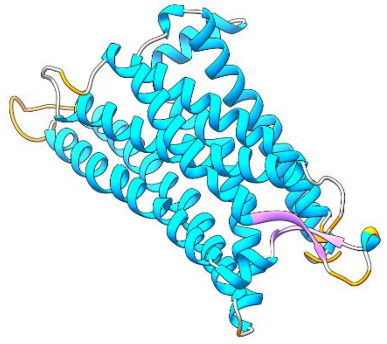
7XTB	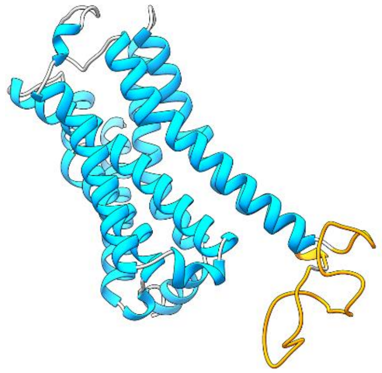
1R9O	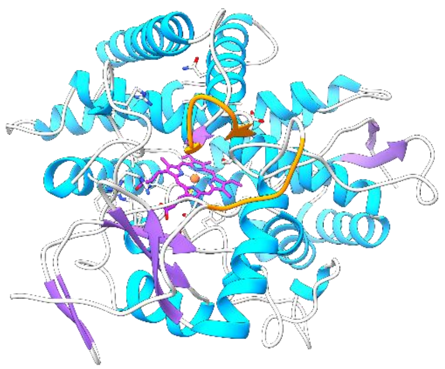
4Y99	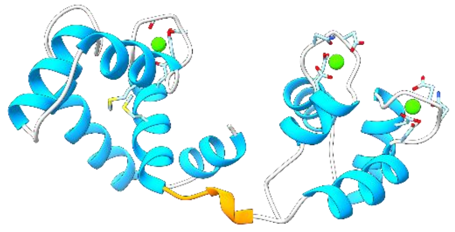

5IU2	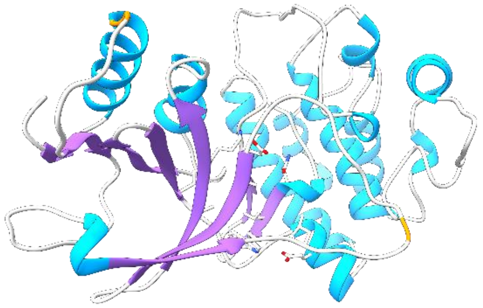
5KCV	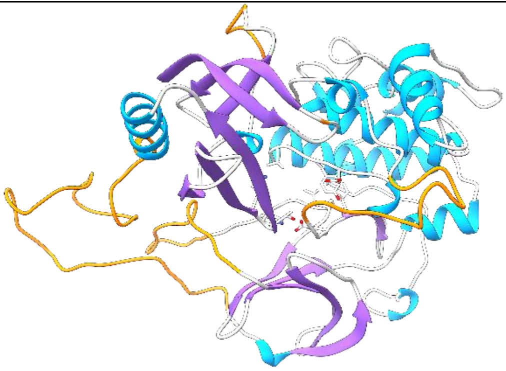
6EDS	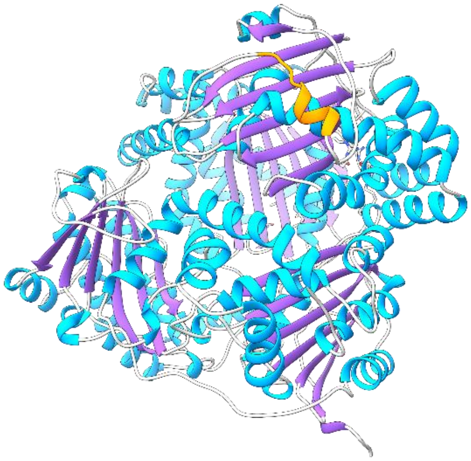
6SFI	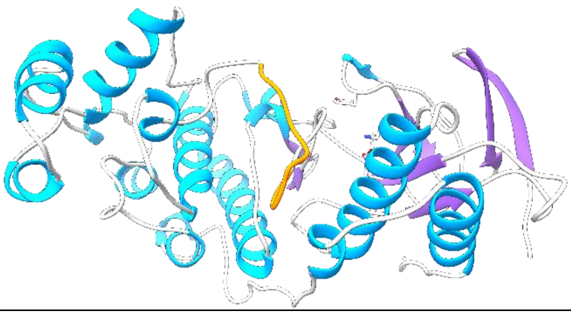

8EXB	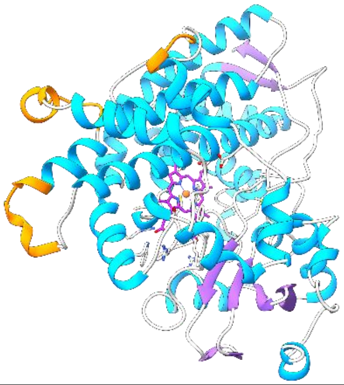

Figure S24. Structure of Protein AKT1. The cyan color represents the Pleckstrin Homology domain, yellow indicates the linking loop, and green represents the kinase domain. The red color represents the surface for the allosteric site.

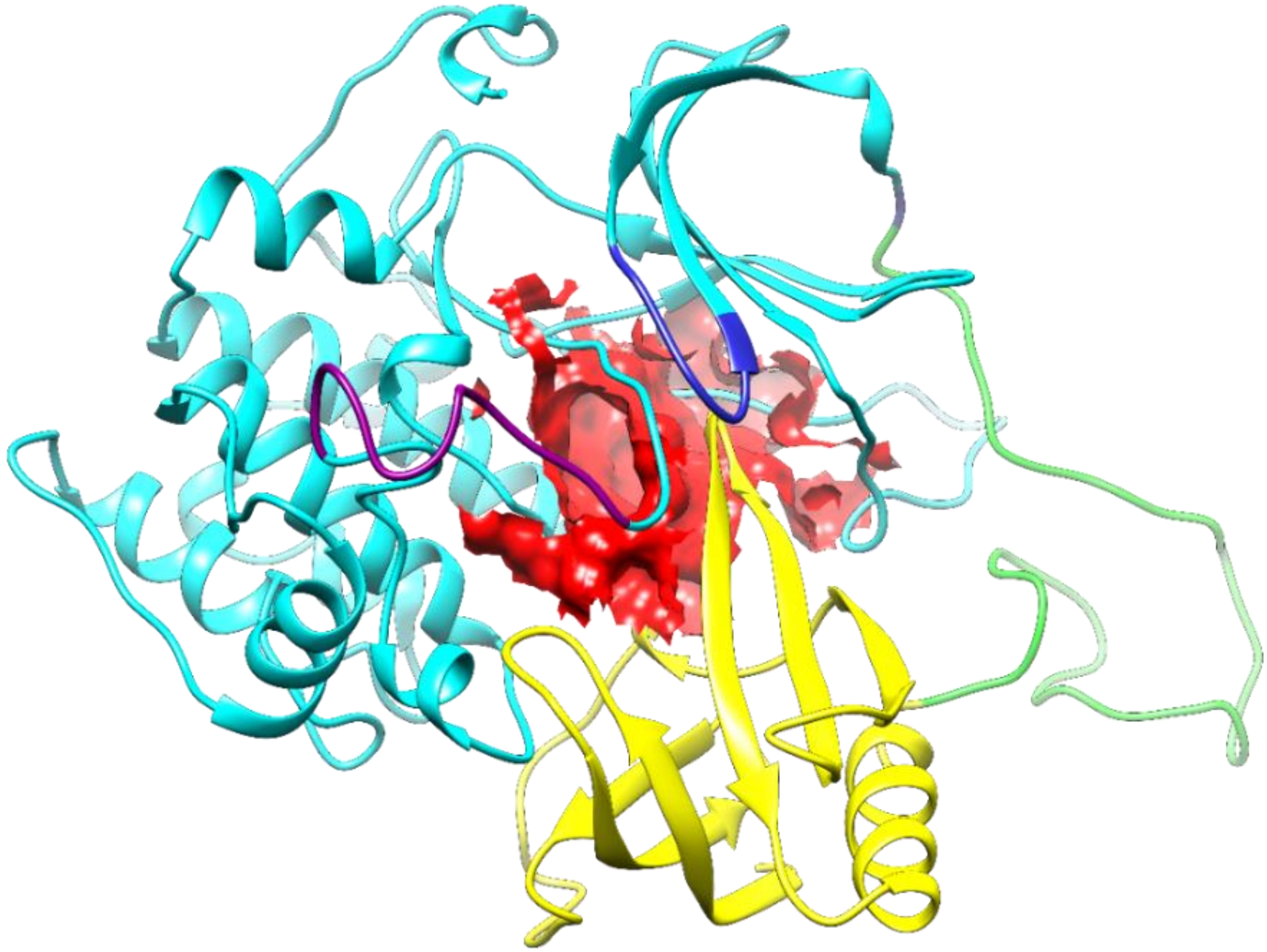

Figure S25. Structure of Protein Ox_2R. The seven transmembrane helices and the horizontal eighth helix are depicted in various colors. The red color represents the surface for the active site.

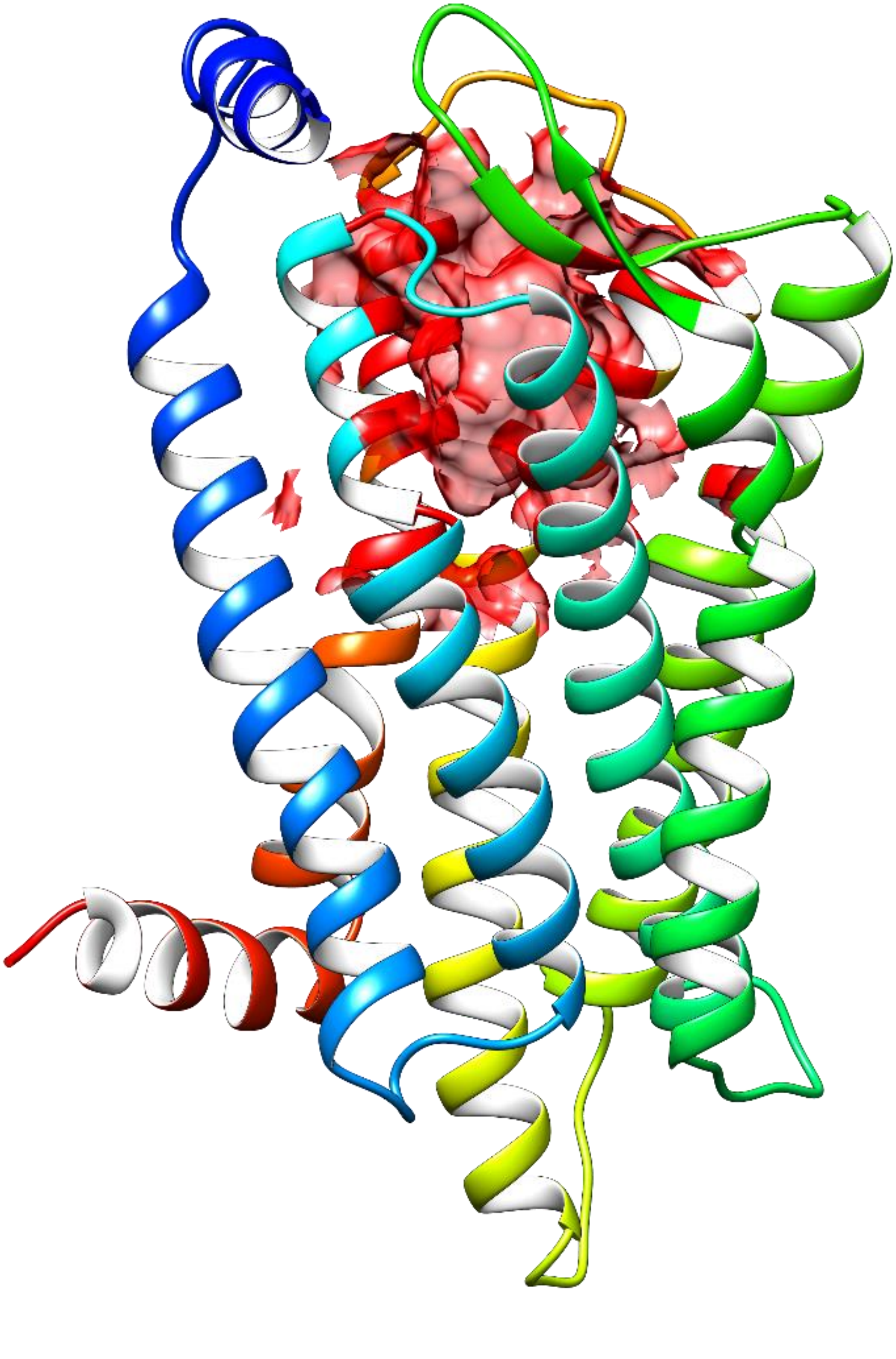

Figure S26. Superposition of Representative Structures from Simulations with Ligands **1f**, **1h**, and **1k**. Changes in helices TM5, TM6, and TM7 can be observed.

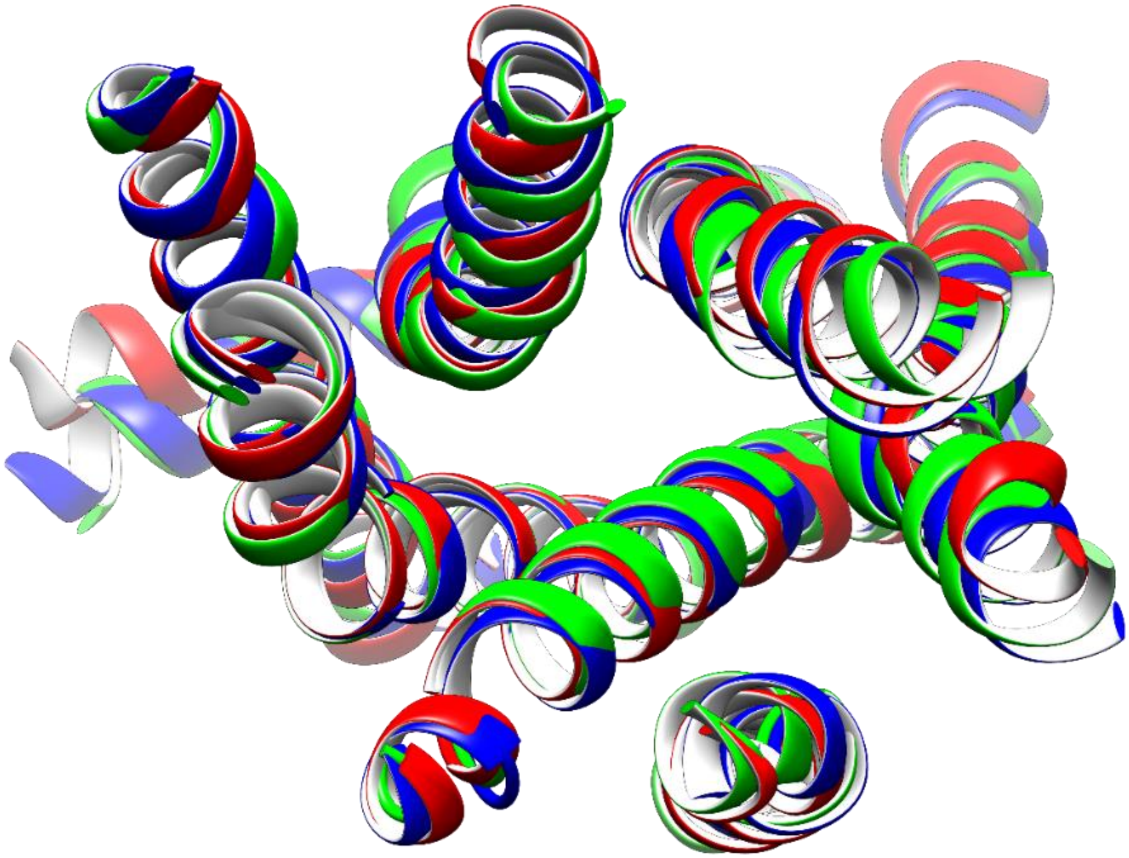

Figure S27. Blind Docking for Structures 5IU2 and 7NA8. The endogenous ligand within the active site is shown with a 97.00% (RMSD = 3.18) population for 5IU2 and 97.74% (RMSD = 5.57) among 1500 independent dockings within the specified active site for this protein.

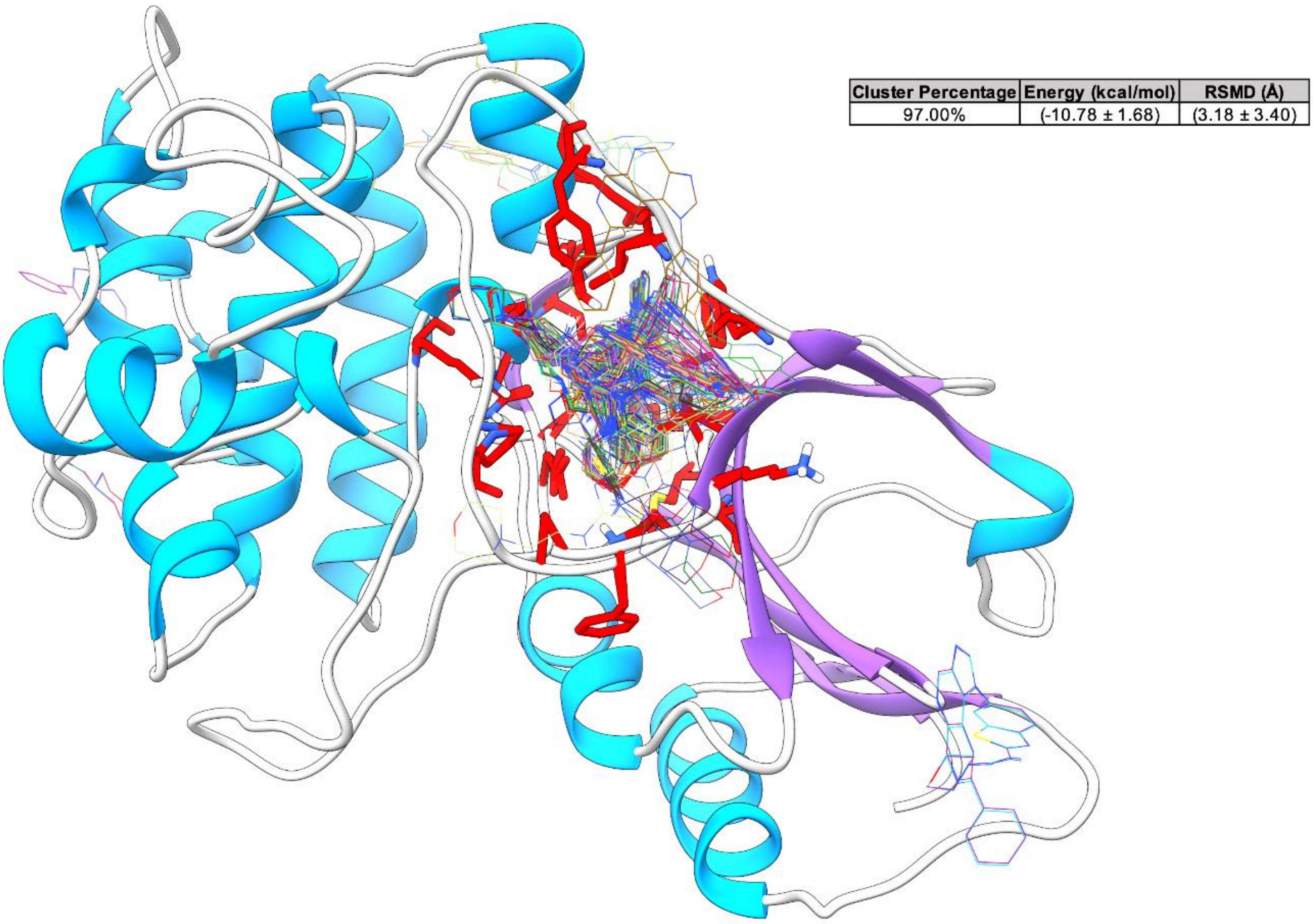

Cluster Percentage	Energy (kcal/mol)	RSMD (Å)
97.00%	(-10.78 ± 1.68)	(3.18 ± 3.40)

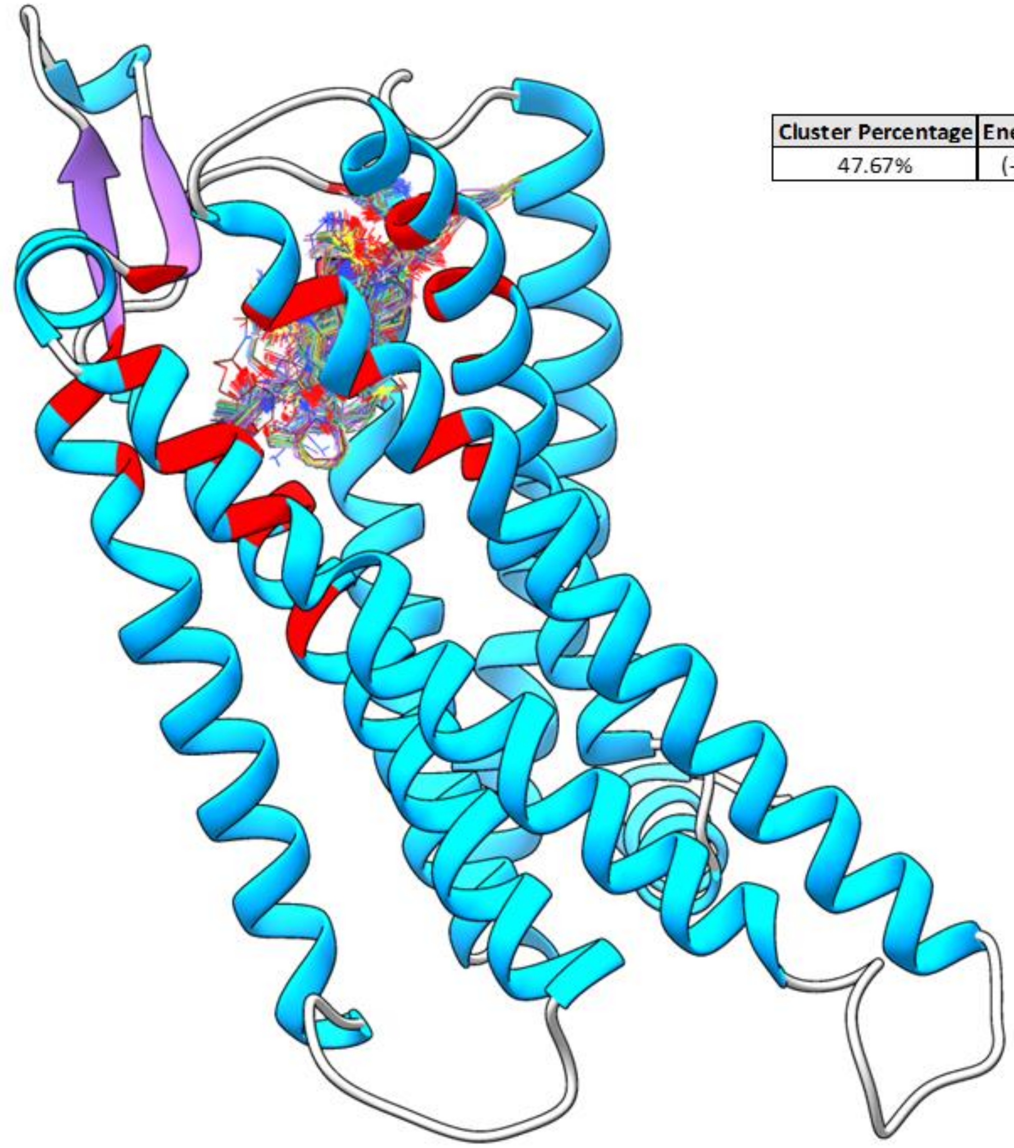

Cluster Percentage	Energy (Kcal/mol)	RMSD (Å)
47.67%	(-10.45 ± 0.40)	(5.57 ± 0.03)

Movie 1. Molecular Simulation of the AKT_1 Target Protein with Ligand **1f**. In this simulation, the linking loop is initially observed to be uncoiled and away from the catalytic site. As the simulation progresses, the loop eventually coils back, approaches, and anchors itself to the protein's center. Ligand **1f** within the allosteric site is shown obstructing the loop's anchoring site. While the simulation was conducted in an explicit water environment, the waters were removed to facilitate the visualization of the linking loop's movement between the kinase and Ph domains. The protein AKT_1 is represented in purple/yellow ribbons, and the ligand is represented as spheres.

https://drive.google.com/file/d/1ZS52LhqK_uTNsacDJWMA2V44yNeYZwa4/view?usp=sharing

Movie 2. Molecular Simulation of the Ox_2R Target Protein with Ligand **1f**. This simulation displays the complete system, including water (liquid) in cyan, lipids in gray, the protein in purple/yellow ribbons, and the ligand in cyan spheres within the active site. The ligand's movements are observed, and it does not undergo significant structural changes, remaining anchored in the active site of the Ox_2R receptor.

https://drive.google.com/file/d/1ApBgUpEaQPDrsO-mouB-wKyzFvhr3Jn9/view?usp=sharing

5 Supplementary References

[S1] Morales-Salazar, I.; Montes-Enríquez, F.P.; Garduño-Albino, C.E.; García-Sánchez, M.A.; Ibarra, I.A.; Rojas-Aguirre, Y.; García-Hernández, M.E.; Sarmiento-Silva, R.E.; Alcaraz-Estrada, S.L.; Díaz-Cervantes, E.; González-Zamora, E.; Islas-Jácome, A. Synthesis of bis-furyl-pyrrolo[3,4-*b*]pyridin-5-ones *via* Ugi–Zhu reaction and *in vitro* activity assays against human SARS-CoV-2 and *in silico* studies on its main proteins. *RSC Med. Chem.* **2023**, *14*, 154–165. DOI: 10.1039/d2md00350c.

[S2] Morales-Salazar, I.; Rincón-Guevara, M.A.; González-Zamora, E.; Islas-Jácome, A. 2-Benzyl-3-morpholino-7-(thiophen-2-yl)-6-(thiophen-2-ylmethyl)-6,7-dihydro-5*H*-pyrrolo[3,4-*b*]pyridin-5-one. *Molbank* **2022**, *4*, M1503. DOI: 10.3390/M1503.
